# Supplementary material for: Glutaminolysis provides nucleotides and amino acids to regulate osteoclast differentiation in mice
Source: EMBO Rep. 2024 Sep 13;25(10):4515–41. doi: 10.1038/s44319-024-00255-x (PMC11467445; doi:10.1038/s44319-024-00255-x)
Supplement: Supplementary file 1 — Appendix [file 44319_2024_255_MOESM1_ESM.pdf]

Appendix

Glutaminolysis provides nucleotides and amino acids to regulate osteoclast differentiation in mice

Guoli Hu[1], Yilin Yu[1], Yinshi Ren[2,3], Robert J Tower[4,5], Guo-Fang Zhang[6,7], Courtney M. Karner[1,5,*]

[1] Department of Internal Medicine, University of Texas Southwestern Medical Center, Dallas, TX 75390
[2] Center for Excellence in Hip Disorders, Texas Scottish Rite Hospital for Children, Dallas, TX 75219
[3] Department of Orthopedic Surgery, University of Texas Southwestern Medical Center, Dallas, TX, 75390
[4] Department of Surgery, University of Texas Southwestern Medical Center, Dallas, TX 75390
[5] Charles and Jane Pak Center for Mineral Metabolism and Clinical Research, University of Texas Southwestern Medical Center, Dallas, TX 75390
[6] Department of Medicine, Division of Endocrinology, Metabolism Nutrition, Duke University Medical Center, Durham, NC 27701
[7] Sarah W. Stedman Nutrition and Metabolism Center & Duke Molecular Physiology Institute, Duke University School of Medicine, Durham, NC 27701

[*]**Correspondence:** Courtney.Karner@UTSouthwestern.edu

Table of Contents:

Appendix Figure S1…………………Page 2
Appendix Figure S2…………………Page 3
Appendix Figure S3…………………Page 4
Appendix Figure S4…………………Page 5
Appendix Table S1…………………..Page 6

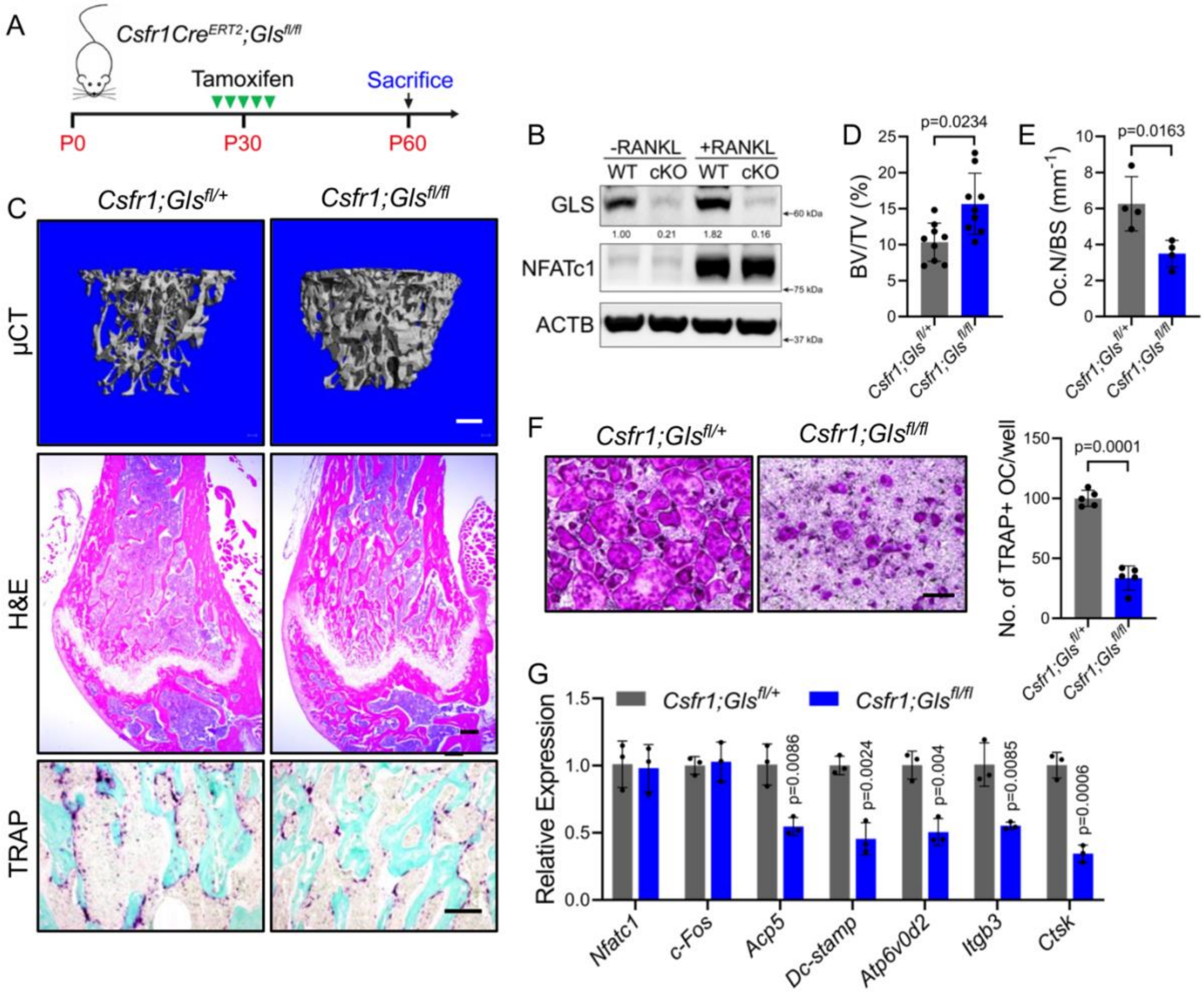

Appendix Figure S1. ***Csfr1Cre^{ERT2};Gls$^{fl/fl}$* mice have increased bone mass due to reduced osteoclast numbers and bone resorption**

(**A**) Schematic of the experimental design. (**B**) Western blot analysis of GLS and NTAFc1 in BMMs derived from WT (*Csfr1Cre^{ERT2};Gls$^{fl/+}$*) and *Csfr1Cre^{ERT2};Gls$^{fl/fl}$* mice stimulated with or without RANKL for 72 hours (*n* = 3). (**C**) Representative µCT (*n* = 9, scale bar: 200 µm), H&E (*n* = 4, scale bar: 100 µm), and TRAP staining (*n* = 4, scale bar: 100 µm) performed on distal femur trabecular bone from 2-month-old WT and *Csfr1;Gls$^{fl/fl}$* female mice. (**D**) The calculated bone volume per tissue volume (BV/TV) (*n* = 9 mice). (**E**) Quantification of osteoclast number per bone surface (Oc.N/BS) (*n* = 4 mcie). (**F**) TRAP staining (left panel) and quantification of TRAP-positive multi-nuclei cells (graph) of BMMs derived from WT or *Csfr1;Gls$^{fl/fl}$* female mice cultured in the presence of RANKL for 4 days (*n* = 5 independent experiments). Scale bar: 500 µm. (**G**) qPCR analysis of osteoclast marker gene expression in BMMs derived from WT and *Csfr1;Gls$^{fl/fl}$* female mice cultured in the presence of RANKL for 4 days (*n* = 3 independent experiments). Data are shown as mean ± SD. 2-tailed Student's paired t test (**D** and **E**). 2-tailed Student's unpaired t test (**F** and **G**).

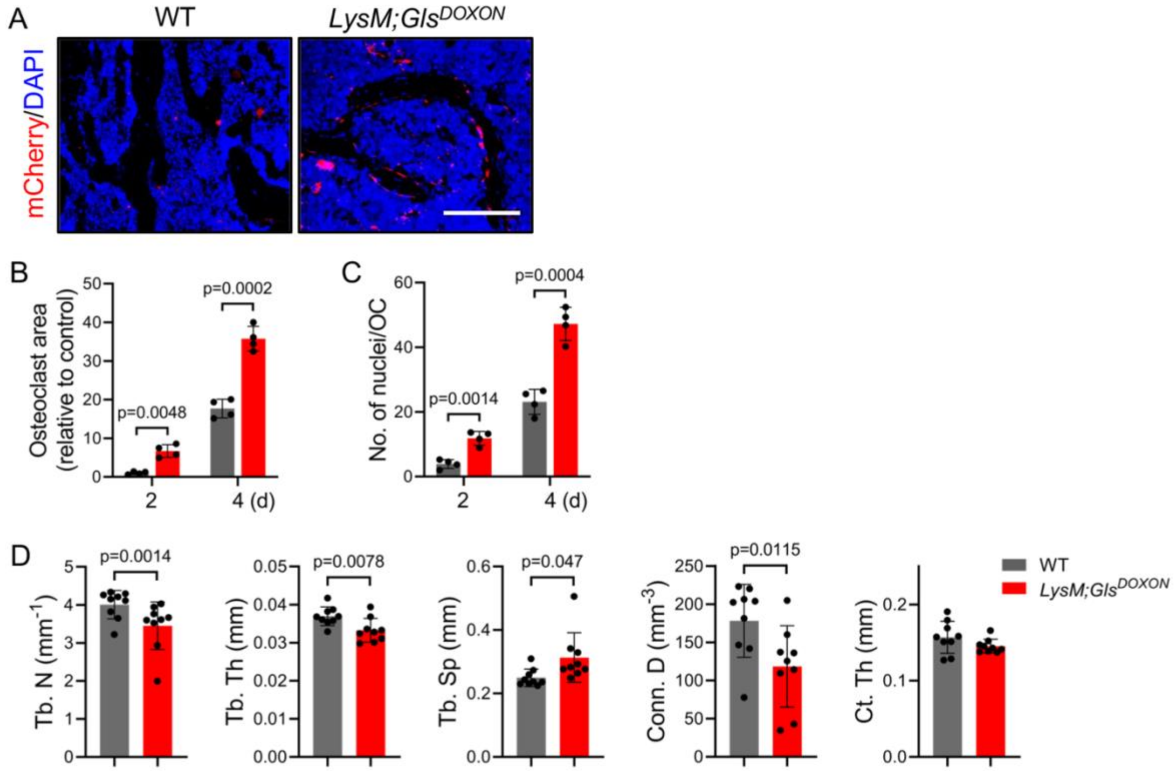

Appendix Figure S2. Confirmation of mCherry expression and microCT parameters of *LysM;Gls^DOXON* mice.

(**A**) mCherry immunofluorescence staining performed on distal femurs from 2-month-old WT and *LysM;Gls^DOXON* female mice (*n* = 4). Scale bar: 100 μm. (**B**-**C**) Quantification of TRAP+ osteoclast area (**B**) and the average number of nuclei per osteoclast (**C**) (n = 4 independent experiments). (**D**) μCT parameters of trabecular bone in the distal femurs of 2-month-old WT and *LysM;Gls^DOXON* female mice (n = 9 mice). Data are shown as mean ± SD. 2-tailed Student's unpaired t test (**B** and **C**). 2-tailed Student's paired t test (**D**).

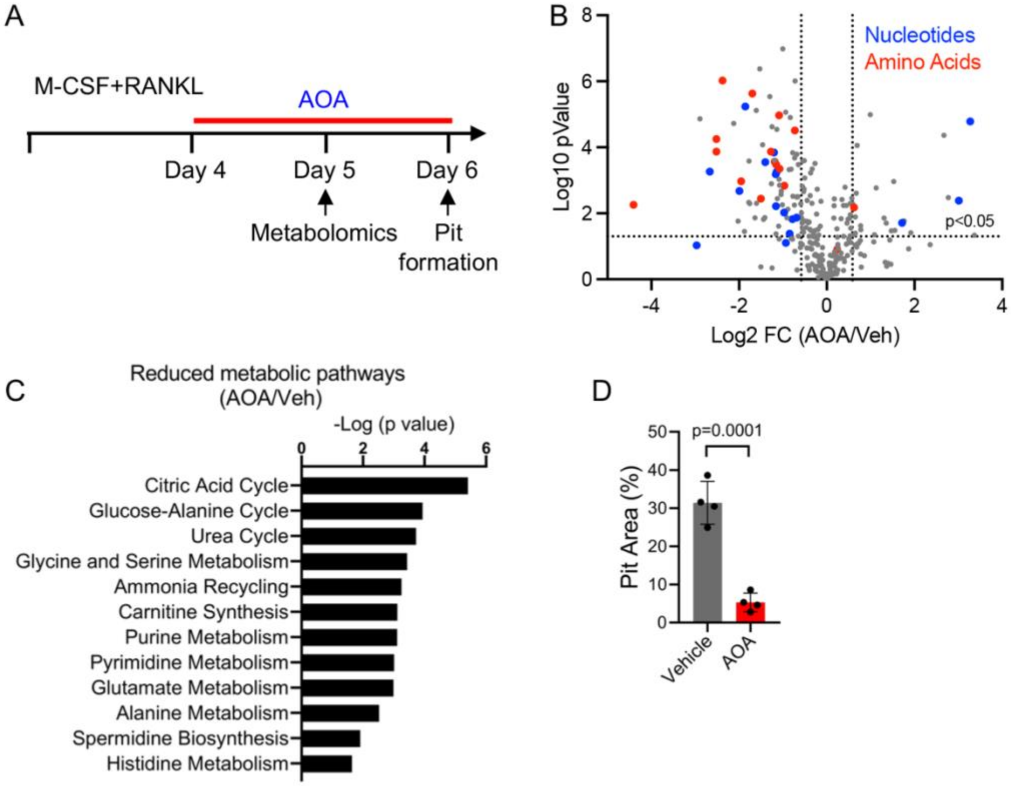

Appendix Figure S3. Metabolic changes caused by pan-transaminase inhibition.
(**A**) Schematic depicting the experimental design used for metabolomics and pit formation assay. (**B**) Volcano plot showing metabolites that differed significantly between AOA (200 μM)- and vehicle-treated mOC (*n* = 4). (**C**) Metabolic pathway analysis showing reduced pathways in AOA-treated mOC. (**D**) Quantification of the resorption pit area of **Fig. 7K** (*n* = 4 independent cultures). Data are shown as mean ± SD. 2-tailed Student's unpaired t test (**B**-**D**).

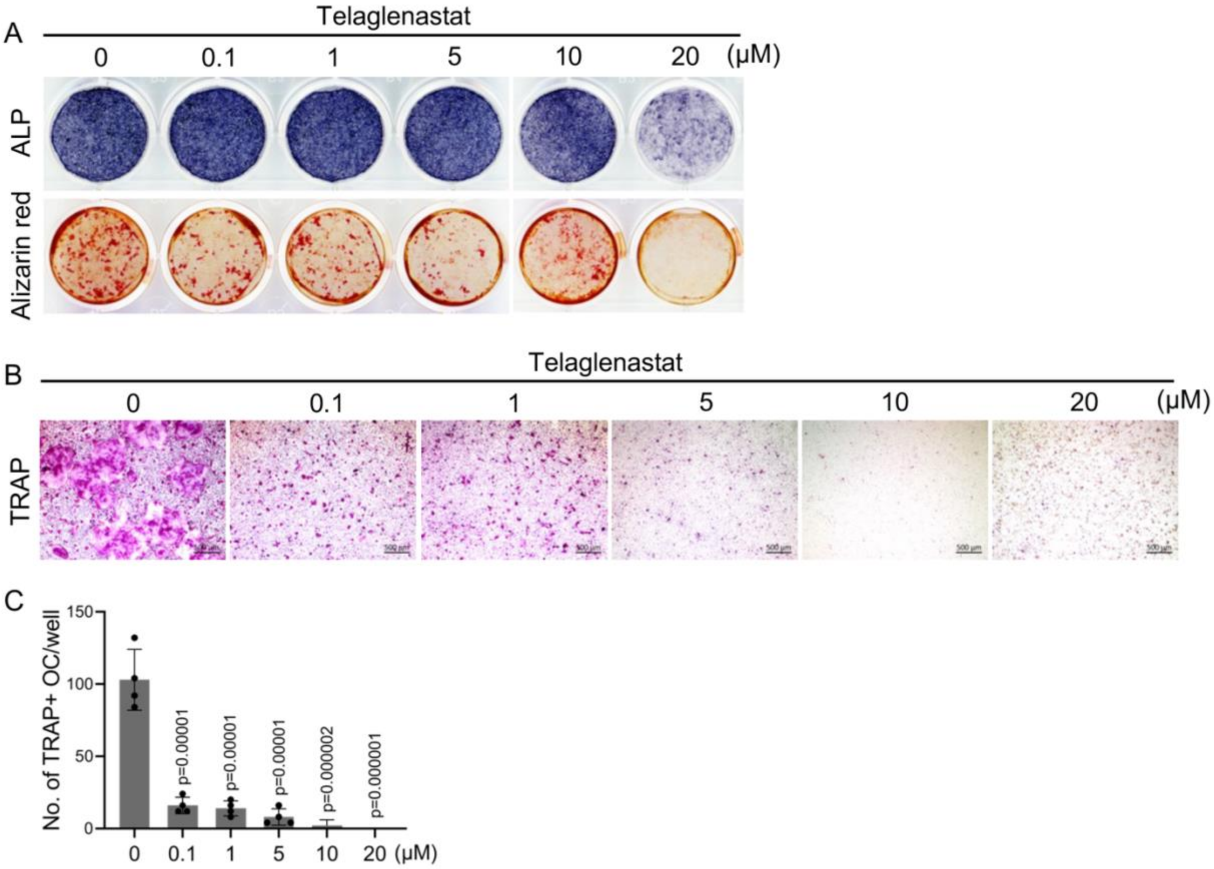

Appendix Figure S4. Sensitivity to CB-839 in osteoblast and osteoclast cultures.
(**A**) ALP staining and Alizarin red staining showing the effects of a gradient of CB-839 on osteoblast differentiation of primary calvarial osteoblast precursor cells (*n* = 4). (**B**) TRAP staining showing the effects of a gradient of CB-839 on the formation of multinucleated TRAP positive osteoclasts (*n* = 4). Scale bar: 500 μm. (**C**) Quantification of the number of TRAP-positive multinucleated osteoclasts from **B** (*n* = 4 independent cultures). Data are shown as mean ± SD. 1-way ANOVA (**C**).

Appendix Table S1. List of qPCR primers used in this study.

Gene symbol	Forward	Reverse
Nfatc1	GGAGCGGAGAAACTTTGCG	GTGACACTAGGGGACACATAACT
c-Fos	CGGGTTTCAACGCCGACTA	TTGGCACTAGAGACGGACAGA
Acp5	CACTCCCACCCTGAGATTTGT	CATCGTCTGCACGGTTCTG
Dc-stamp	TACGTGGAGAGAAGCAAGGAA	ACACTGAGACGTGGTTTAGGAAT
Itgb3	CCACACGAGGCGTGAACTC	CTTCAGGTTACATCGGGGTGA
Atp6v0d2	CAGAGCTGTACTTCAATGTGGAC	AGGTCTCACACTGCACTAGGT
Ctsk	GAAGAAGACTCACCAGAAGCAG	TCCAGGTTATGGGCAGAGATT
β-actin	AGATGTGGATCAGCAAGCAG	GCGCAAGTTAGGTTTTGTCA
