# Supplementary figures and images for: Glutaminolysis provides nucleotides and amino acids to regulate osteoclast differentiation in mice
Source: EMBO Rep. 2024 Sep 13;25(10):4515–41. doi: 10.1038/s44319-024-00255-x (PMC11467445; doi:10.1038/s44319-024-00255-x)

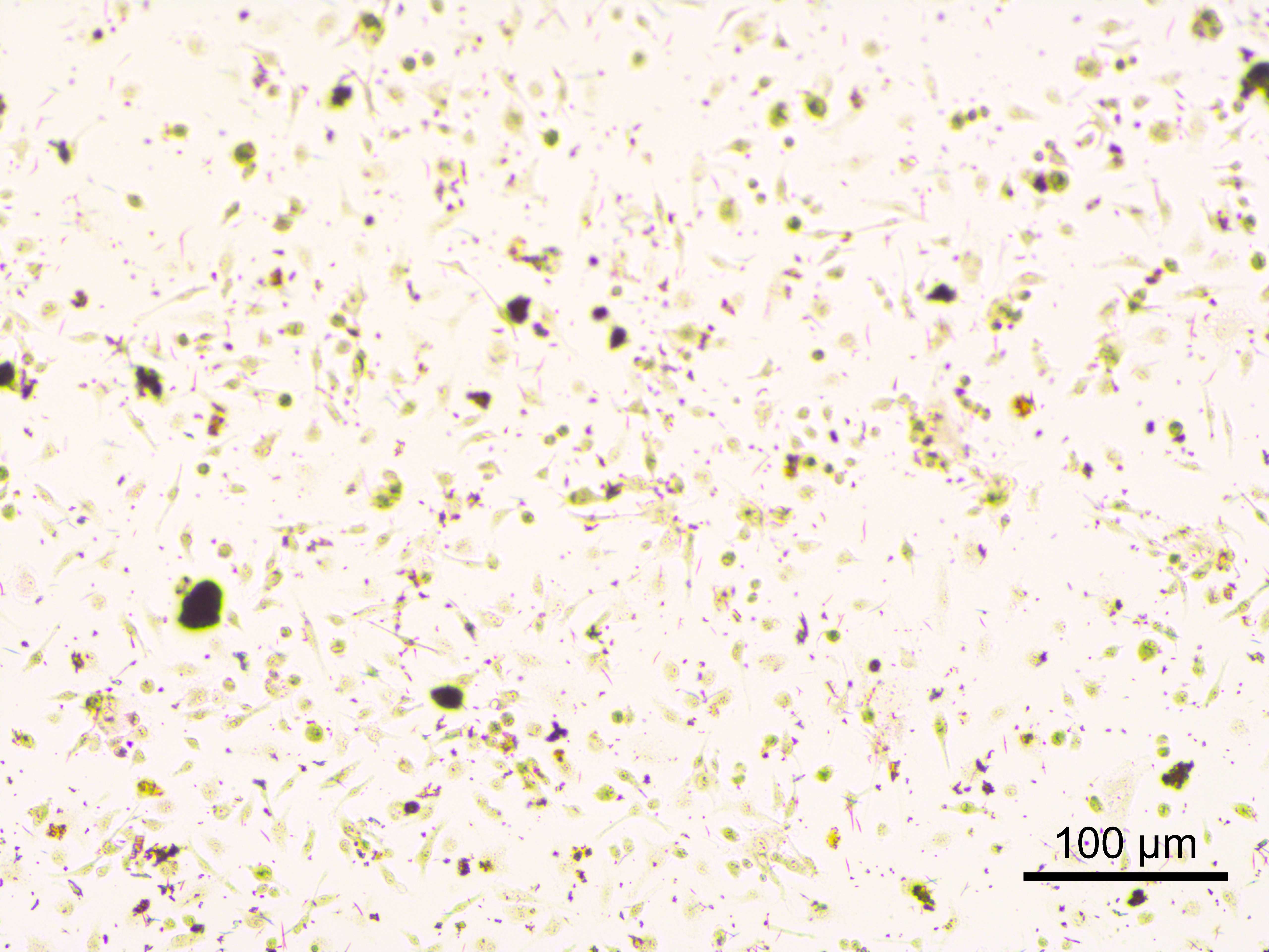

Supplement: Supplementary file 3 — Source data Fig. 1 [file 44319_2024_255_MOESM3_ESM.zip › Figure 1/1B/TRAP stain_BMM.tif]

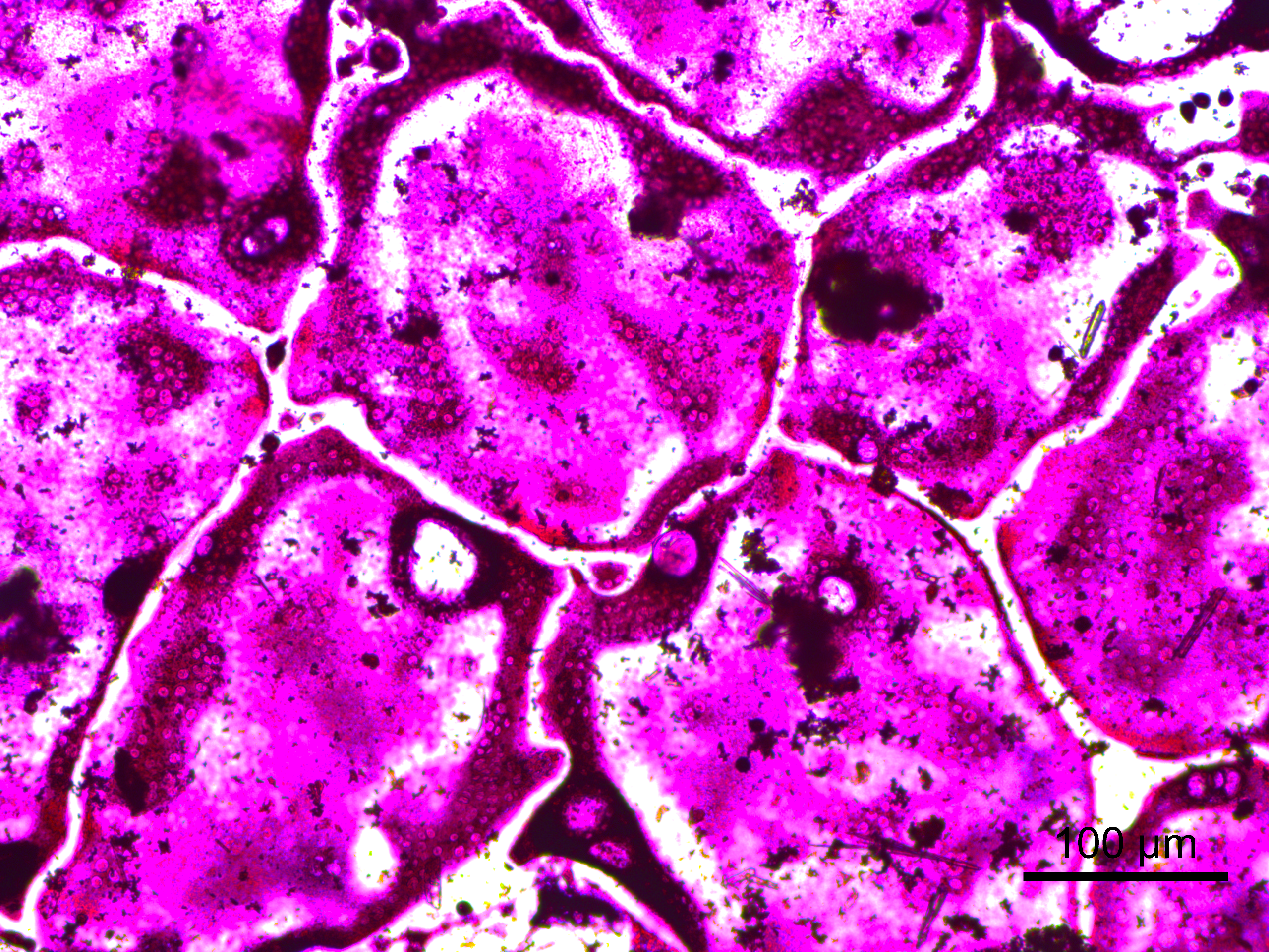

Supplement: Supplementary file 3 — Source data Fig. 1 [file 44319_2024_255_MOESM3_ESM.zip › Figure 1/1B/TRAP stain_mOC.tif]

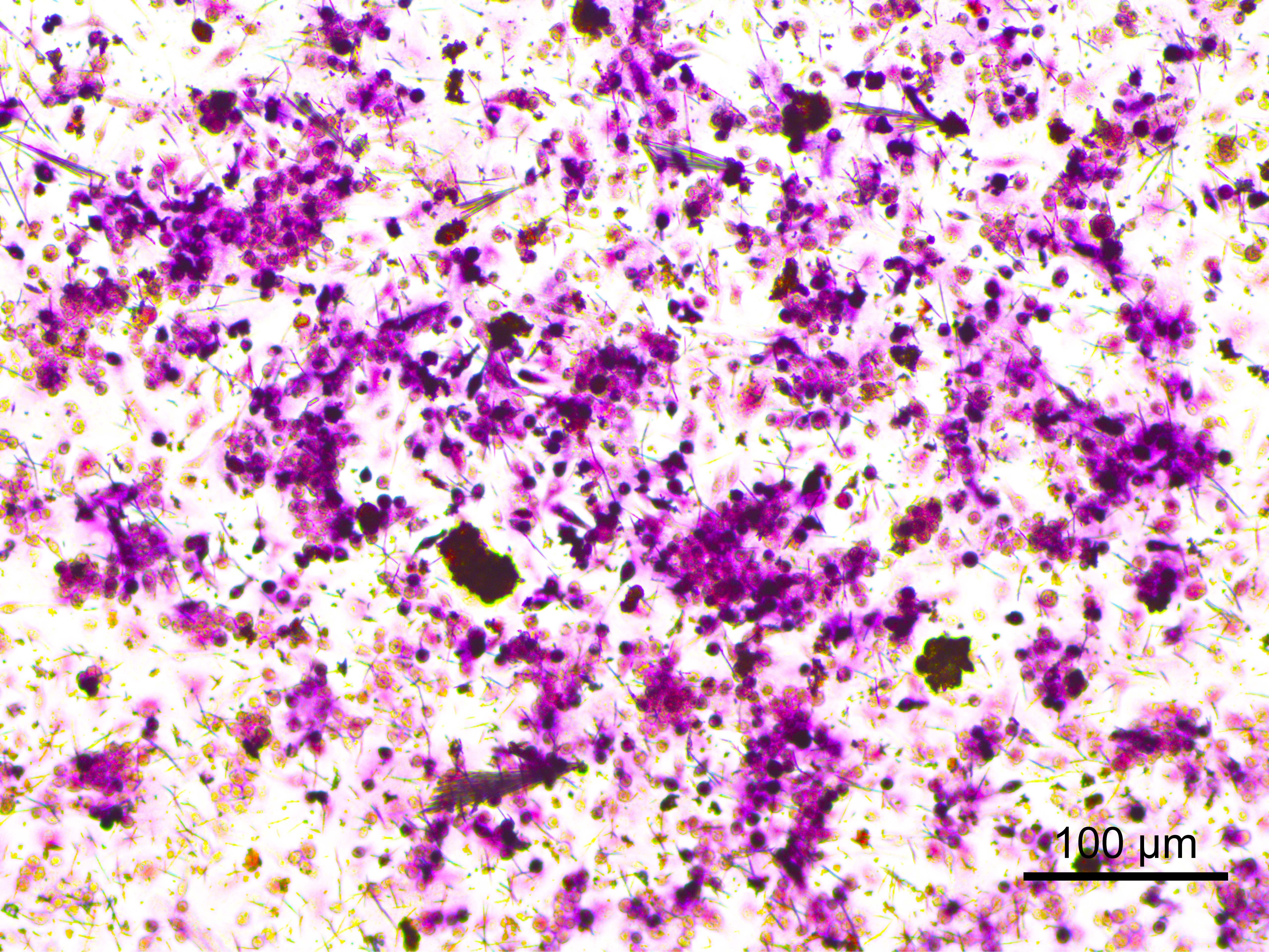

Supplement: Supplementary file 3 — Source data Fig. 1 [file 44319_2024_255_MOESM3_ESM.zip › Figure 1/1B/TRAP stain_pOC.tif]

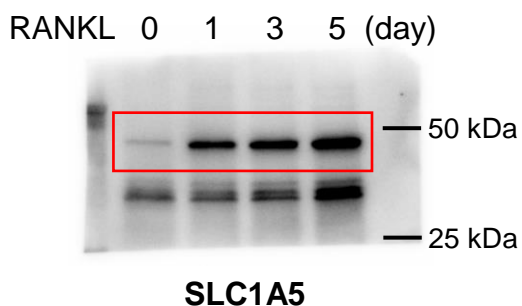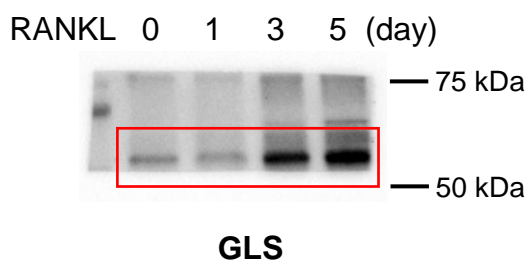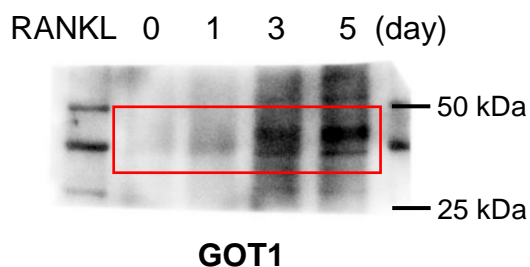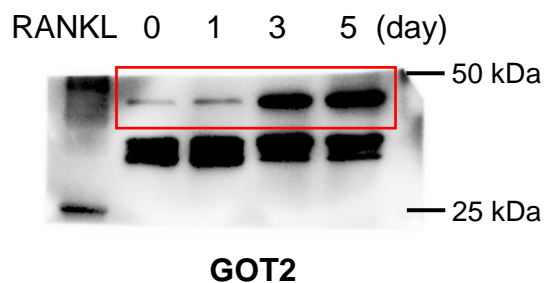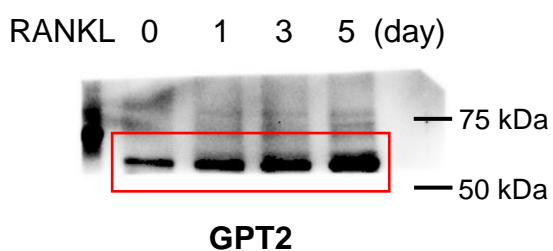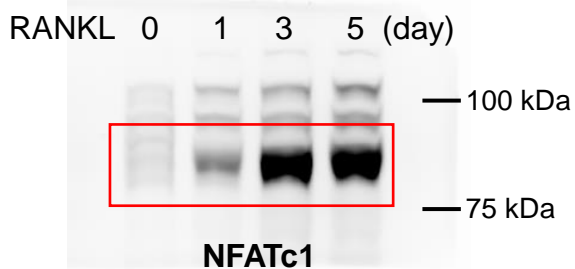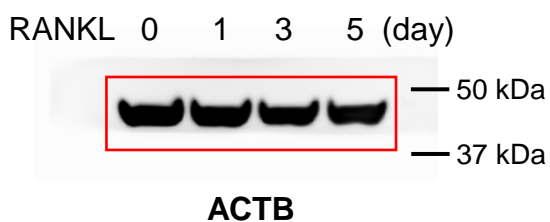

**Full unedited gel for Figure 2A**

Supplement: Supplementary file 4 — Source data Fig. 2 [file 44319_2024_255_MOESM4_ESM.zip › Figure 2/2A/Full unedited gels for 2A.pdf]

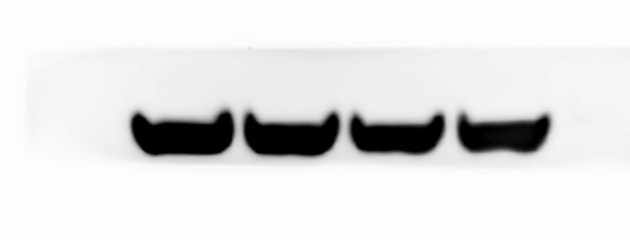

Supplement: Supplementary file 4 — Source data Fig. 2 [file 44319_2024_255_MOESM4_ESM.zip › Figure 2/2A/western blot_ACTB.tif]

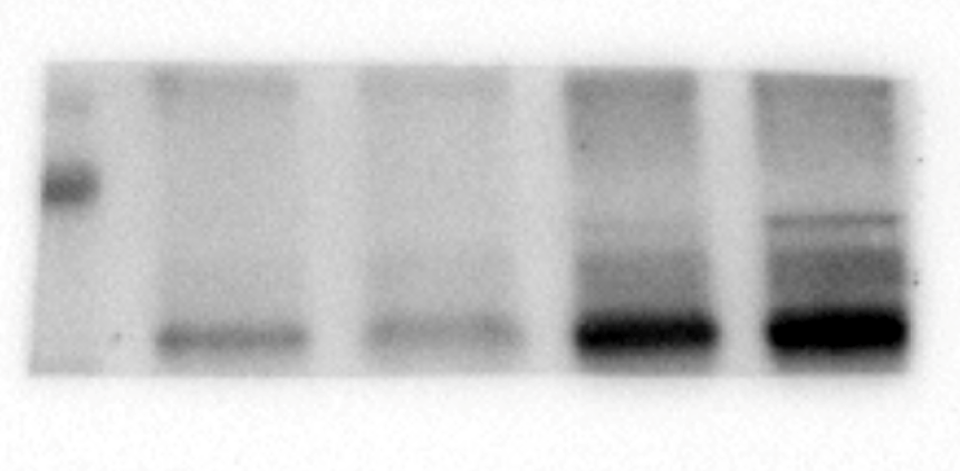

Supplement: Supplementary file 4 — Source data Fig. 2 [file 44319_2024_255_MOESM4_ESM.zip › Figure 2/2A/western blot_GLS.tif]

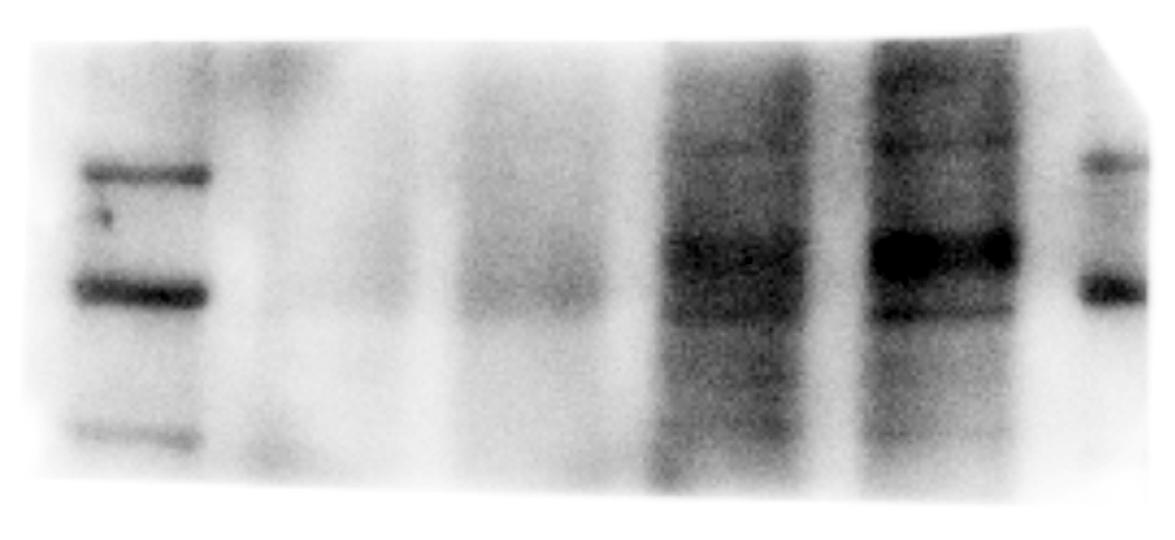

Supplement: Supplementary file 4 — Source data Fig. 2 [file 44319_2024_255_MOESM4_ESM.zip › Figure 2/2A/western blot_GOT1.tif]

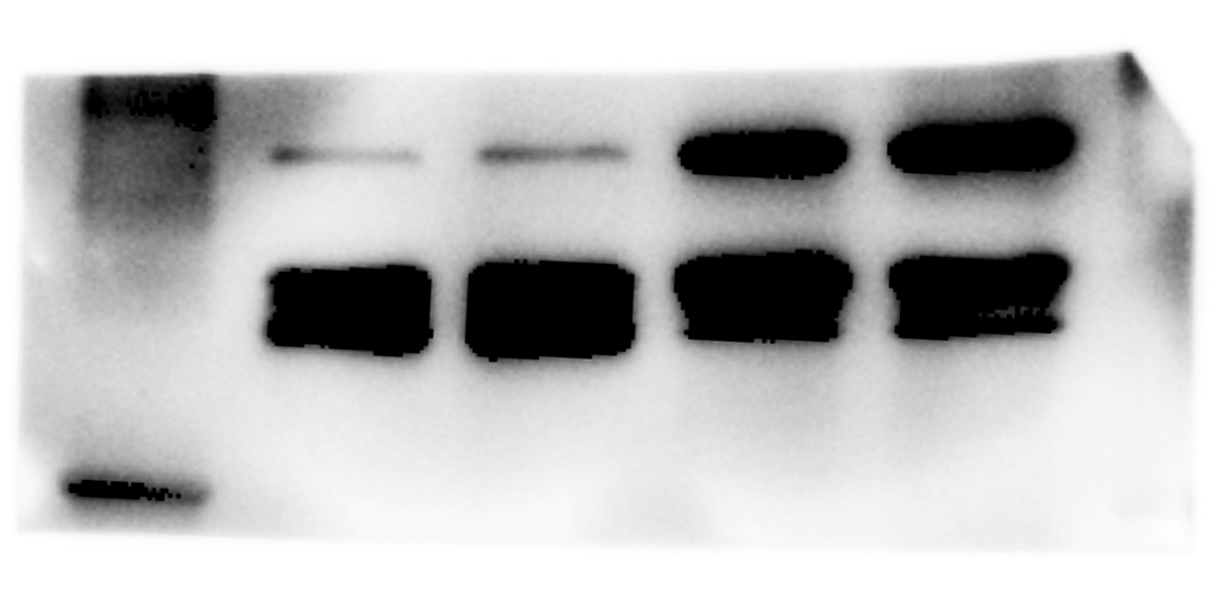

Supplement: Supplementary file 4 — Source data Fig. 2 [file 44319_2024_255_MOESM4_ESM.zip › Figure 2/2A/western blot_GOT2.tif]

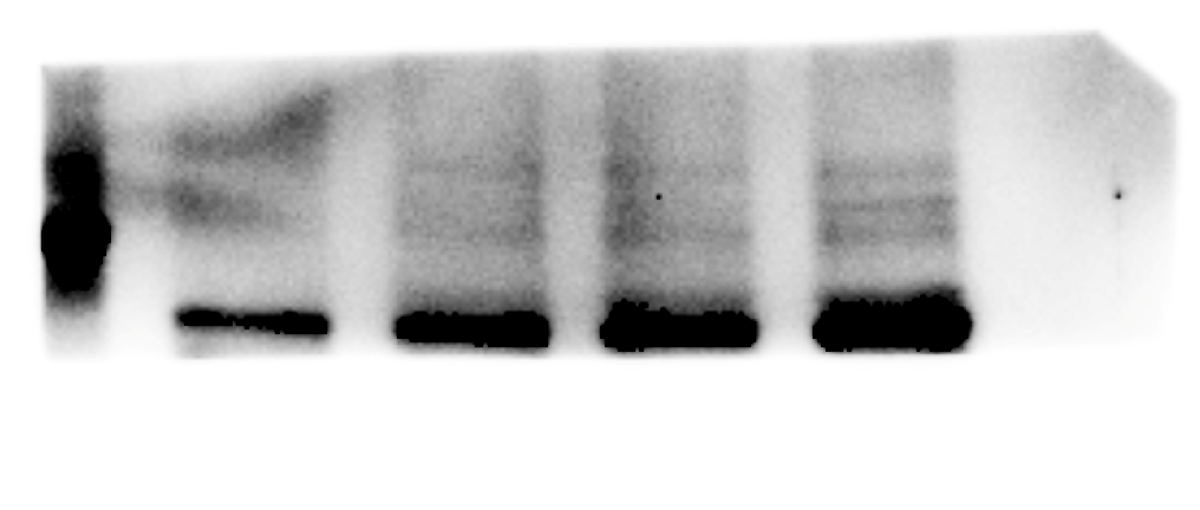

Supplement: Supplementary file 4 — Source data Fig. 2 [file 44319_2024_255_MOESM4_ESM.zip › Figure 2/2A/western blot_GPT2.tif]

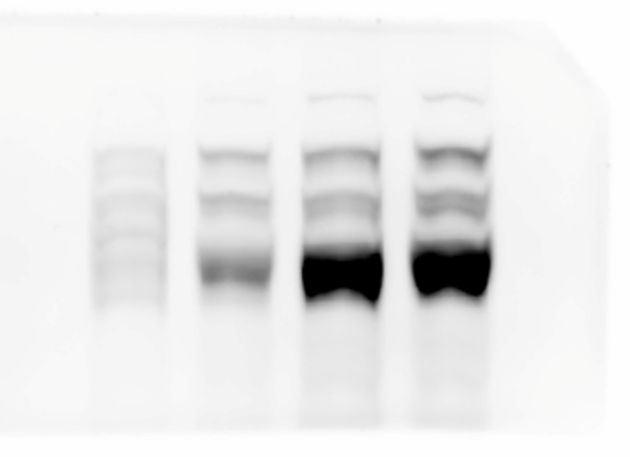

Supplement: Supplementary file 4 — Source data Fig. 2 [file 44319_2024_255_MOESM4_ESM.zip › Figure 2/2A/western blot_NFATC1.tif]

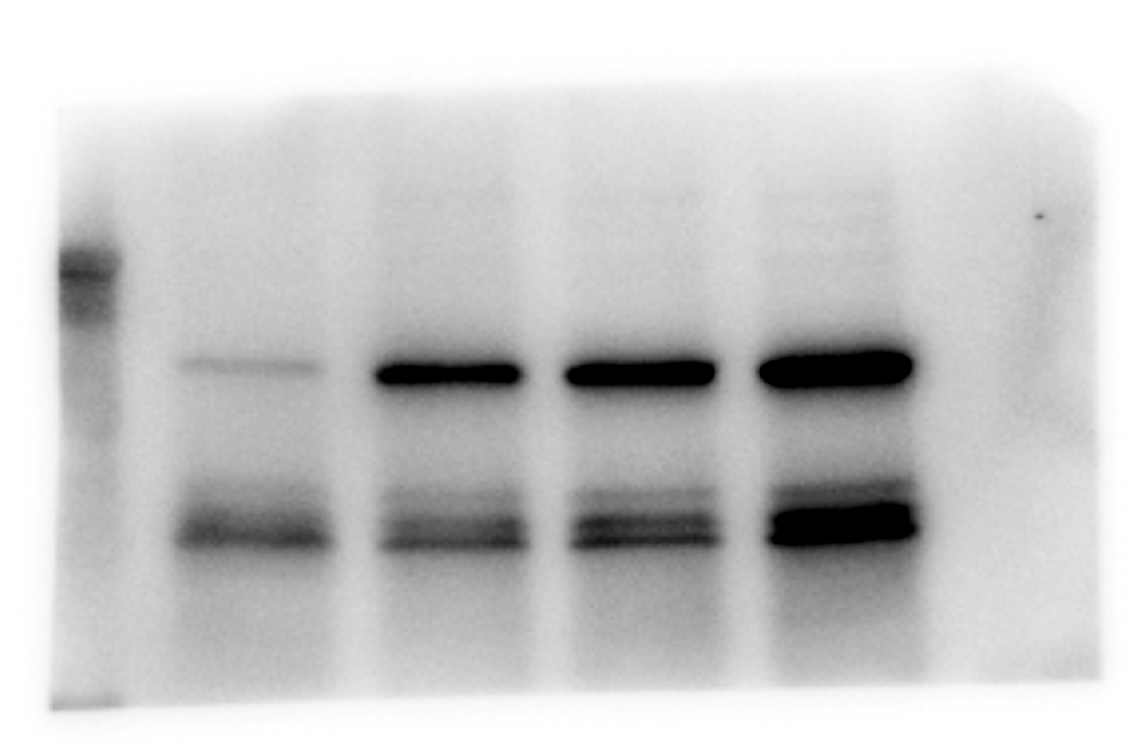

Supplement: Supplementary file 4 — Source data Fig. 2 [file 44319_2024_255_MOESM4_ESM.zip › Figure 2/2A/western blot_SLC1A5.tif]

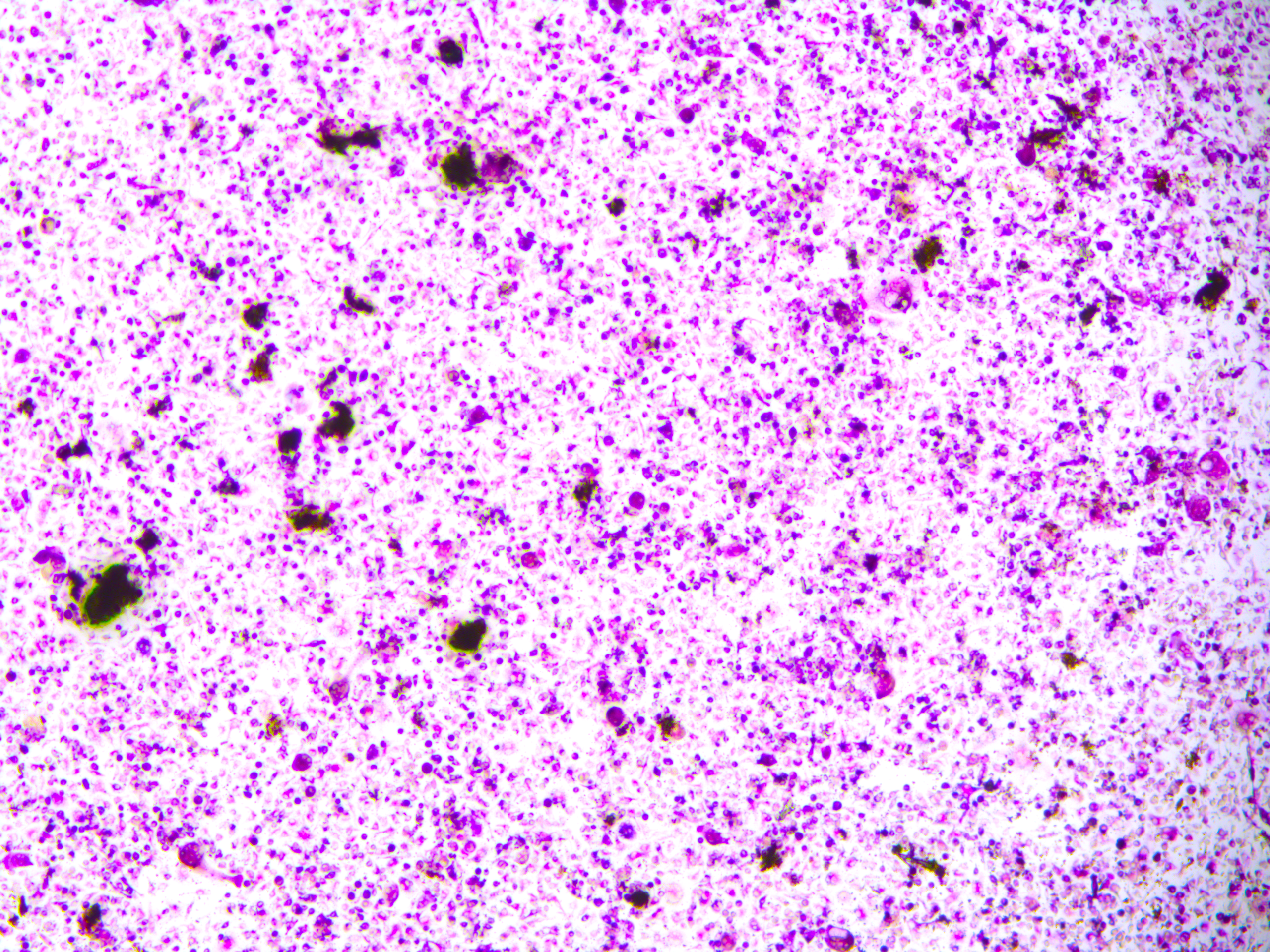

Supplement: Supplementary file 5 — Source data Fig. 3 [file 44319_2024_255_MOESM5_ESM.zip › Figure 3/3B/TRAP_BPTES.tif]

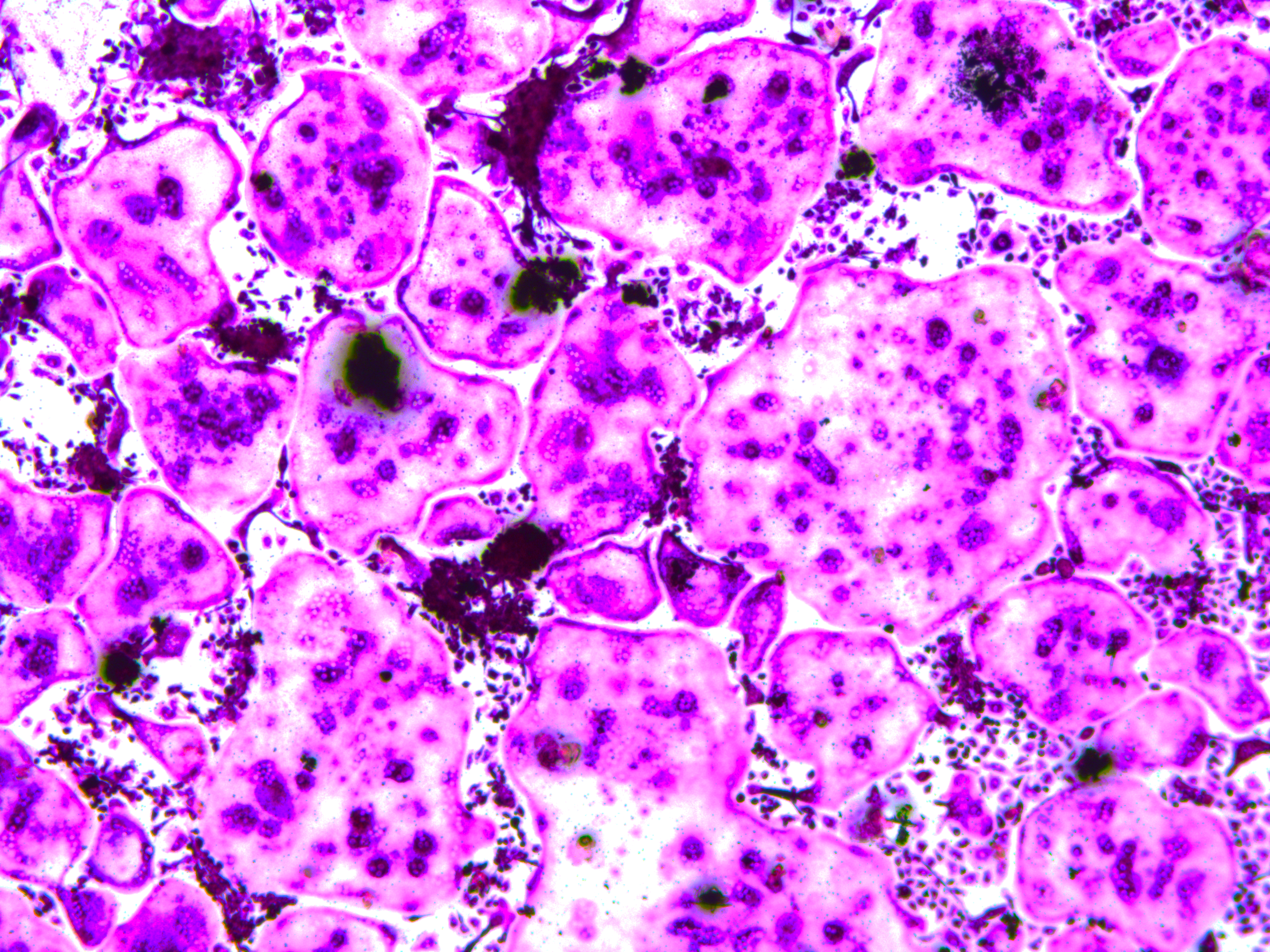

Supplement: Supplementary file 5 — Source data Fig. 3 [file 44319_2024_255_MOESM5_ESM.zip › Figure 3/3B/TRAP_vehicle.tif]

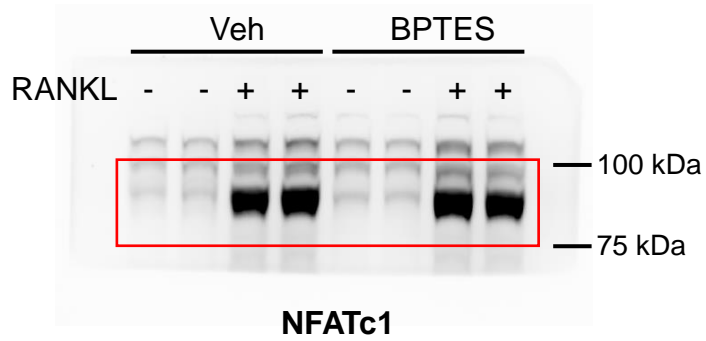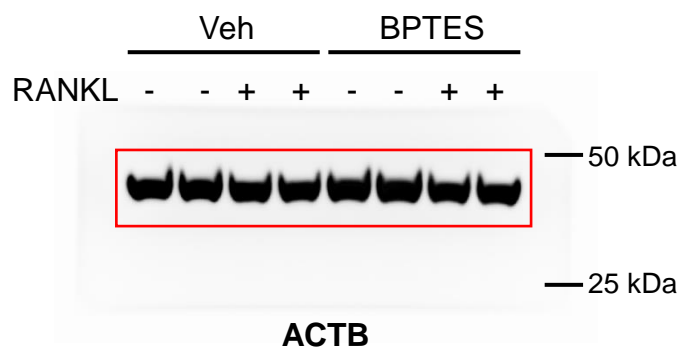

Full unedited gel for Fig. 3C

Supplement: Supplementary file 5 — Source data Fig. 3 [file 44319_2024_255_MOESM5_ESM.zip › Figure 3/3C/Full unedited gel for 3C.pdf]

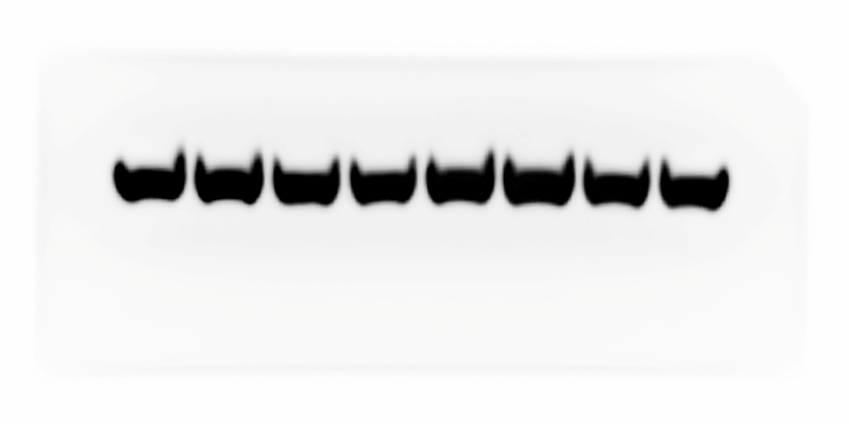

Supplement: Supplementary file 5 — Source data Fig. 3 [file 44319_2024_255_MOESM5_ESM.zip › Figure 3/3C/western blot_ACTB.tif]

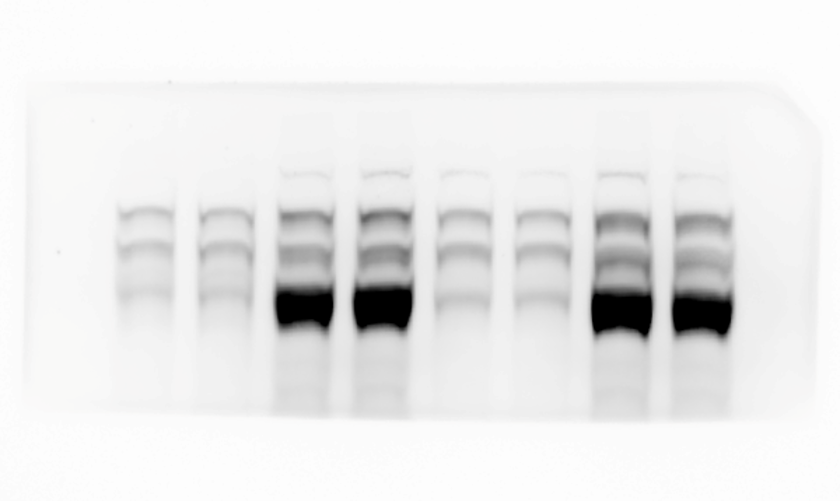

Supplement: Supplementary file 5 — Source data Fig. 3 [file 44319_2024_255_MOESM5_ESM.zip › Figure 3/3C/western blot_NFATc1.tif]

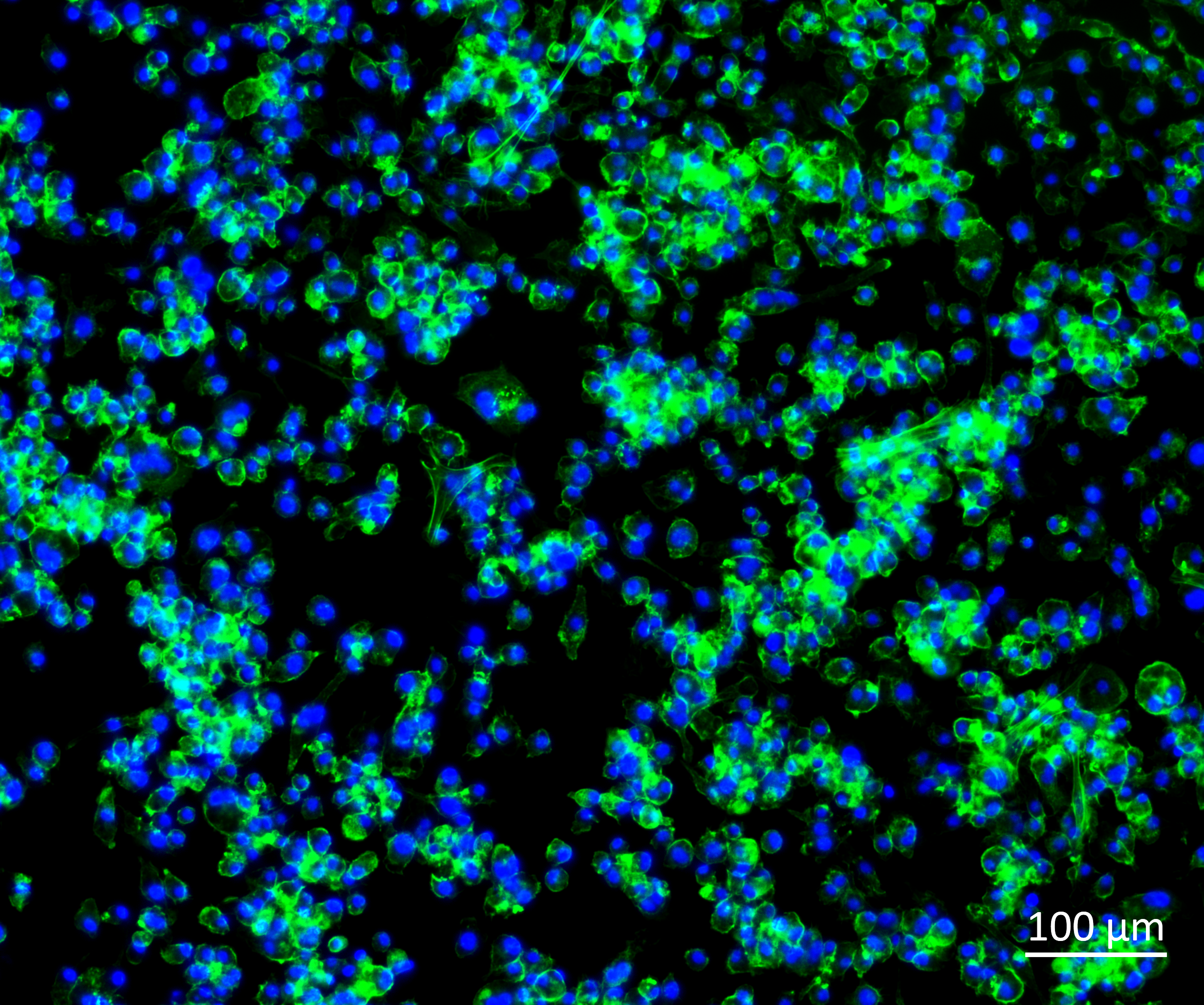

Supplement: Supplementary file 5 — Source data Fig. 3 [file 44319_2024_255_MOESM5_ESM.zip › Figure 3/3E/actin ring_BPTES.tif]

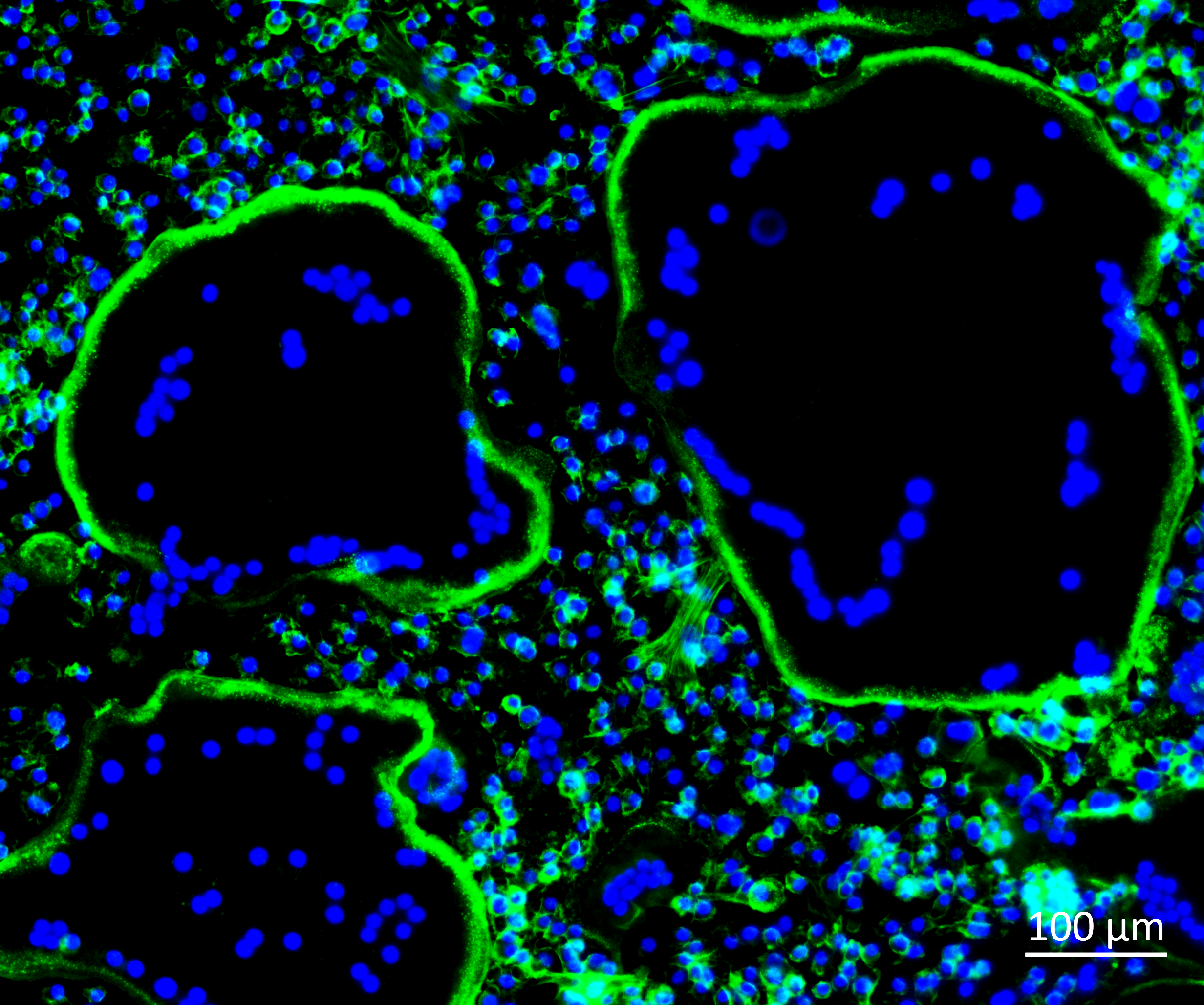

Supplement: Supplementary file 5 — Source data Fig. 3 [file 44319_2024_255_MOESM5_ESM.zip › Figure 3/3E/actin ring_vehicle.tif]

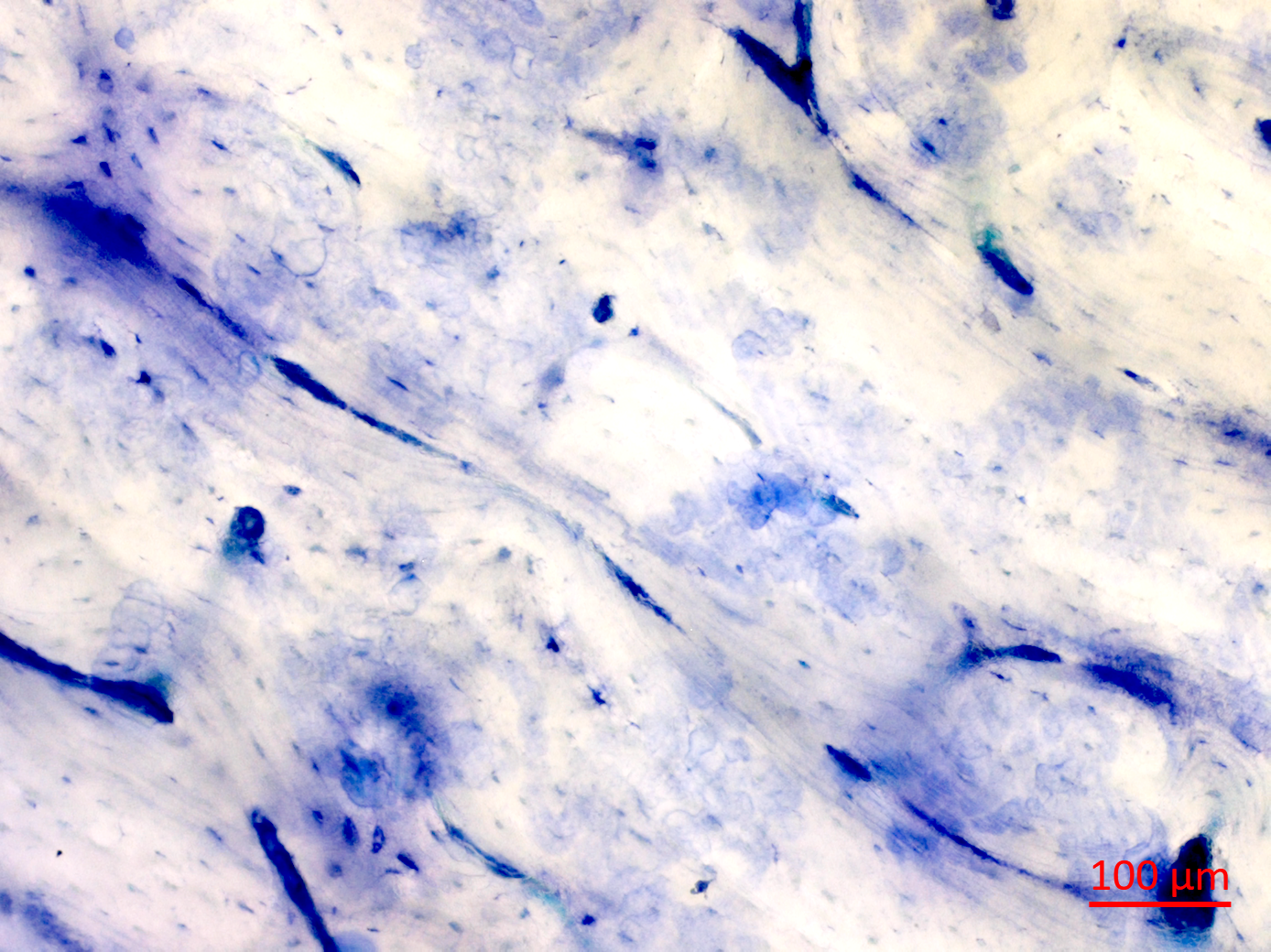

Supplement: Supplementary file 5 — Source data Fig. 3 [file 44319_2024_255_MOESM5_ESM.zip › Figure 3/3F/pit assay_BPTES.tif]

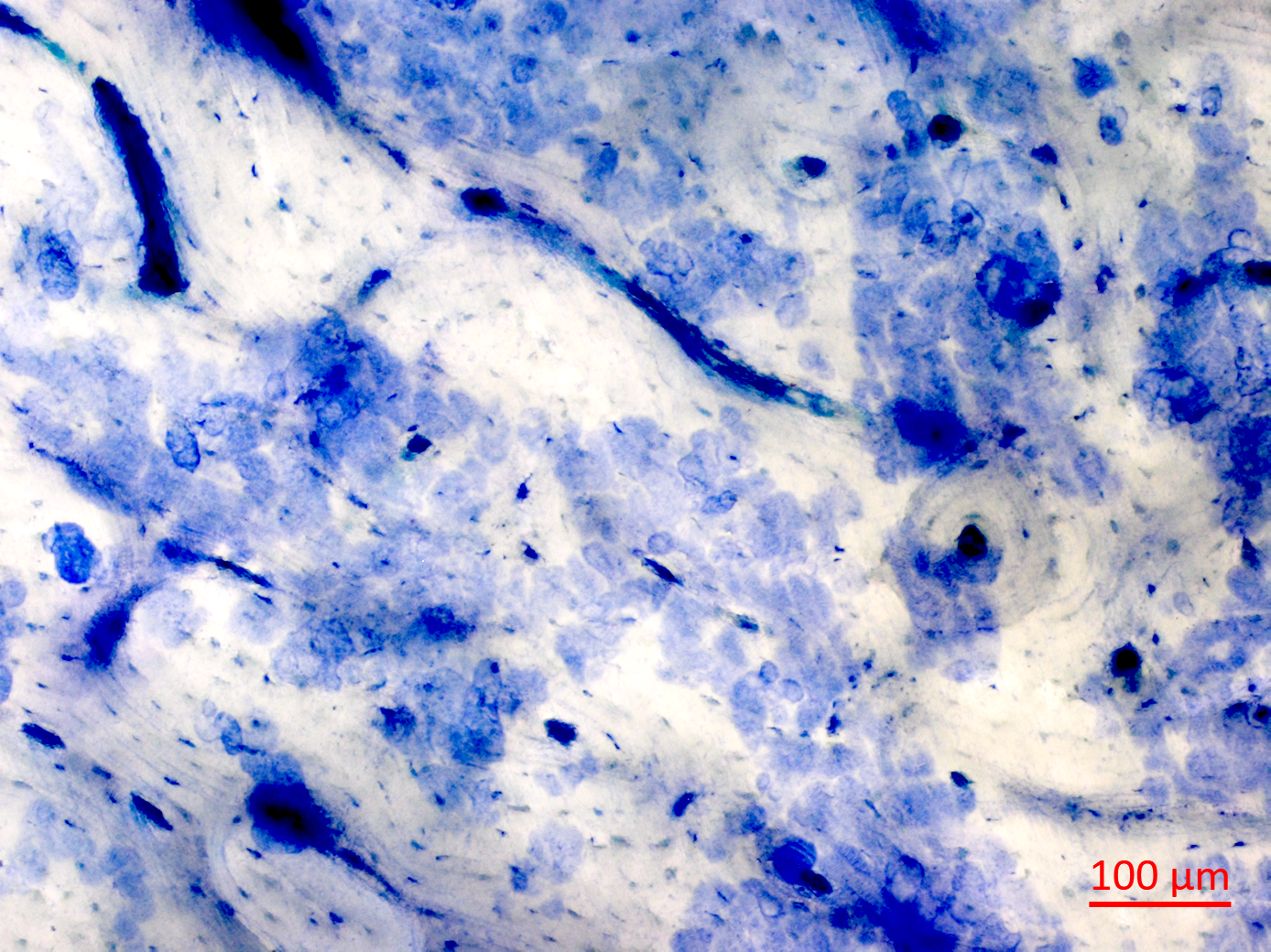

Supplement: Supplementary file 5 — Source data Fig. 3 [file 44319_2024_255_MOESM5_ESM.zip › Figure 3/3F/pit assay_vehicle.tif]

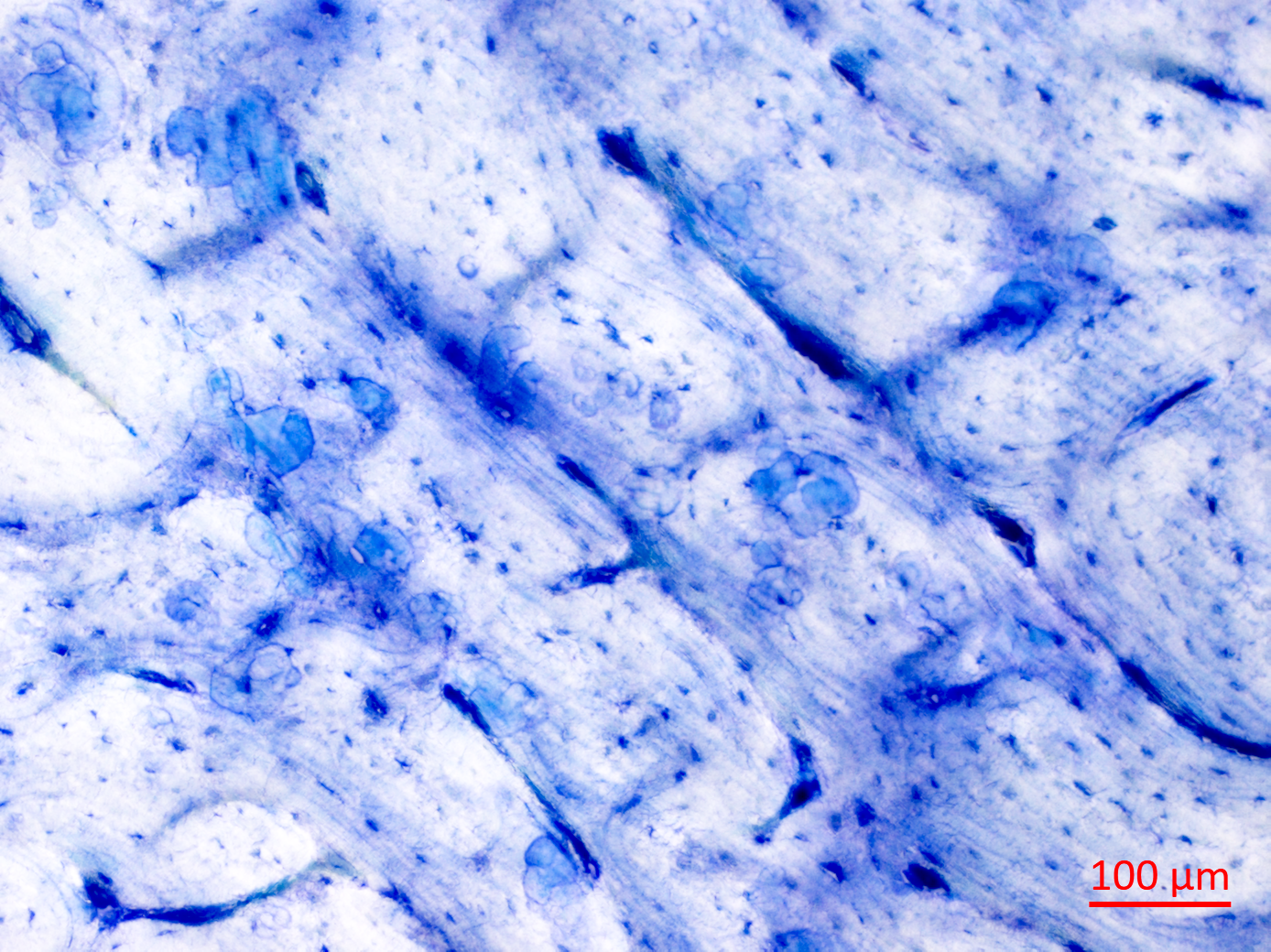

Supplement: Supplementary file 5 — Source data Fig. 3 [file 44319_2024_255_MOESM5_ESM.zip › Figure 3/3H/pit assay_BPTES.tif]

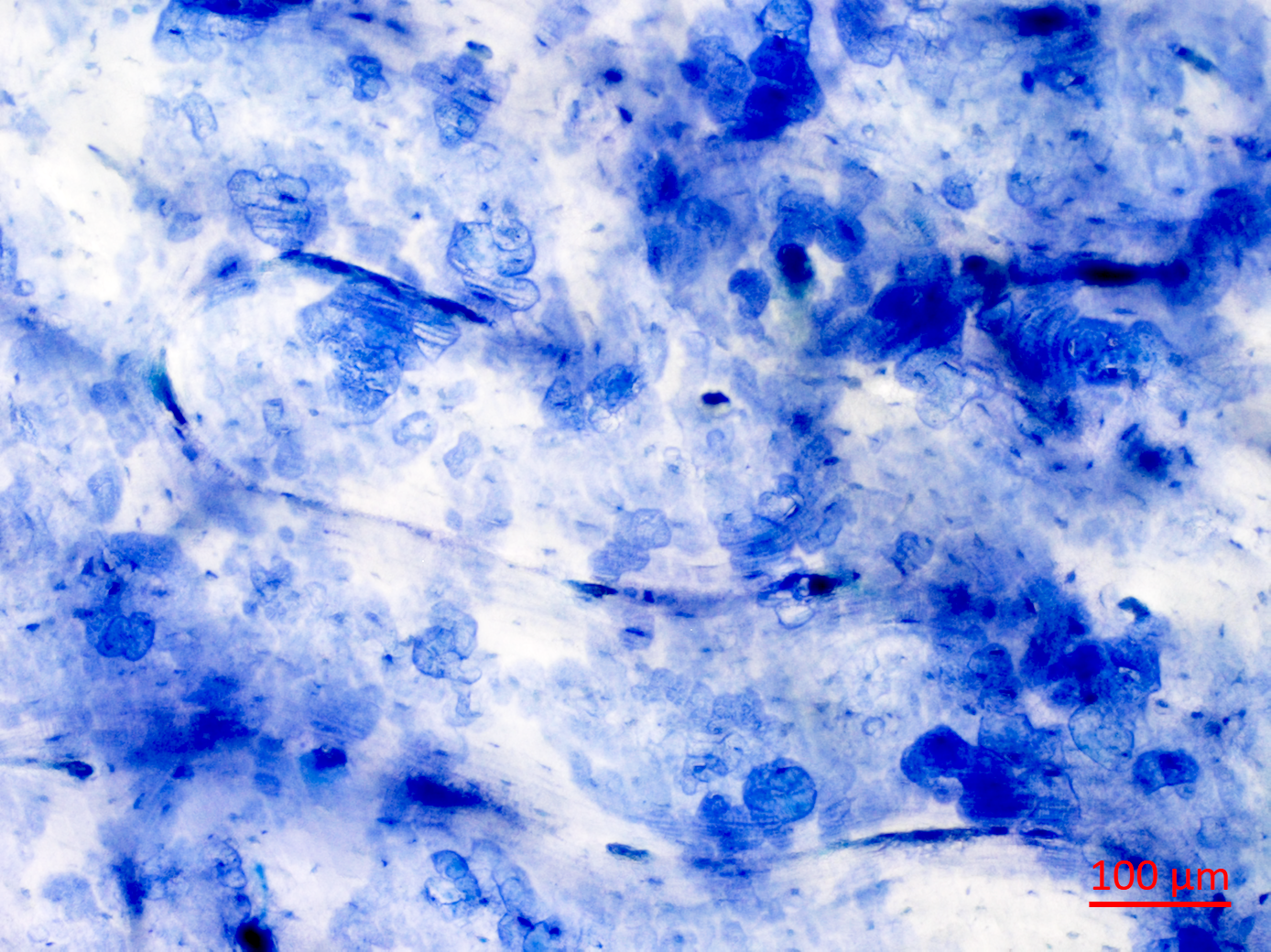

Supplement: Supplementary file 5 — Source data Fig. 3 [file 44319_2024_255_MOESM5_ESM.zip › Figure 3/3H/pit assay_vehicle.tif]

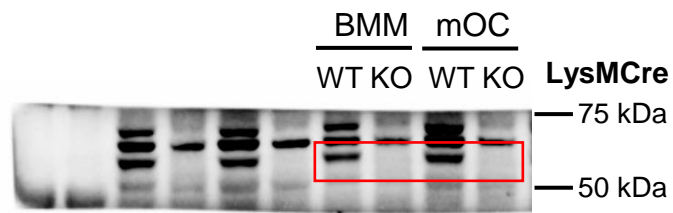

**GLS**

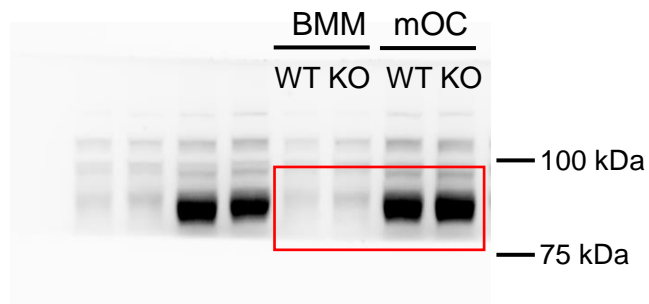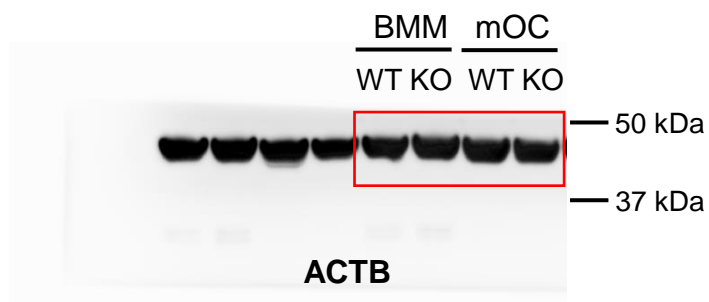

**Full unedited gel for Fig. 4A**

Supplement: Supplementary file 6 — Source data Fig. 4 [file 44319_2024_255_MOESM6_ESM.zip › Figure 4/4A/Full unedited gel for 4A.pdf]

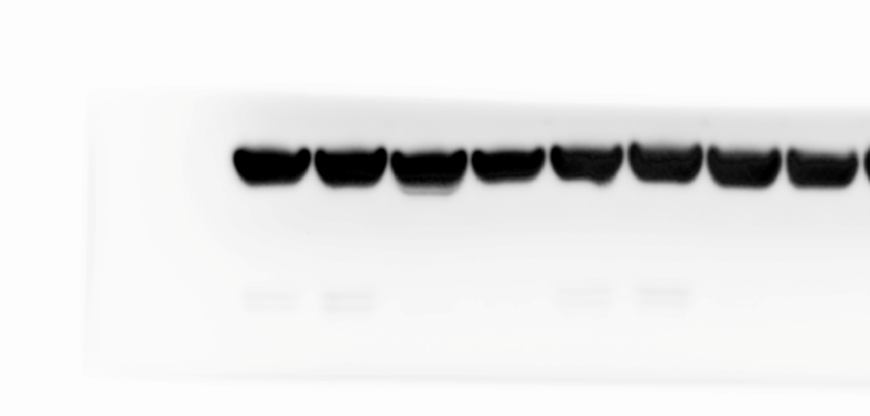

Supplement: Supplementary file 6 — Source data Fig. 4 [file 44319_2024_255_MOESM6_ESM.zip › Figure 4/4A/western blot_ACTB.tif]

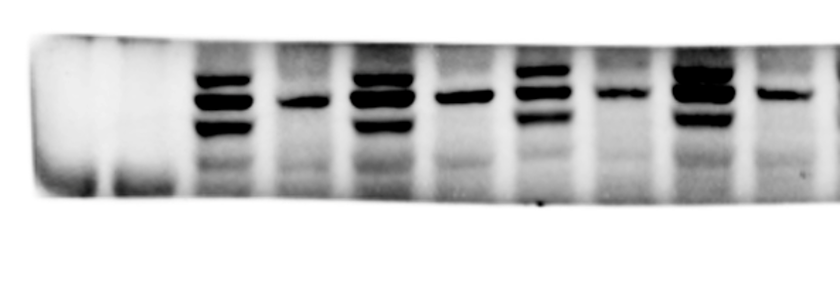

Supplement: Supplementary file 6 — Source data Fig. 4 [file 44319_2024_255_MOESM6_ESM.zip › Figure 4/4A/western blot_GLS.tif]

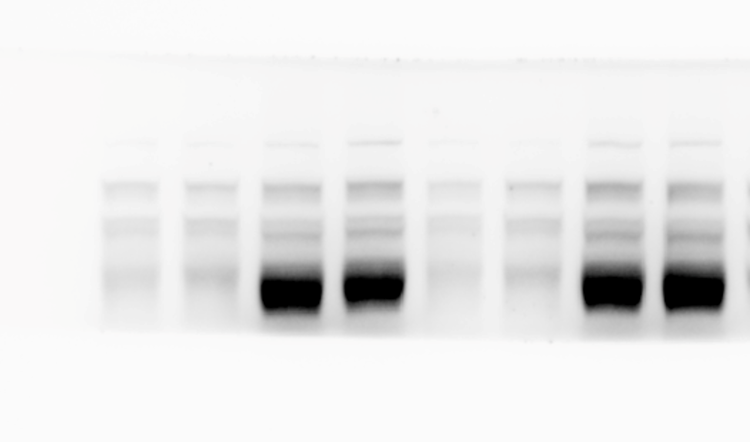

Supplement: Supplementary file 6 — Source data Fig. 4 [file 44319_2024_255_MOESM6_ESM.zip › Figure 4/4A/western blot_NFATc1.tif]

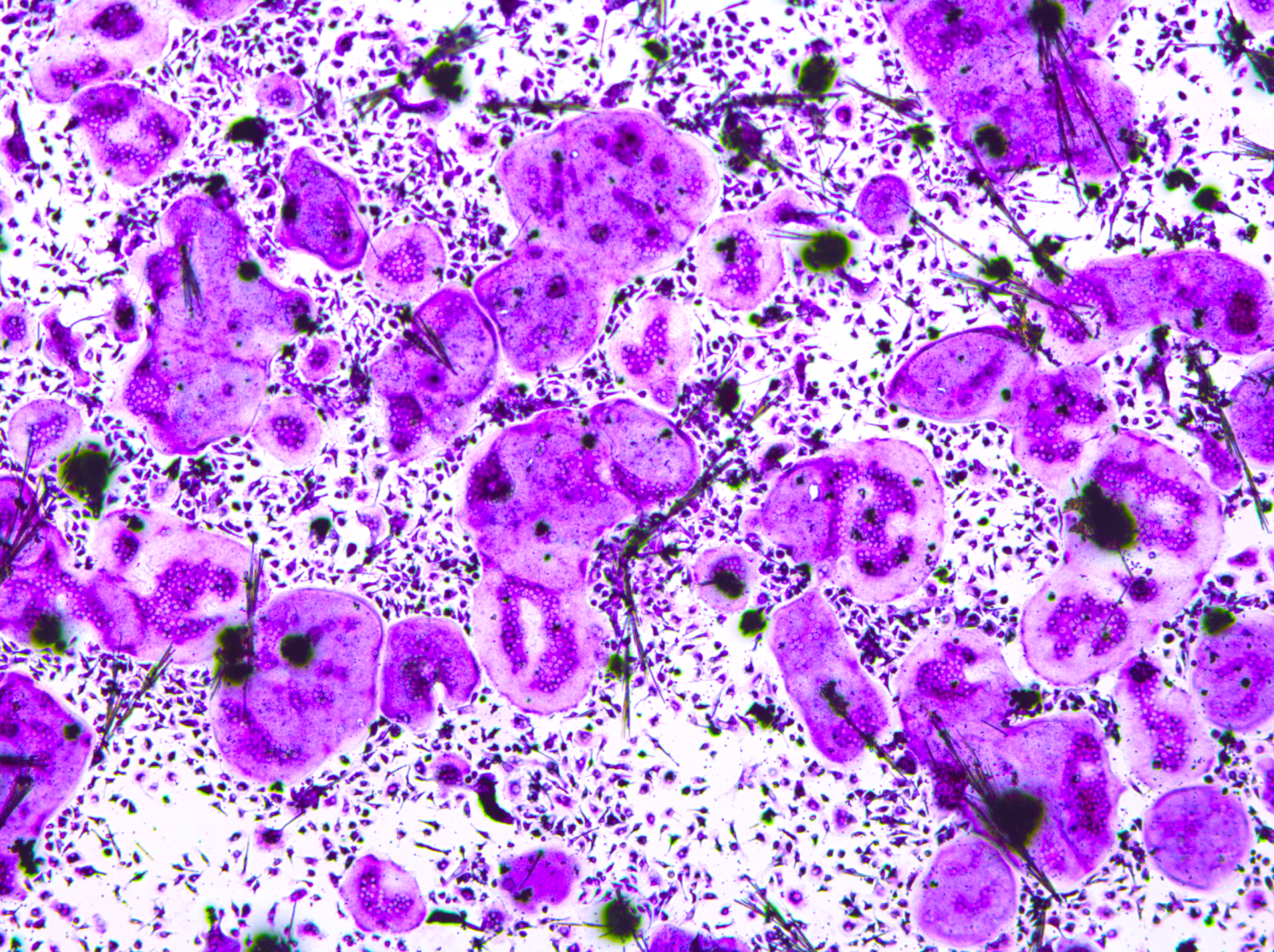

Supplement: Supplementary file 6 — Source data Fig. 4 [file 44319_2024_255_MOESM6_ESM.zip › Figure 4/4B/TRAP_LysM;Glsfl+.tif]

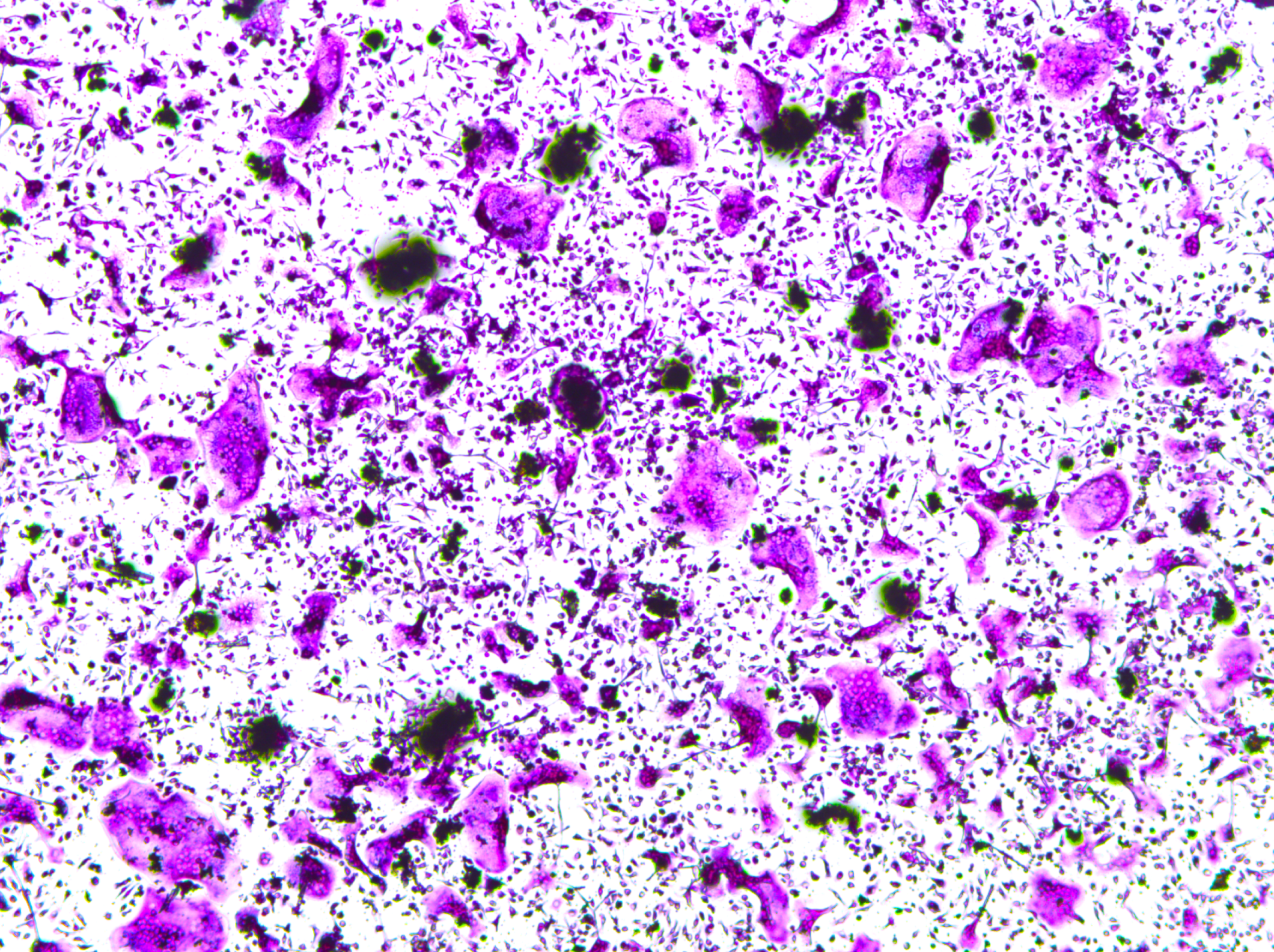

Supplement: Supplementary file 6 — Source data Fig. 4 [file 44319_2024_255_MOESM6_ESM.zip › Figure 4/4B/TRAP_LysM;Glsflfl.tif]

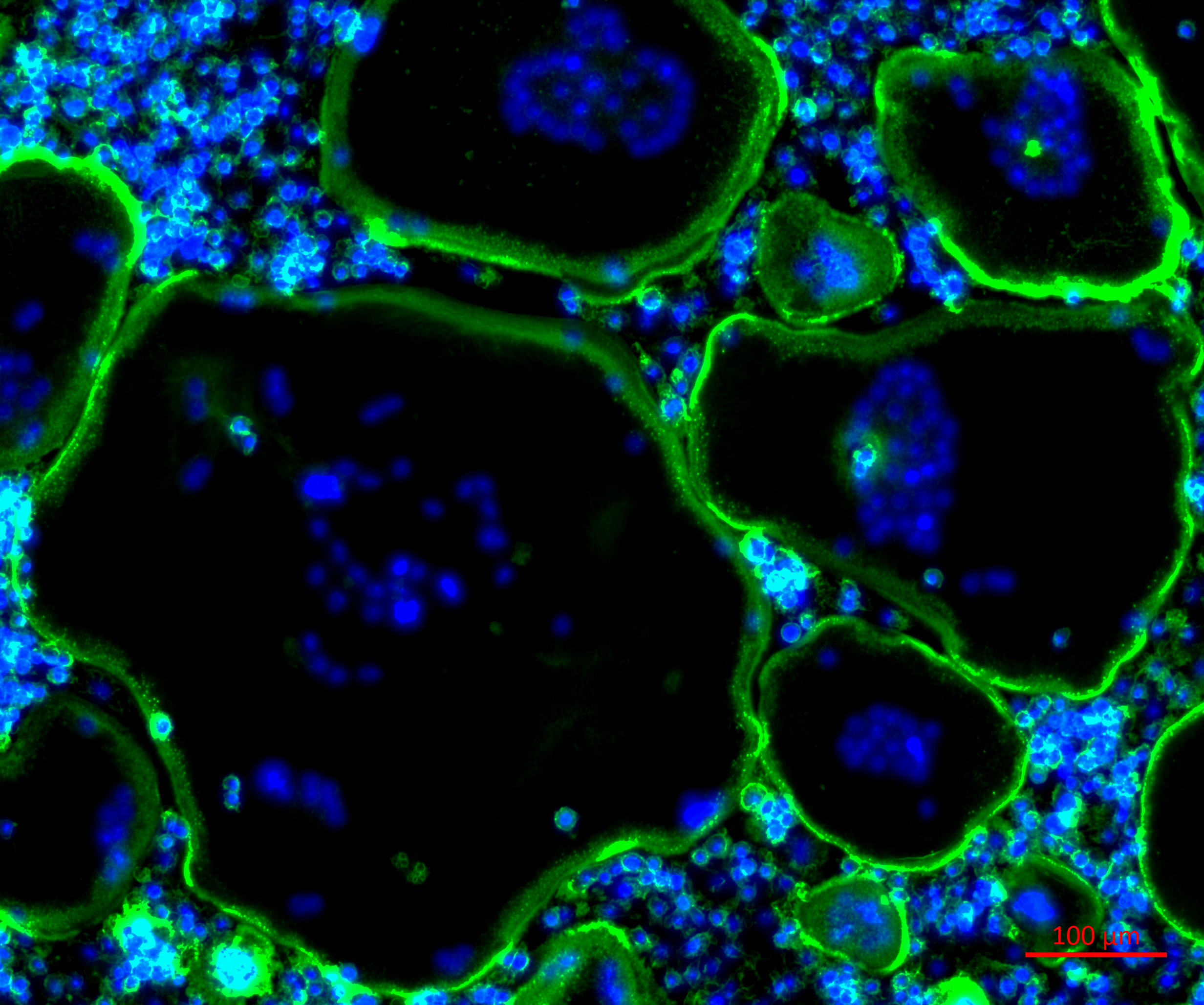

Supplement: Supplementary file 6 — Source data Fig. 4 [file 44319_2024_255_MOESM6_ESM.zip › Figure 4/4E/actin ring_LysM;Glsfl+.tif]

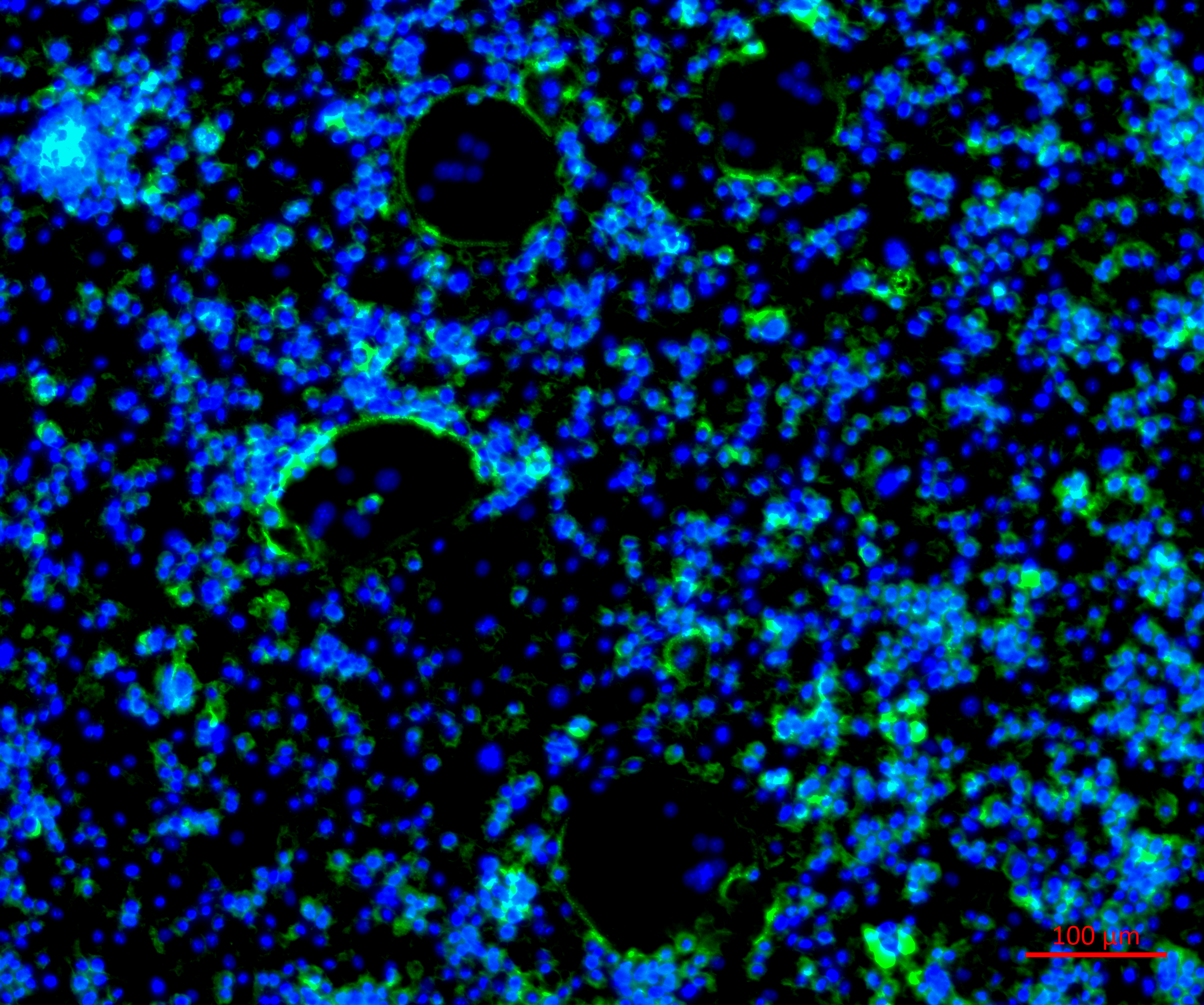

Supplement: Supplementary file 6 — Source data Fig. 4 [file 44319_2024_255_MOESM6_ESM.zip › Figure 4/4E/actin ring_LysM;Glsflfl.tif]

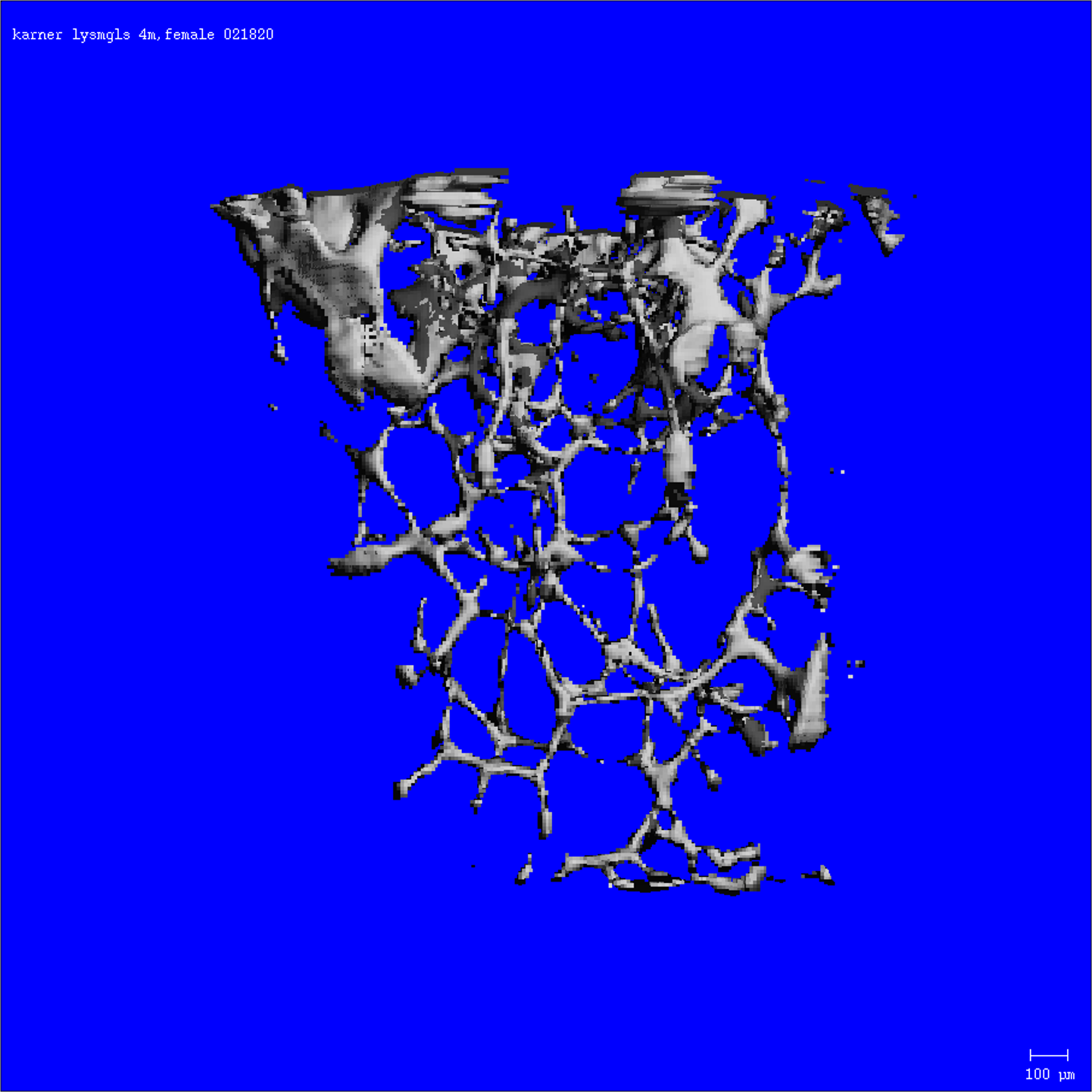

Supplement: Supplementary file 6 — Source data Fig. 4 [file 44319_2024_255_MOESM6_ESM.zip › Figure 4/4G/microCT_LysM;Glsfl+.tif]

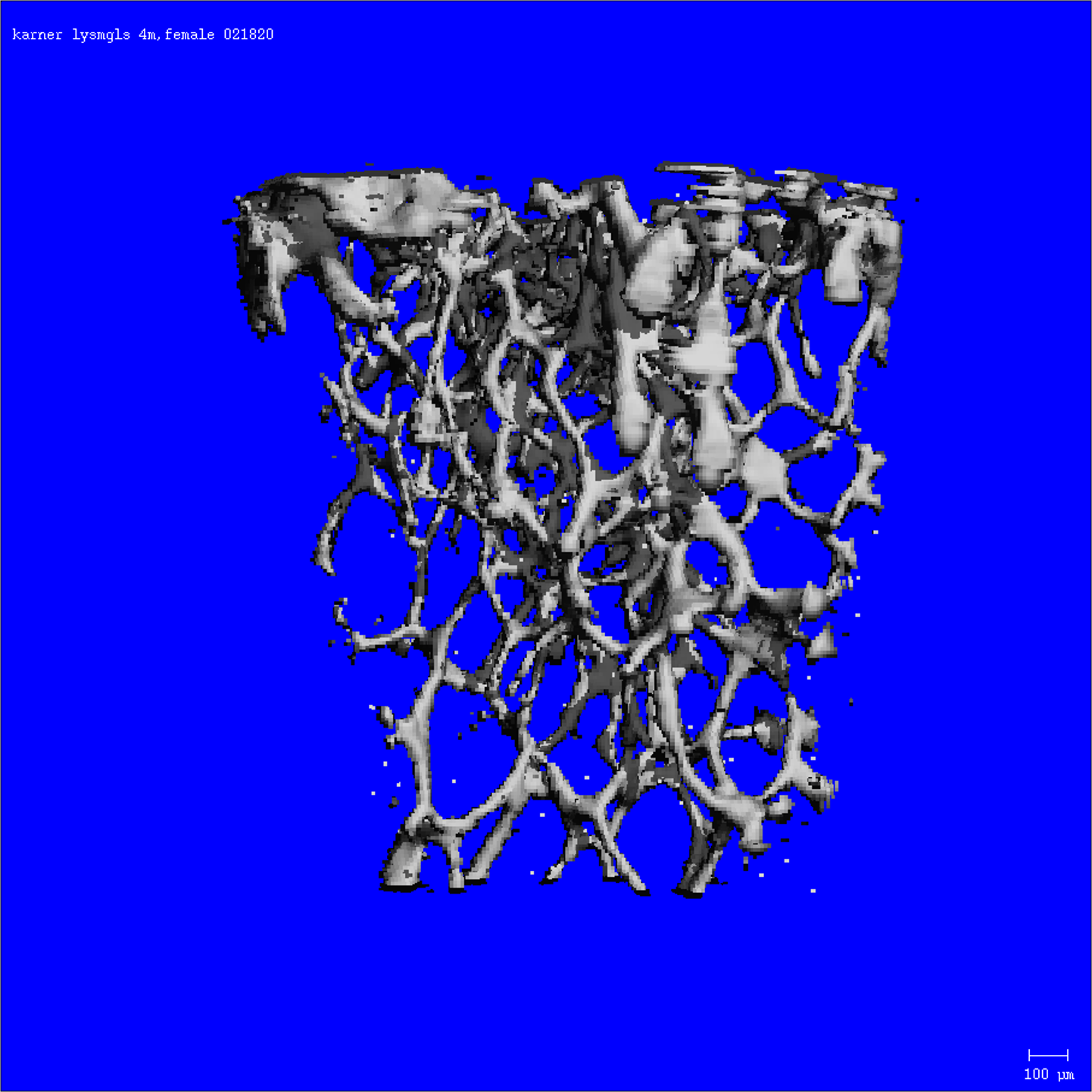

Supplement: Supplementary file 6 — Source data Fig. 4 [file 44319_2024_255_MOESM6_ESM.zip › Figure 4/4G/microCT_LysM;Glsflfl.tif]

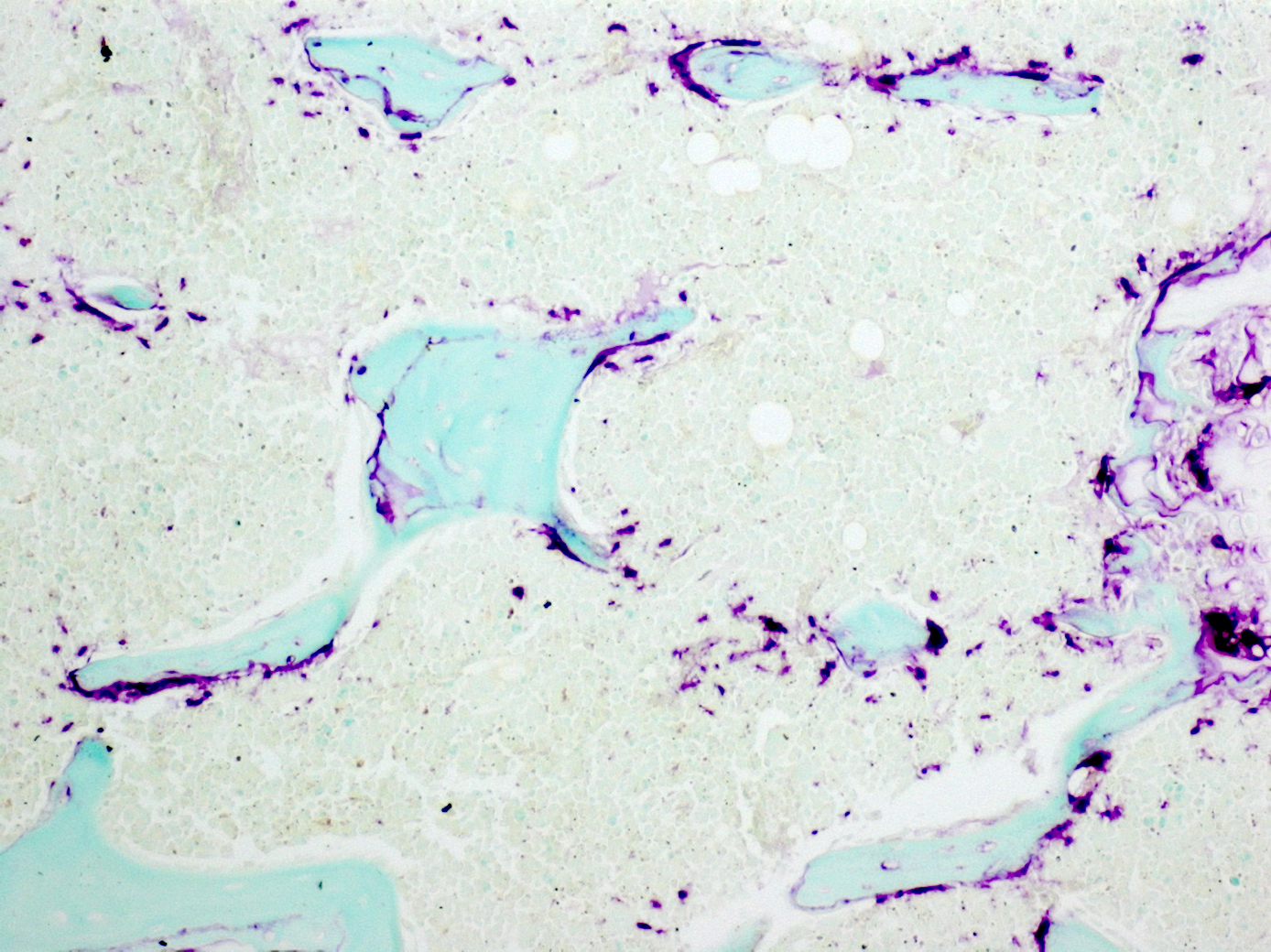

Supplement: Supplementary file 6 — Source data Fig. 4 [file 44319_2024_255_MOESM6_ESM.zip › Figure 4/4I/TRAP_LysM;Glsfl+.tif]

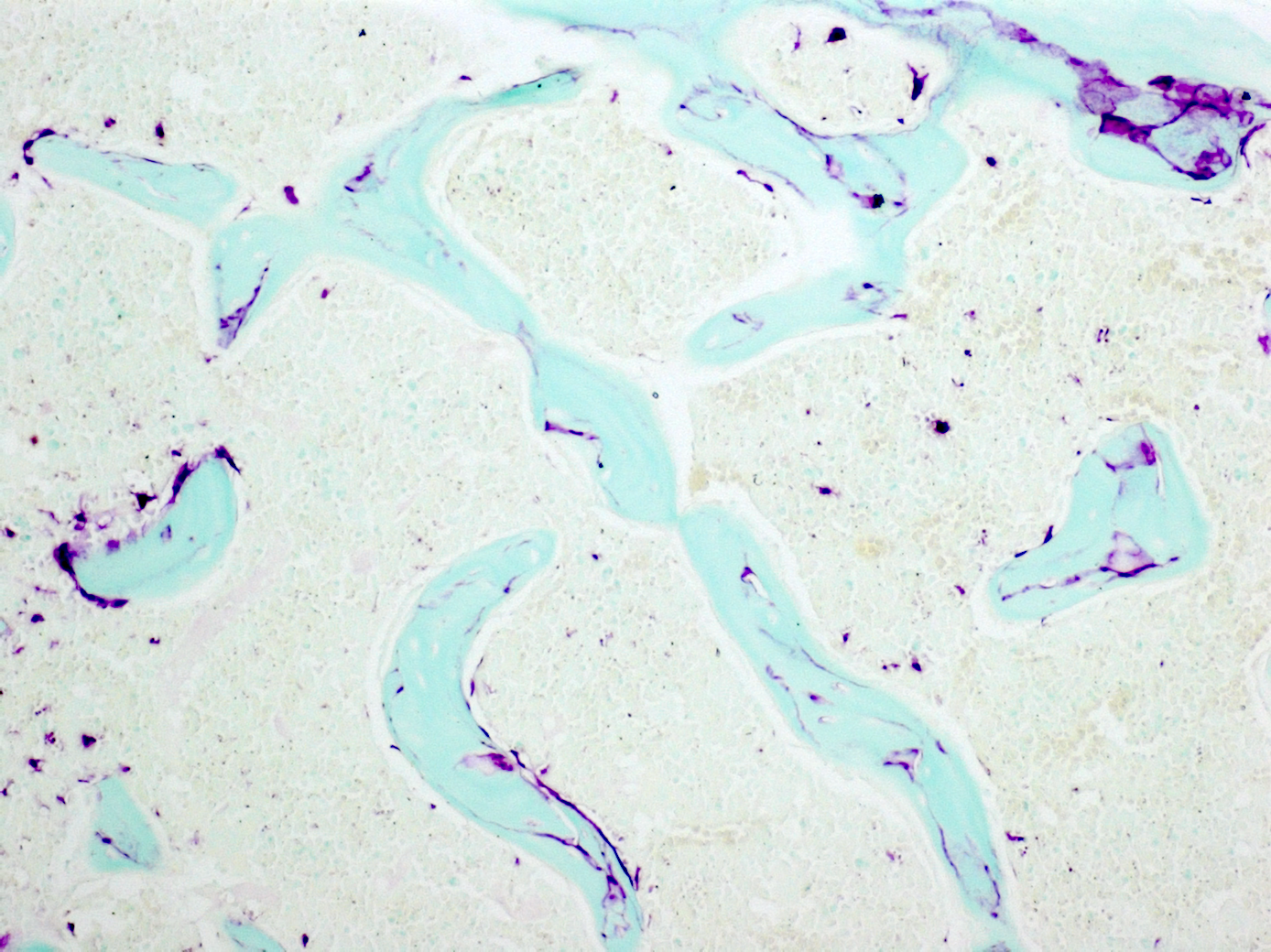

Supplement: Supplementary file 6 — Source data Fig. 4 [file 44319_2024_255_MOESM6_ESM.zip › Figure 4/4I/TRAP_LysM;Glsflfl.tif]

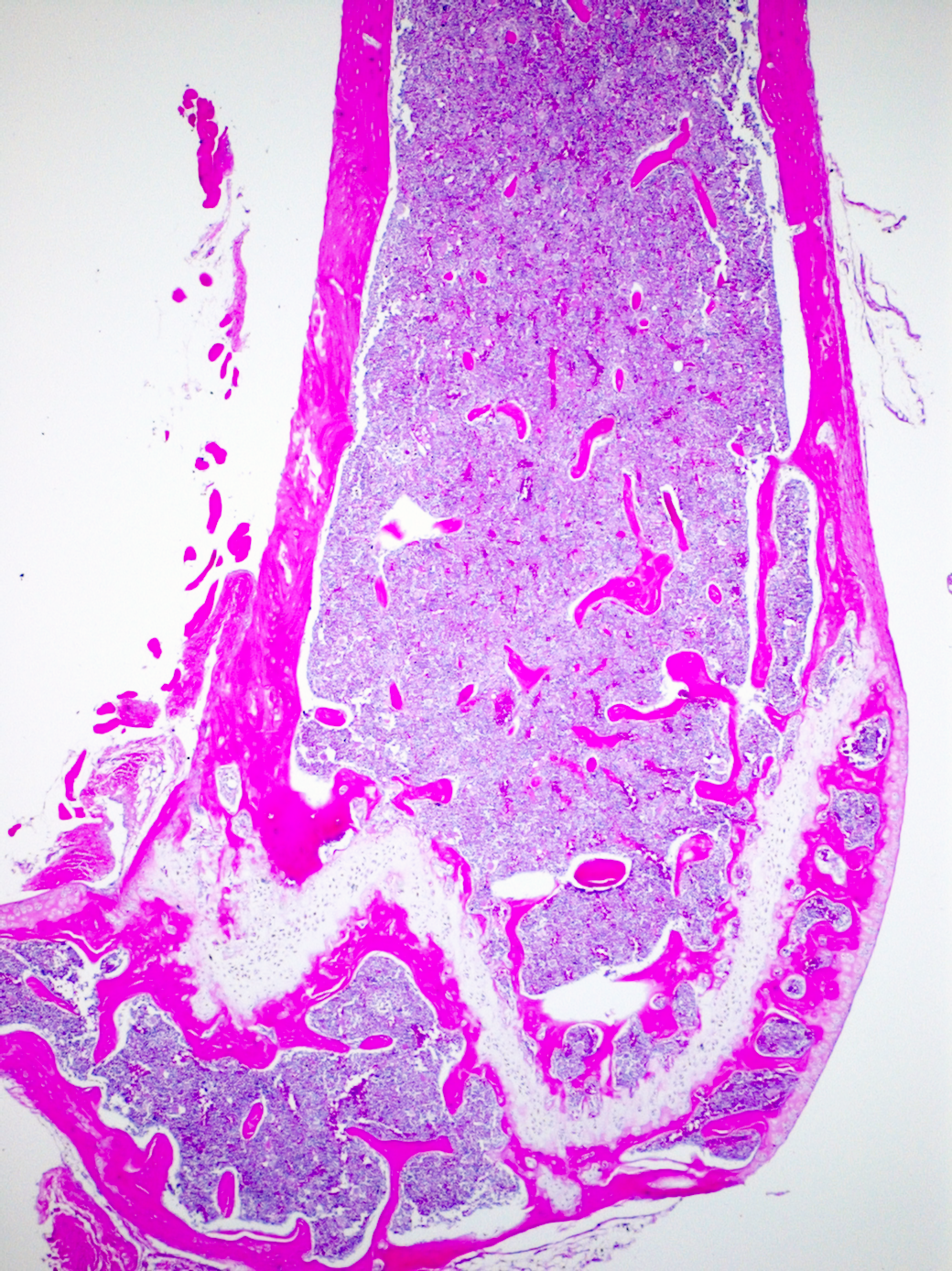

Supplement: Supplementary file 6 — Source data Fig. 4 [file 44319_2024_255_MOESM6_ESM.zip › Figure 4/4M/H&E_LysM;Glsfl+.tif]

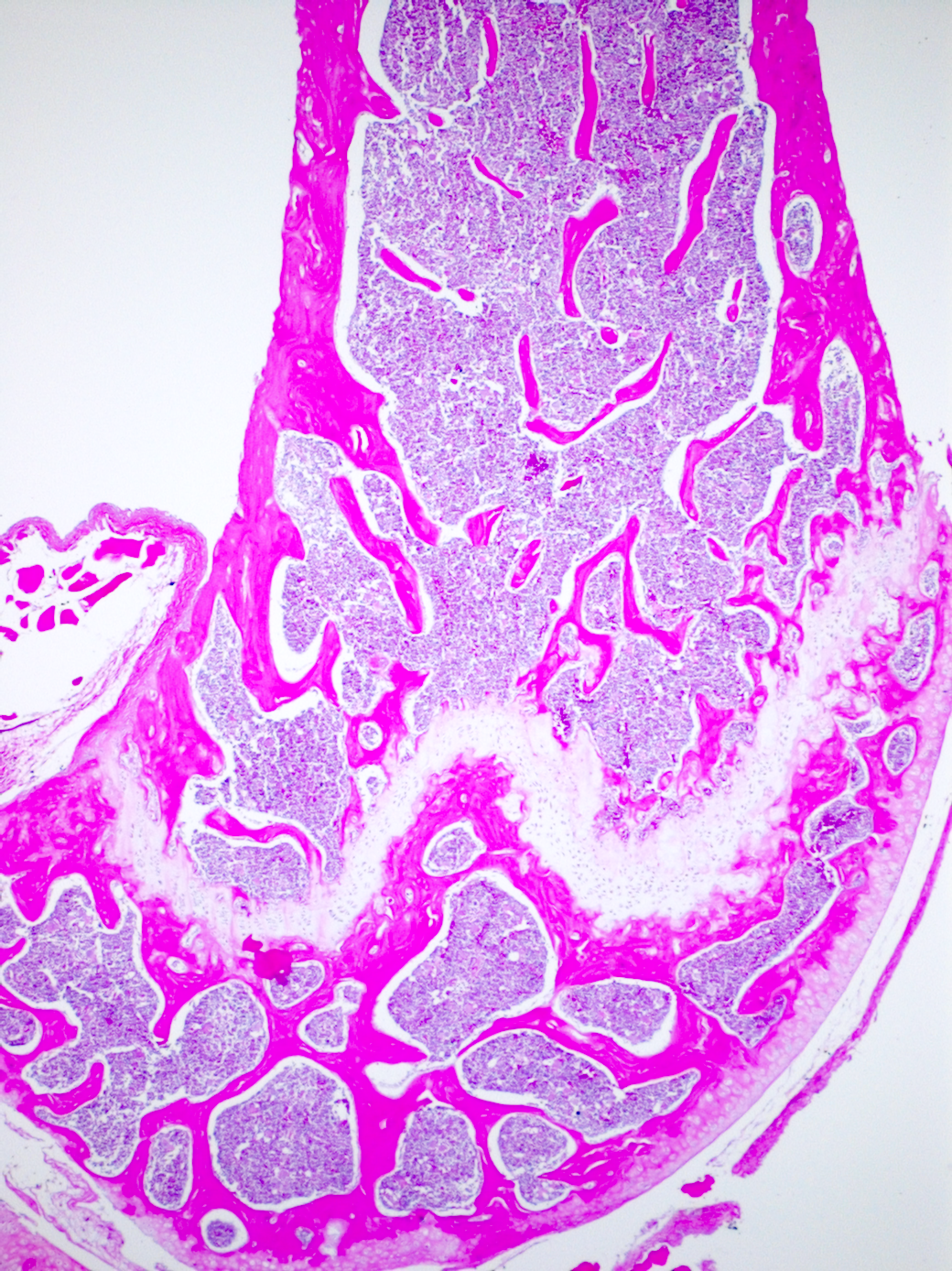

Supplement: Supplementary file 6 — Source data Fig. 4 [file 44319_2024_255_MOESM6_ESM.zip › Figure 4/4M/H&E_LysM;Glsflfl.tif]

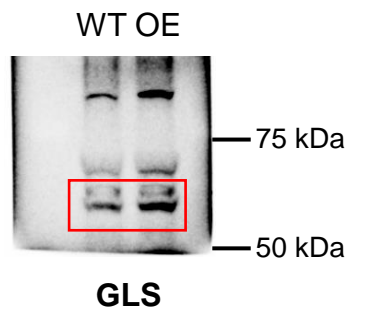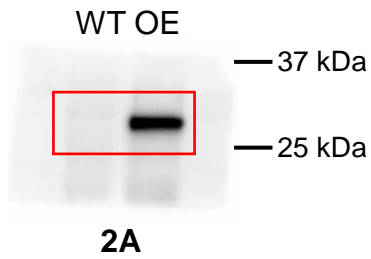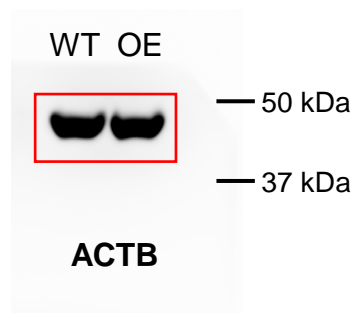

**Full unedited gel for Fig. 5A**

Supplement: Supplementary file 7 — Source data Fig. 5 [file 44319_2024_255_MOESM7_ESM.zip › Figure 5/5A/Full unedited gel for 5A.pdf]

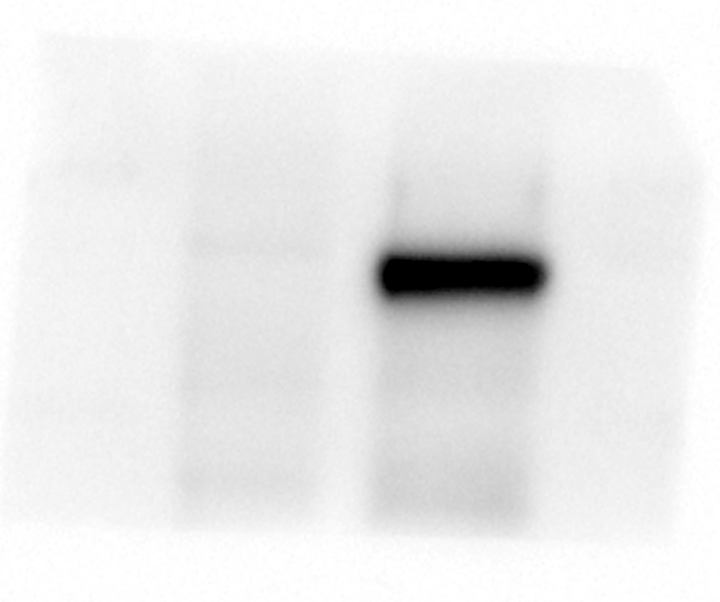

Supplement: Supplementary file 7 — Source data Fig. 5 [file 44319_2024_255_MOESM7_ESM.zip › Figure 5/5A/western blot_2A.tif]

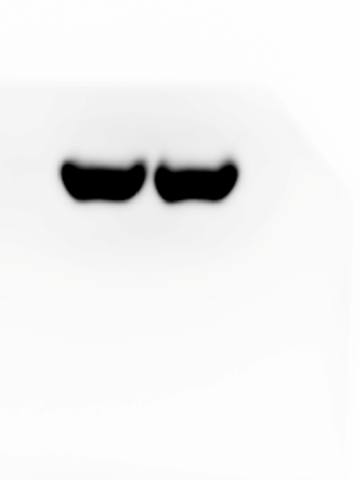

Supplement: Supplementary file 7 — Source data Fig. 5 [file 44319_2024_255_MOESM7_ESM.zip › Figure 5/5A/western blot_ACTB.tif]

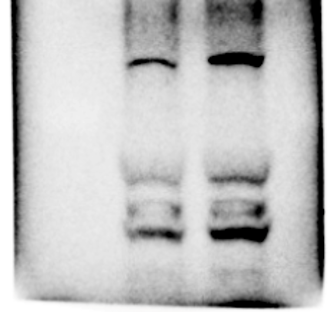

Supplement: Supplementary file 7 — Source data Fig. 5 [file 44319_2024_255_MOESM7_ESM.zip › Figure 5/5A/western blot_GLS.tif]

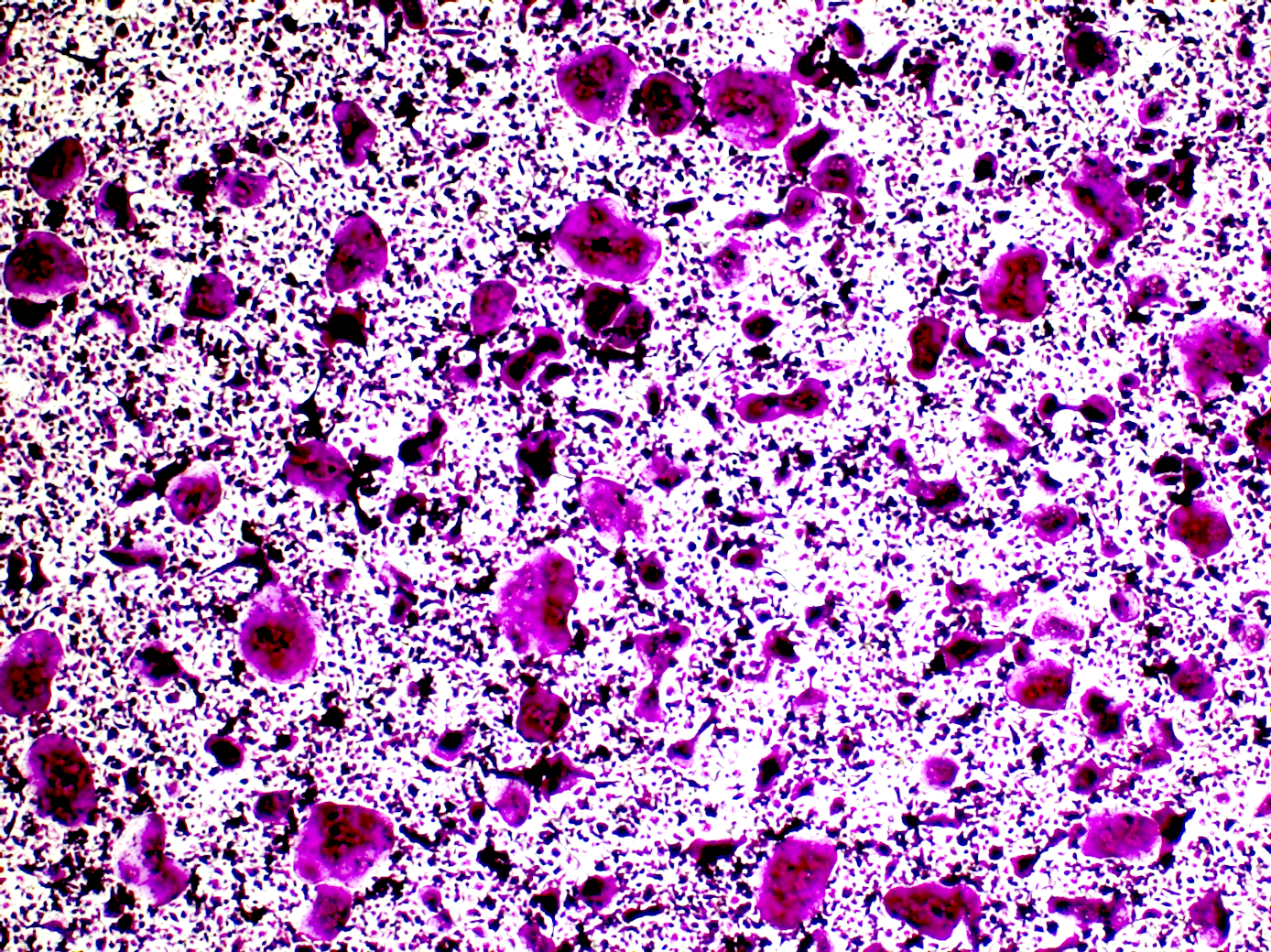

Supplement: Supplementary file 7 — Source data Fig. 5 [file 44319_2024_255_MOESM7_ESM.zip › Figure 5/5B/TRAP_LysM;GlsDOXON-Day 2.tif]

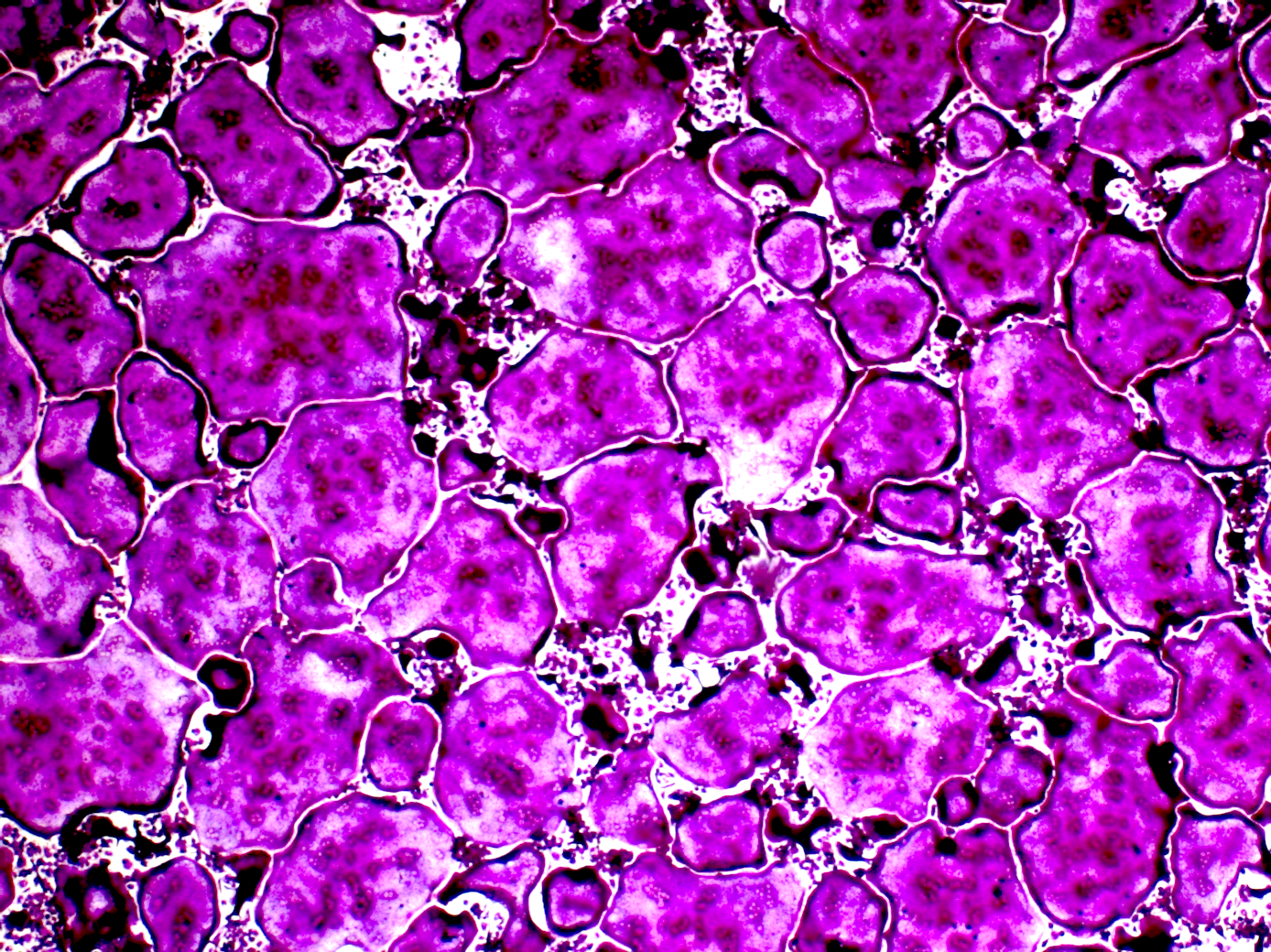

Supplement: Supplementary file 7 — Source data Fig. 5 [file 44319_2024_255_MOESM7_ESM.zip › Figure 5/5B/TRAP_LysM;GlsDOXON-Day 4.tif]

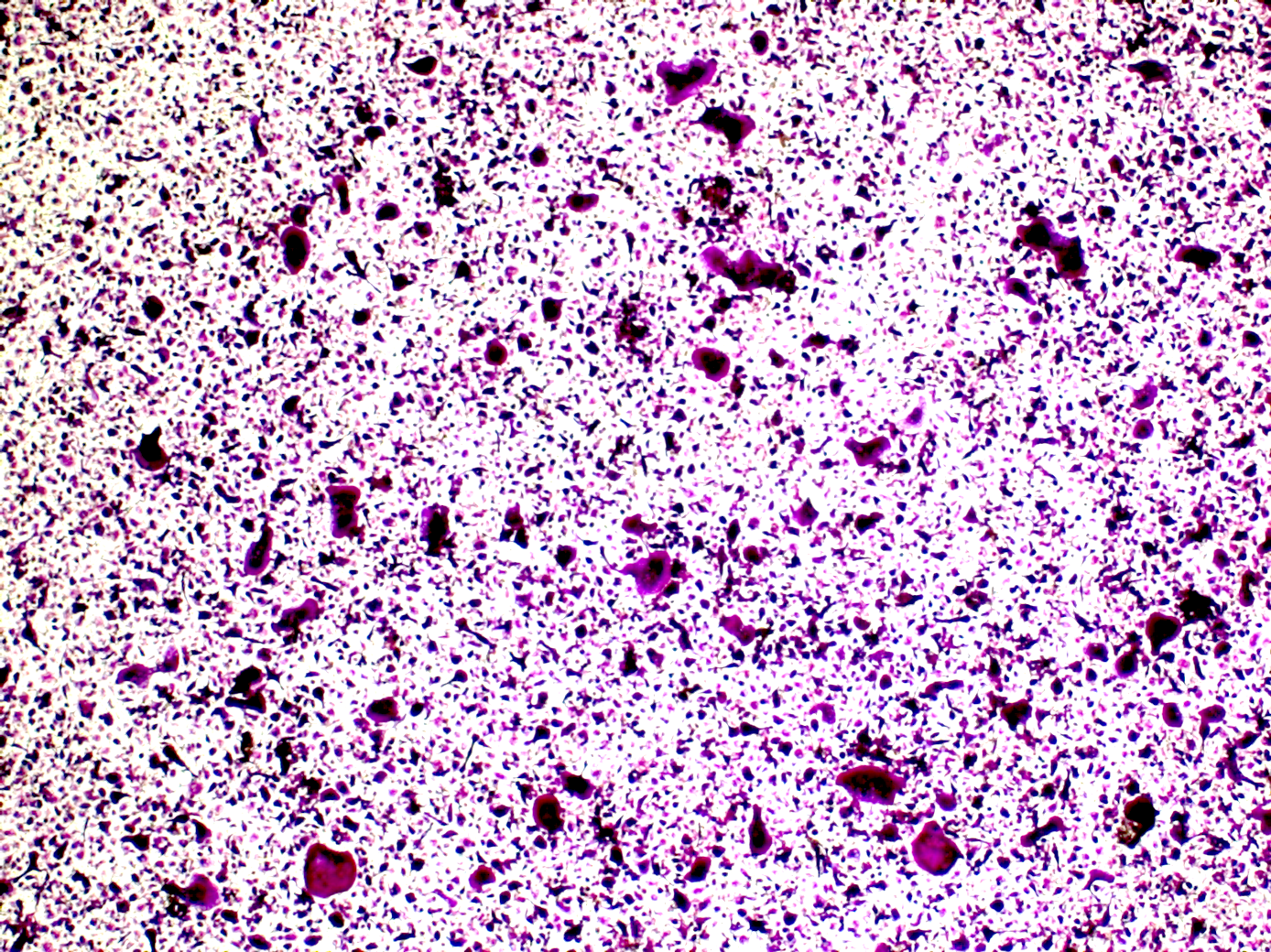

Supplement: Supplementary file 7 — Source data Fig. 5 [file 44319_2024_255_MOESM7_ESM.zip › Figure 5/5B/TRAP_WT-Day 2.tif]

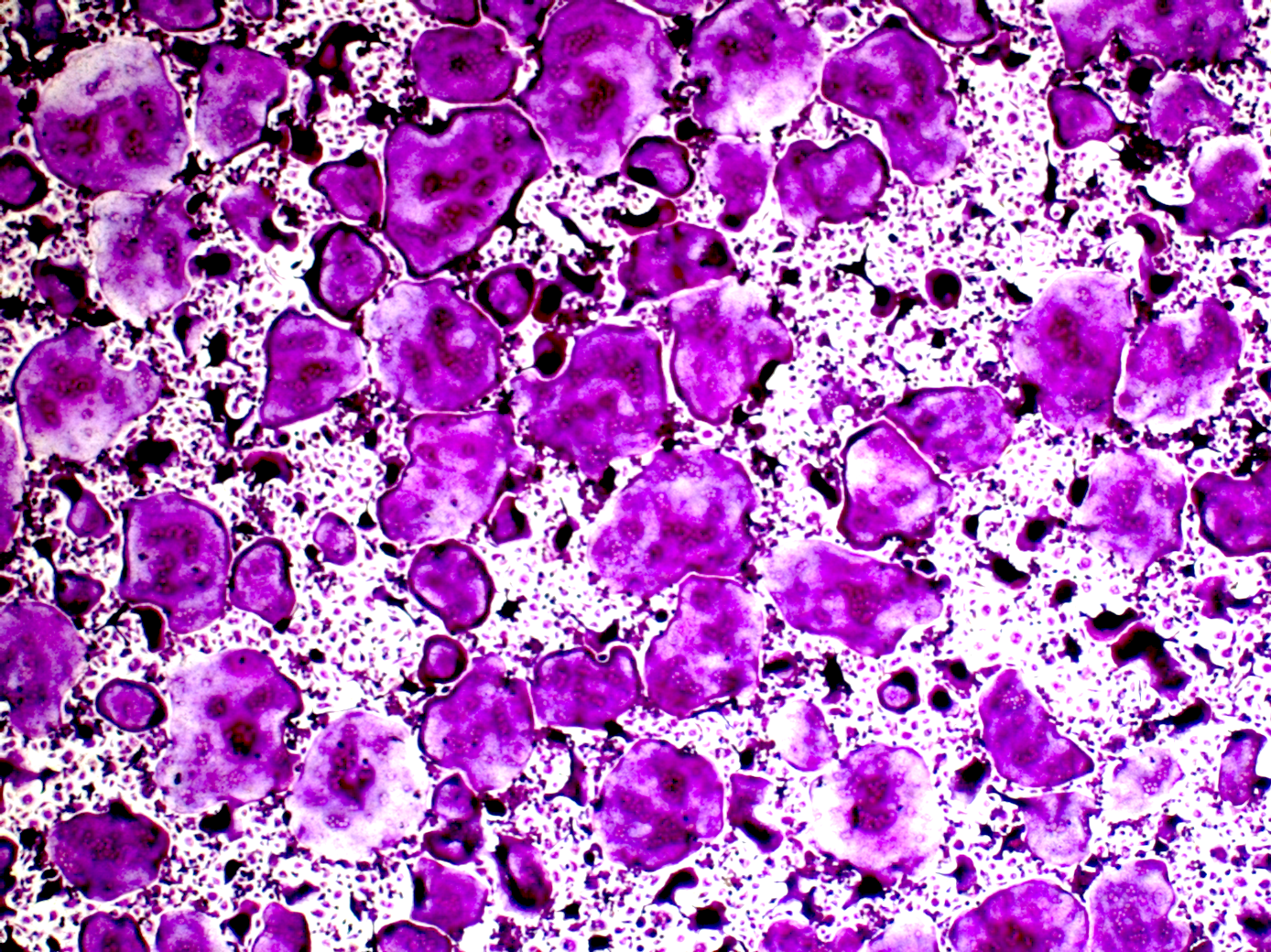

Supplement: Supplementary file 7 — Source data Fig. 5 [file 44319_2024_255_MOESM7_ESM.zip › Figure 5/5B/TRAP_WT-Day 4.tif]

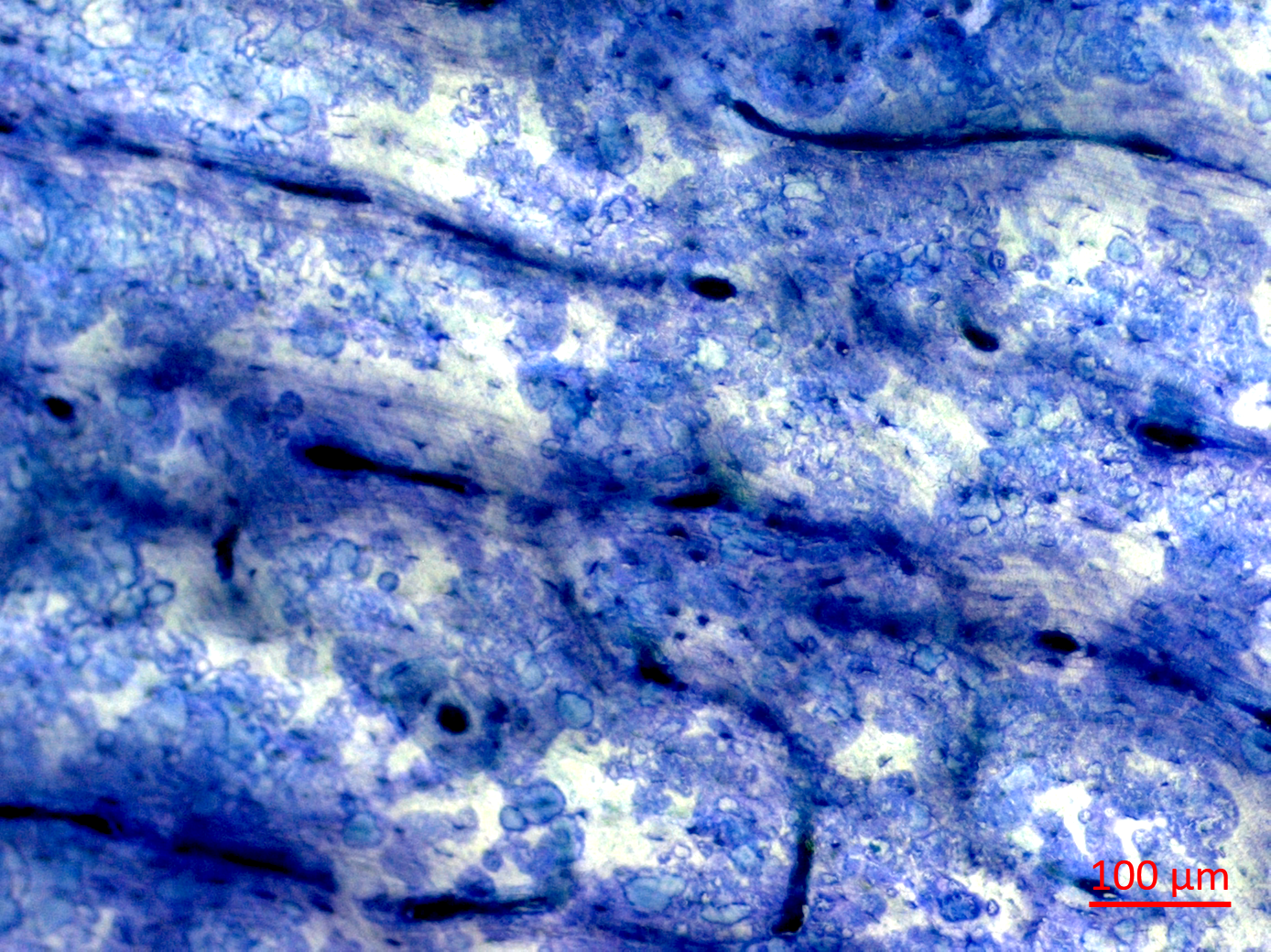

Supplement: Supplementary file 7 — Source data Fig. 5 [file 44319_2024_255_MOESM7_ESM.zip › Figure 5/5E/Pit assay_LysM;GlsDOXON.tif]

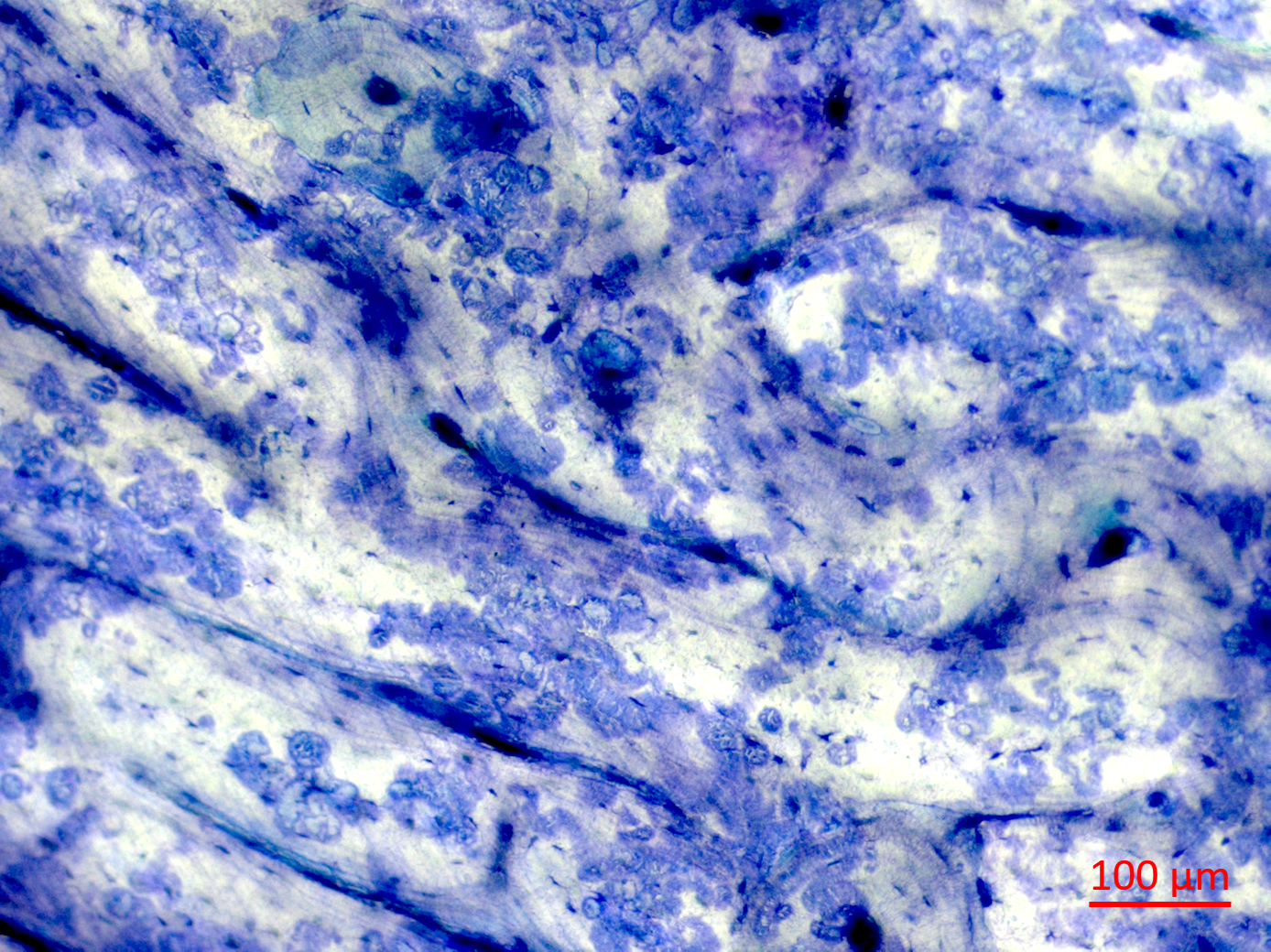

Supplement: Supplementary file 7 — Source data Fig. 5 [file 44319_2024_255_MOESM7_ESM.zip › Figure 5/5E/Pit assay_WT.tif]

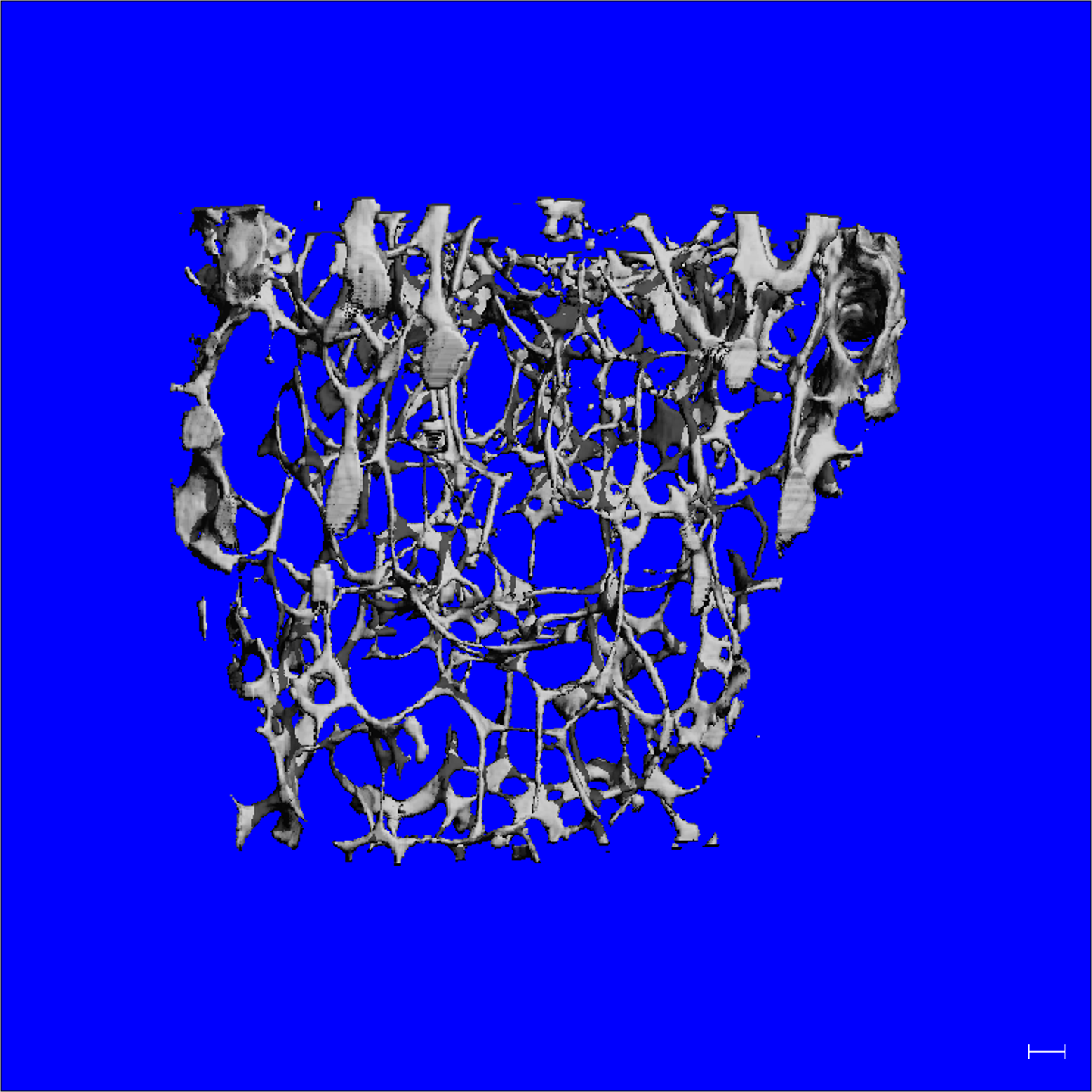

Supplement: Supplementary file 7 — Source data Fig. 5 [file 44319_2024_255_MOESM7_ESM.zip › Figure 5/5G/microCT_LysM;GlsDOXON.tif]

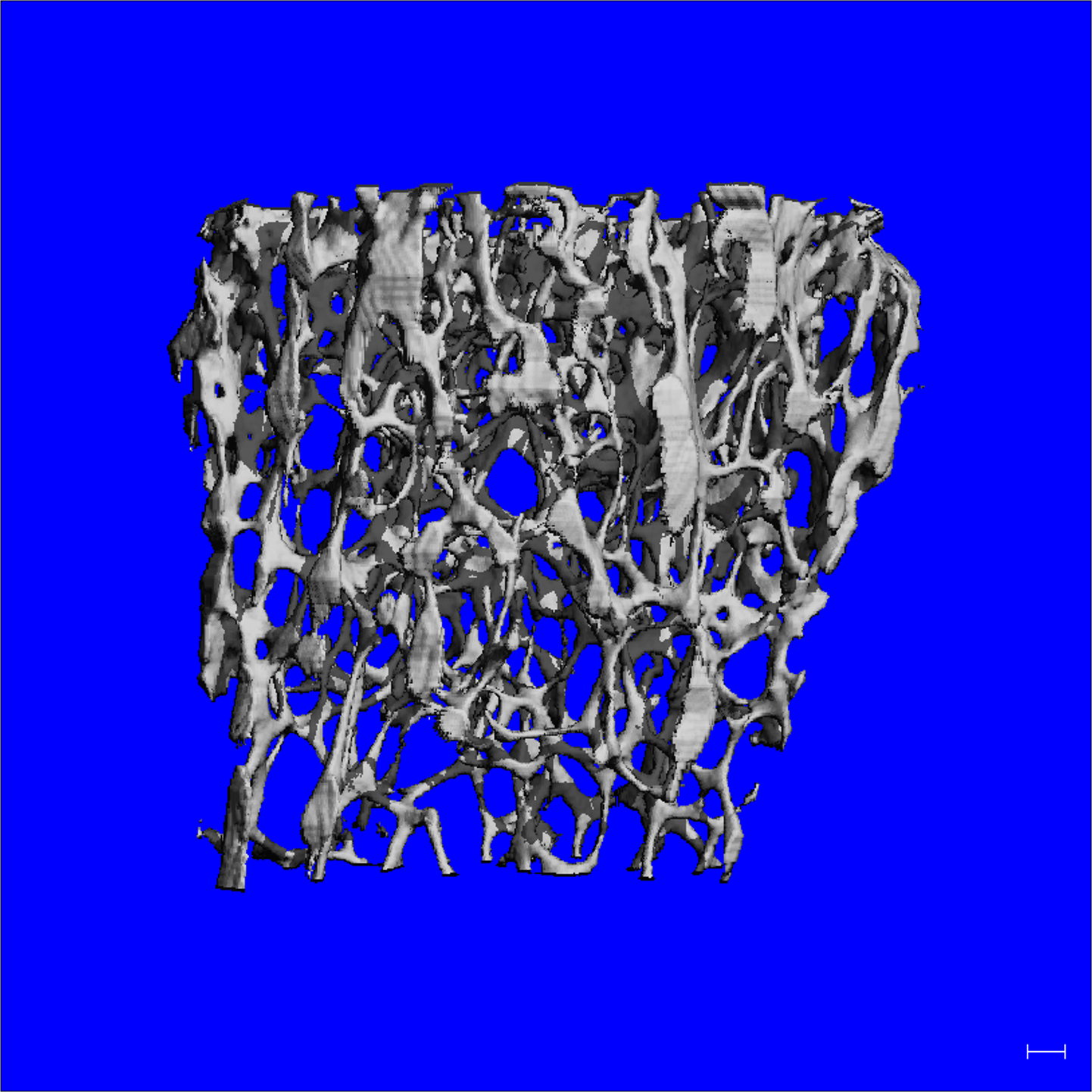

Supplement: Supplementary file 7 — Source data Fig. 5 [file 44319_2024_255_MOESM7_ESM.zip › Figure 5/5G/microCT_WT.tif]

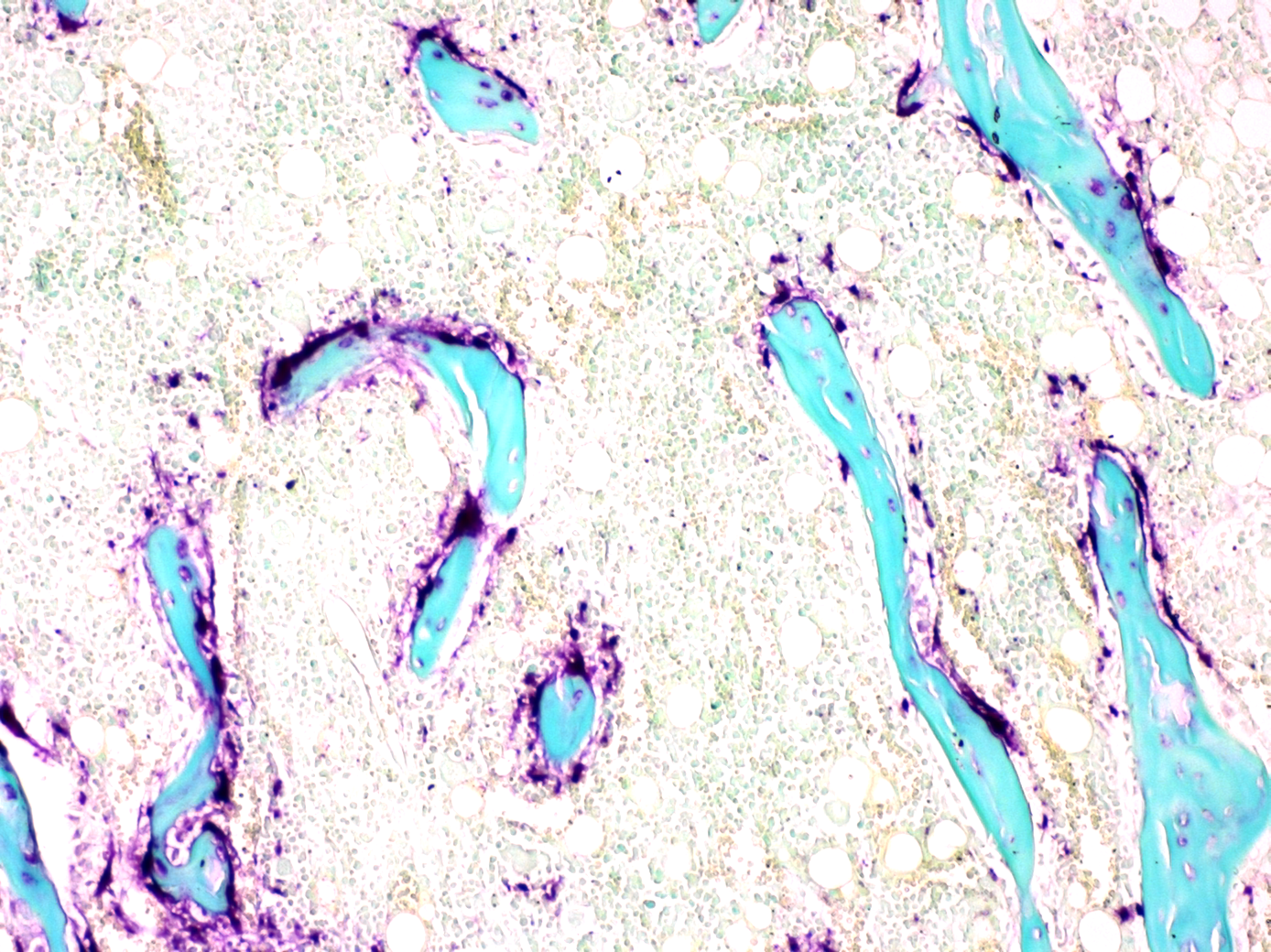

Supplement: Supplementary file 7 — Source data Fig. 5 [file 44319_2024_255_MOESM7_ESM.zip › Figure 5/5K/TRAP_LysM;GlsDOXON.tif]

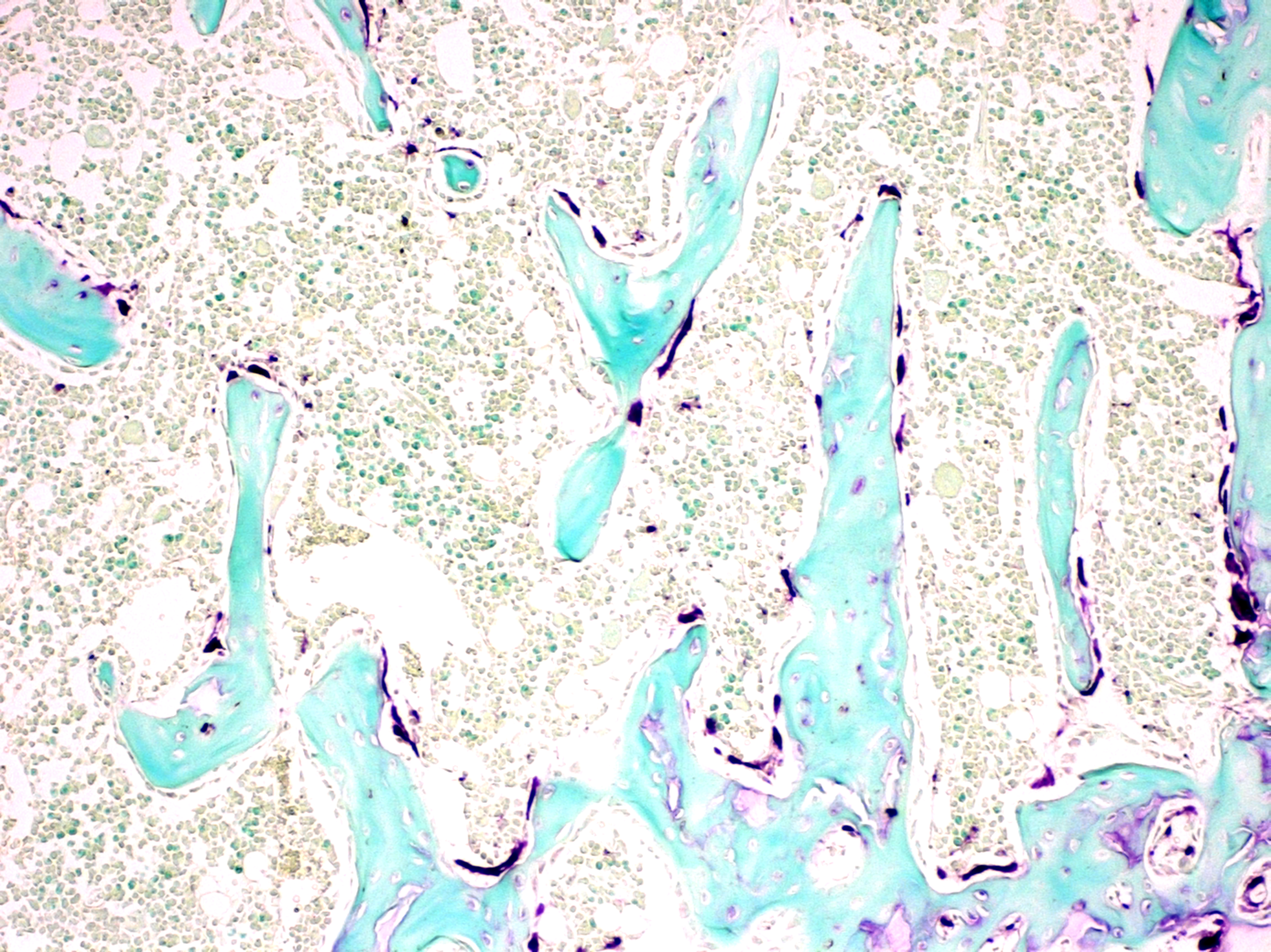

Supplement: Supplementary file 7 — Source data Fig. 5 [file 44319_2024_255_MOESM7_ESM.zip › Figure 5/5K/TRAP_WT.tif]

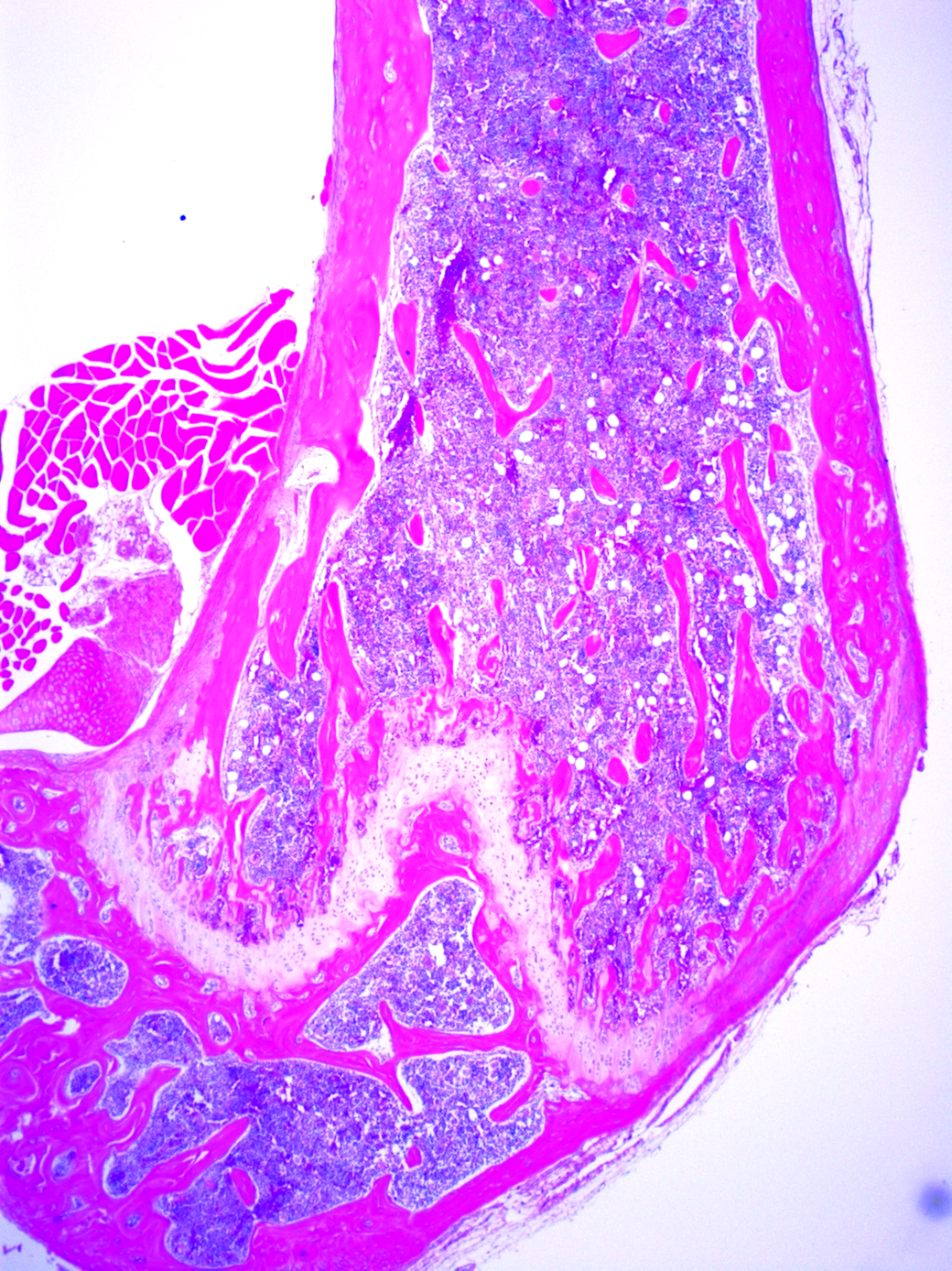

Supplement: Supplementary file 7 — Source data Fig. 5 [file 44319_2024_255_MOESM7_ESM.zip › Figure 5/5M/H&E_LysM;GlsDOXON.tif]

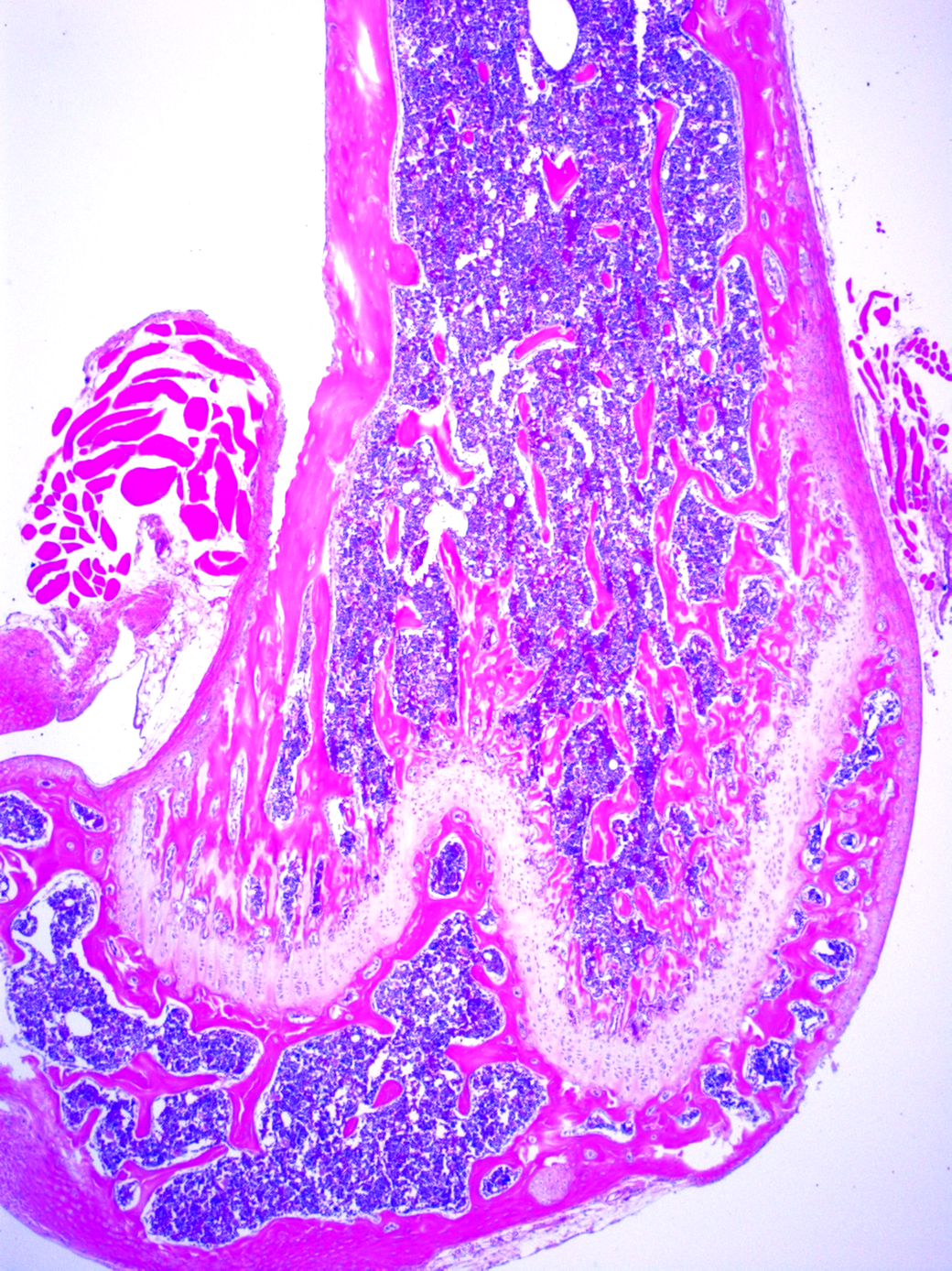

Supplement: Supplementary file 7 — Source data Fig. 5 [file 44319_2024_255_MOESM7_ESM.zip › Figure 5/5M/H&E_WT.tif]

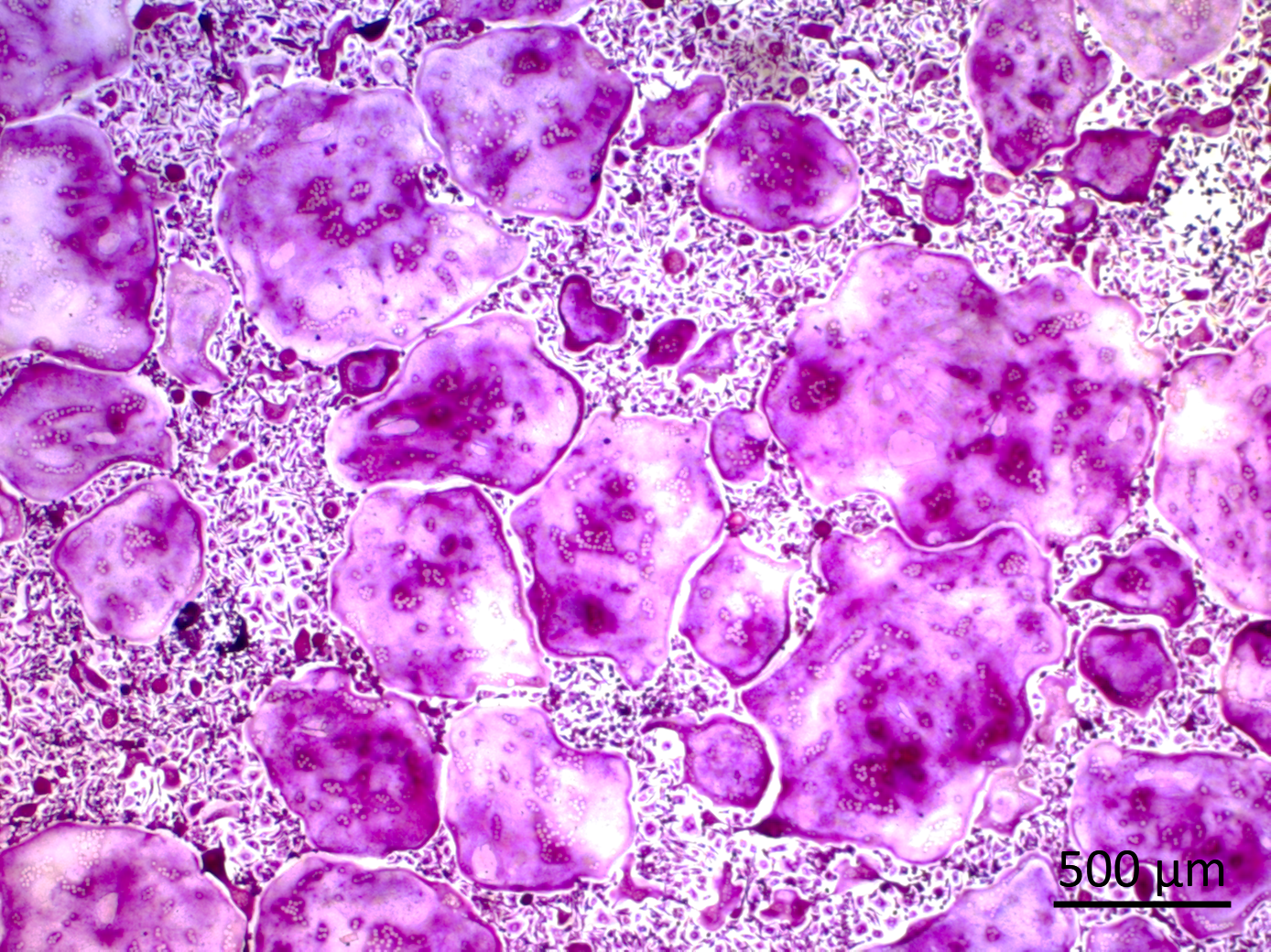

Supplement: Supplementary file 8 — Source data Fig. 6 [file 44319_2024_255_MOESM8_ESM.zip › Figure 6/6A/TRAP_Control.tif]

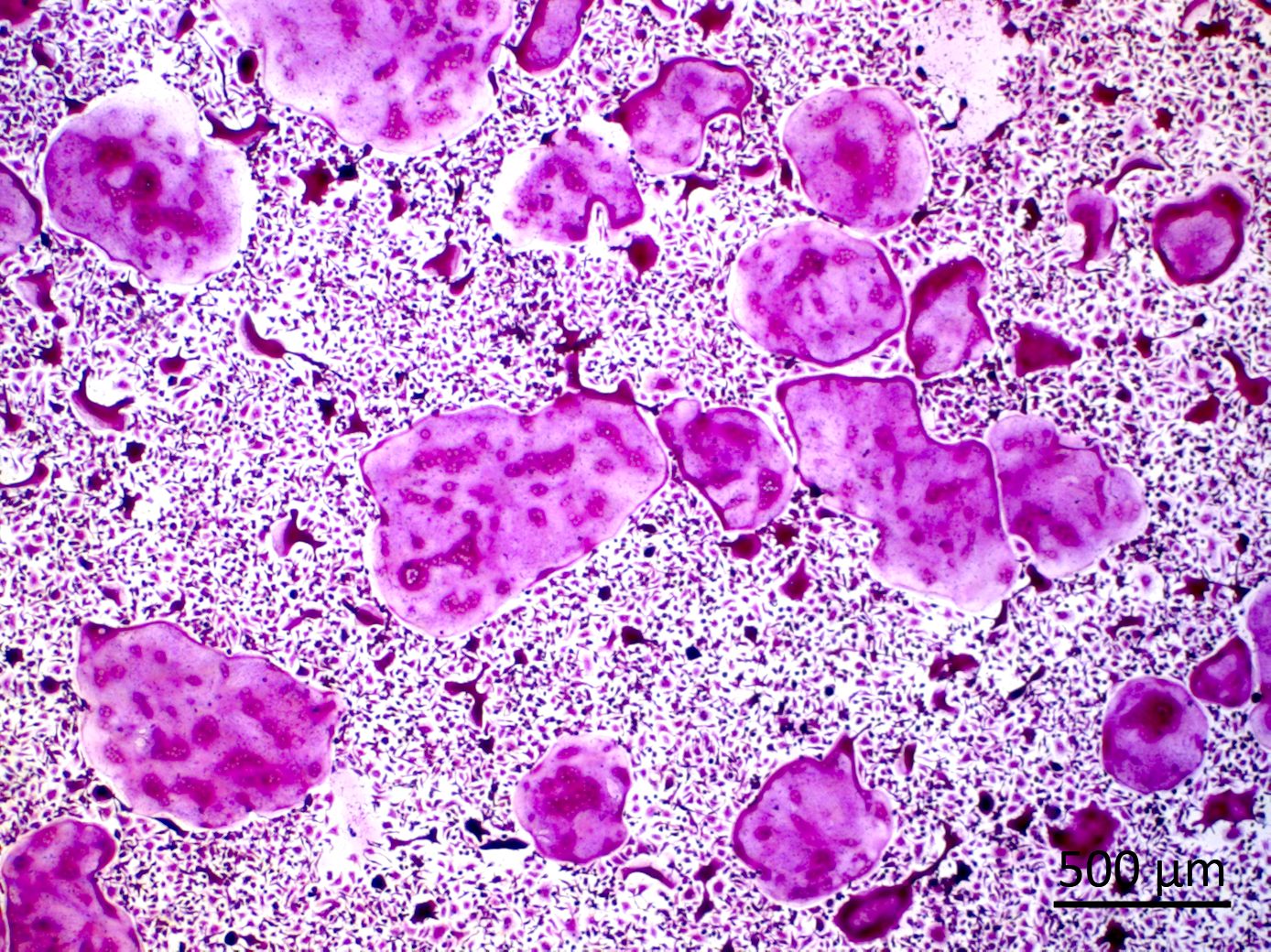

Supplement: Supplementary file 8 — Source data Fig. 6 [file 44319_2024_255_MOESM8_ESM.zip › Figure 6/6A/TRAP_NEAAs&Nucleotides.tif]

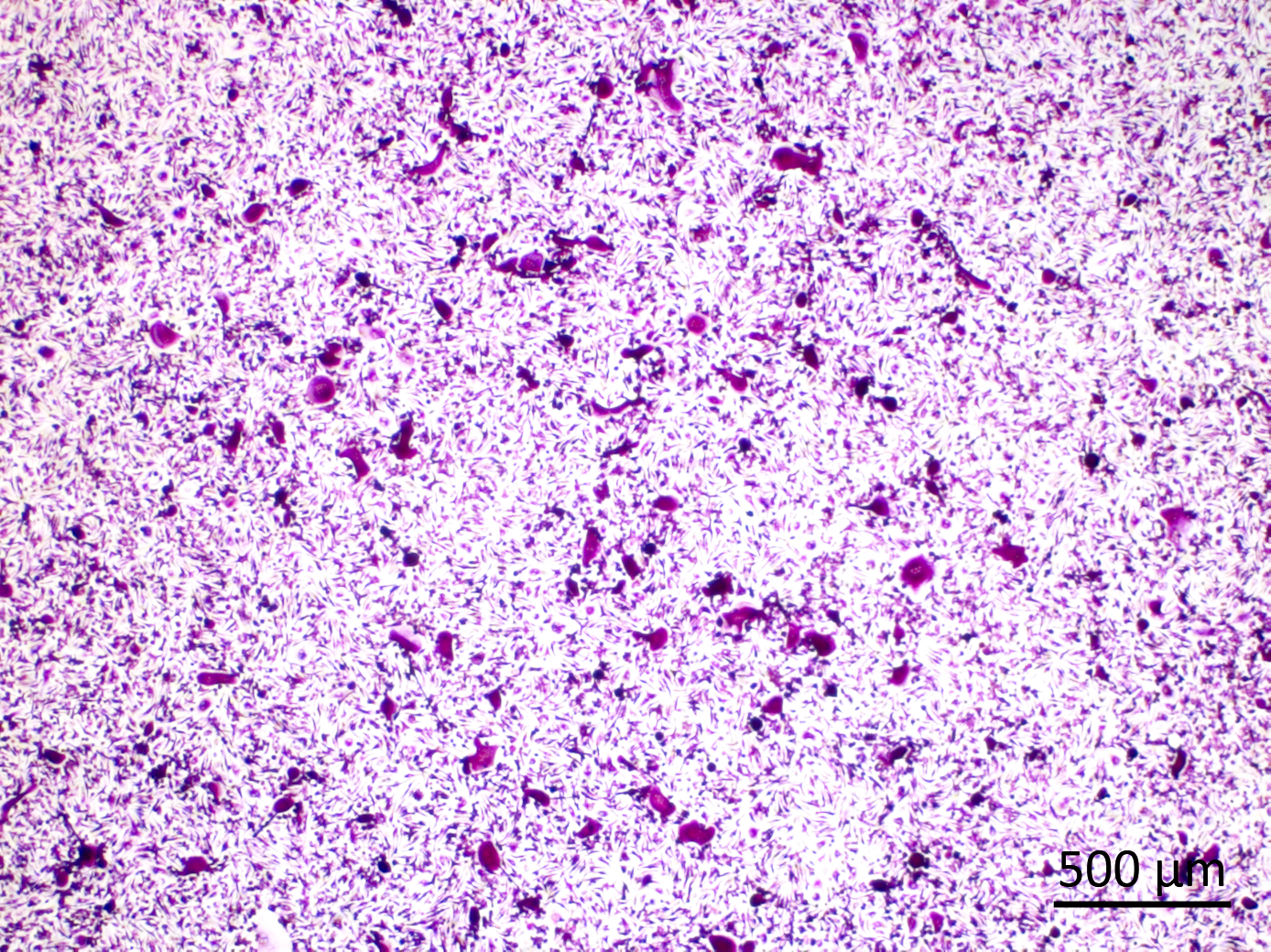

Supplement: Supplementary file 8 — Source data Fig. 6 [file 44319_2024_255_MOESM8_ESM.zip › Figure 6/6A/TRAP_NEAAs.tif]

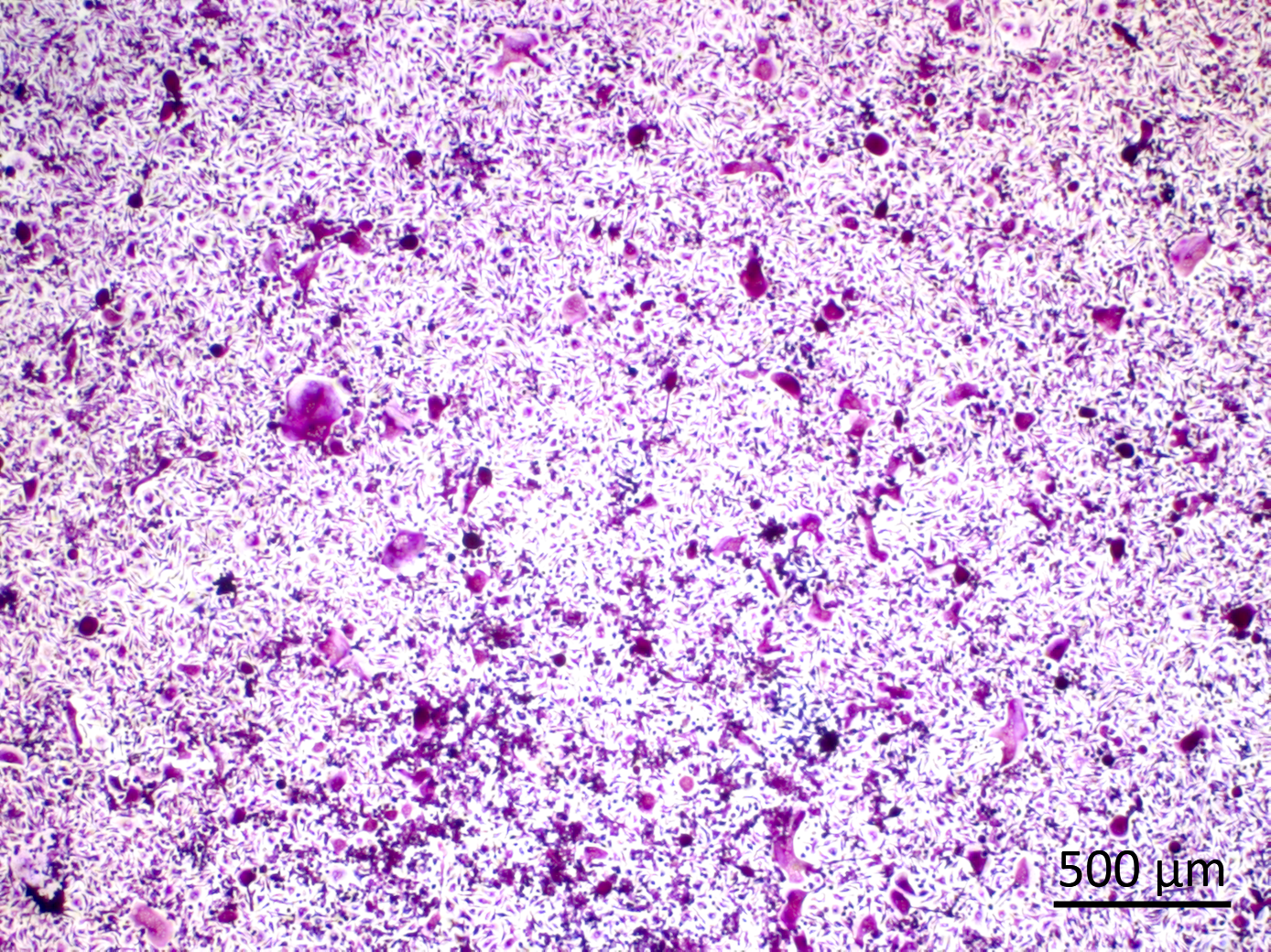

Supplement: Supplementary file 8 — Source data Fig. 6 [file 44319_2024_255_MOESM8_ESM.zip › Figure 6/6A/TRAP_Nucleotides.tif]

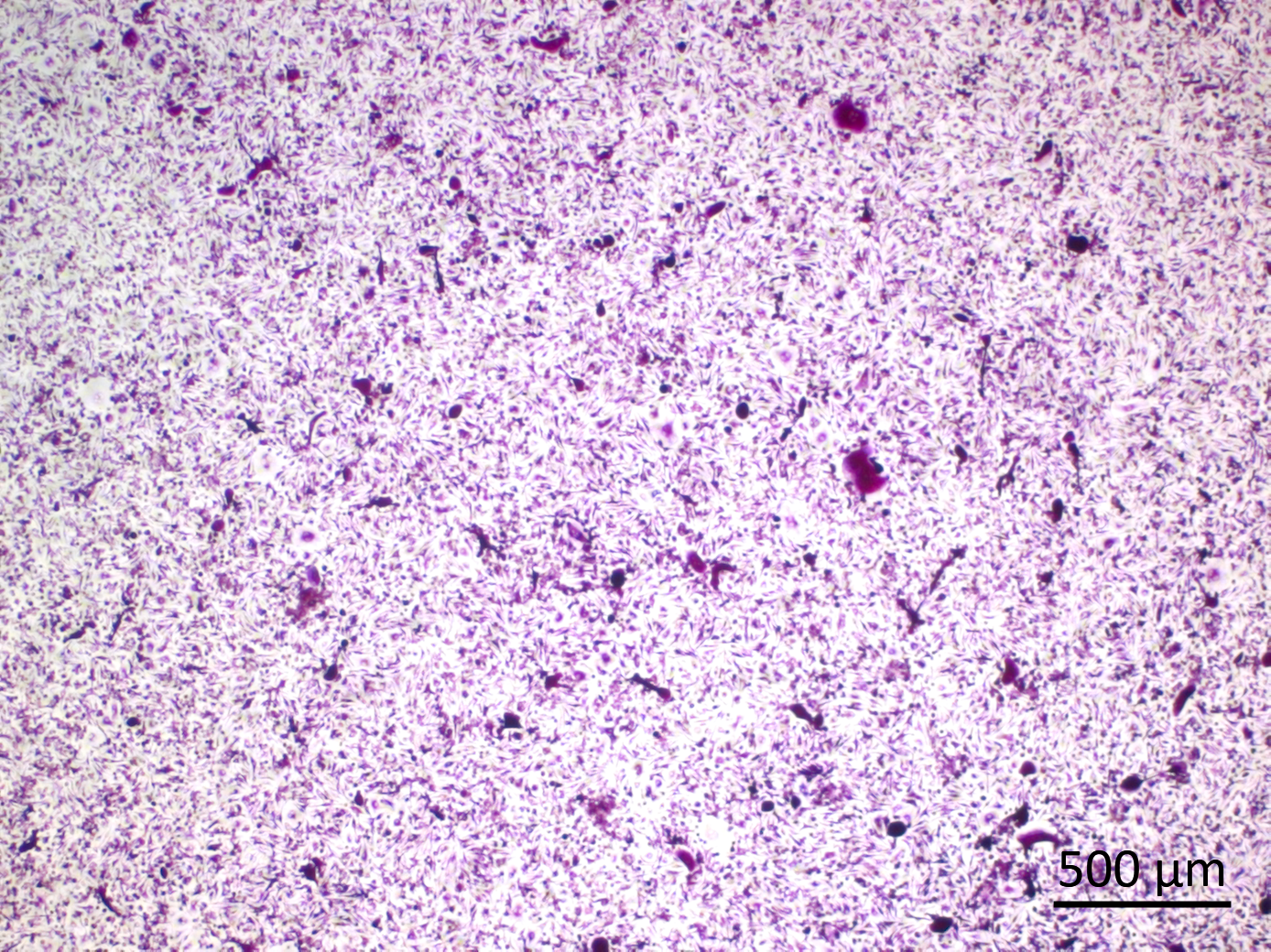

Supplement: Supplementary file 8 — Source data Fig. 6 [file 44319_2024_255_MOESM8_ESM.zip › Figure 6/6A/TRAP_Vehicle.tif]

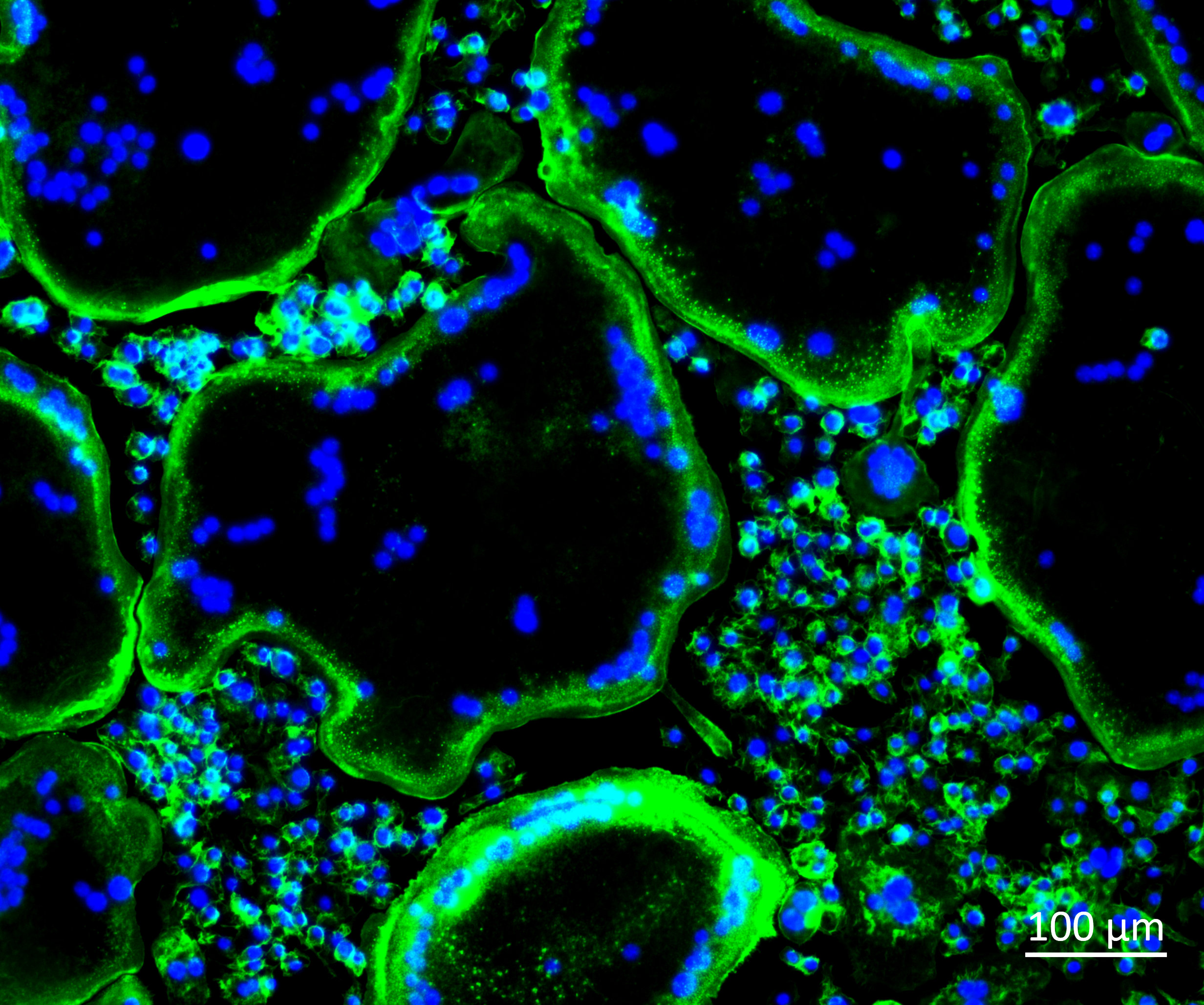

Supplement: Supplementary file 8 — Source data Fig. 6 [file 44319_2024_255_MOESM8_ESM.zip › Figure 6/6B/Actin ring_Control.tif]

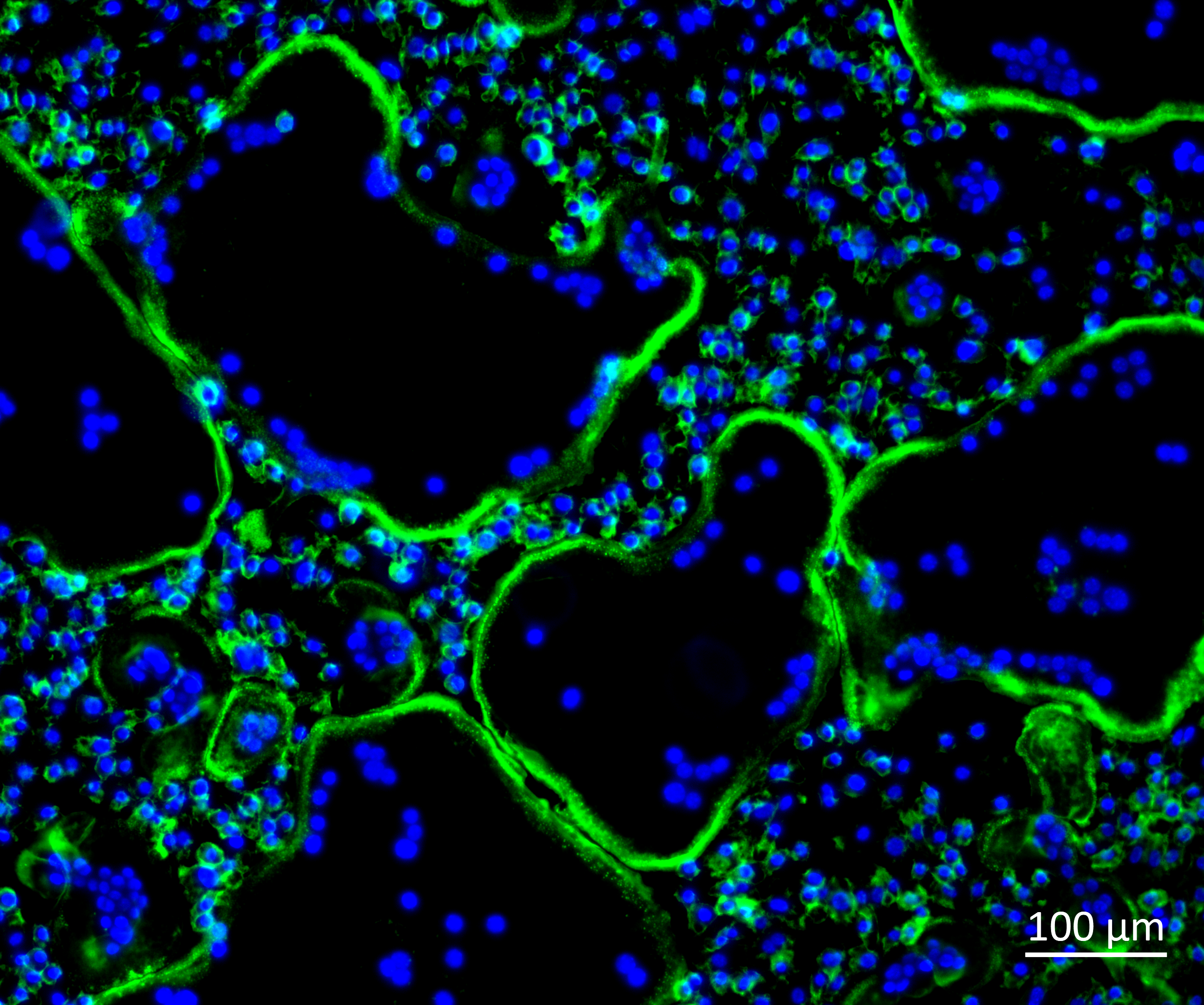

Supplement: Supplementary file 8 — Source data Fig. 6 [file 44319_2024_255_MOESM8_ESM.zip › Figure 6/6B/Actin ring_NEAAs&Nucleotides.tif]

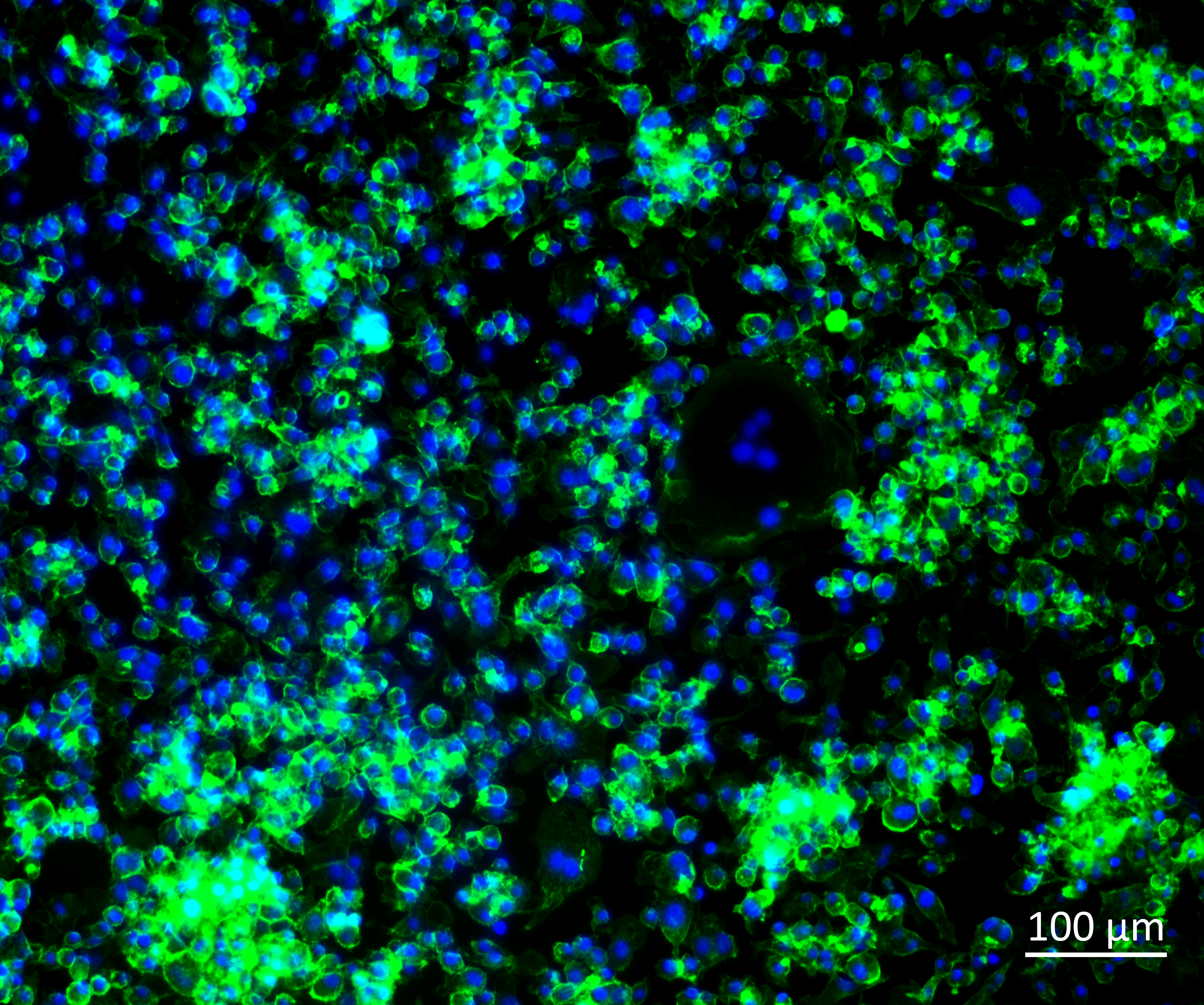

Supplement: Supplementary file 8 — Source data Fig. 6 [file 44319_2024_255_MOESM8_ESM.zip › Figure 6/6B/Actin ring_NEAAs.tif]

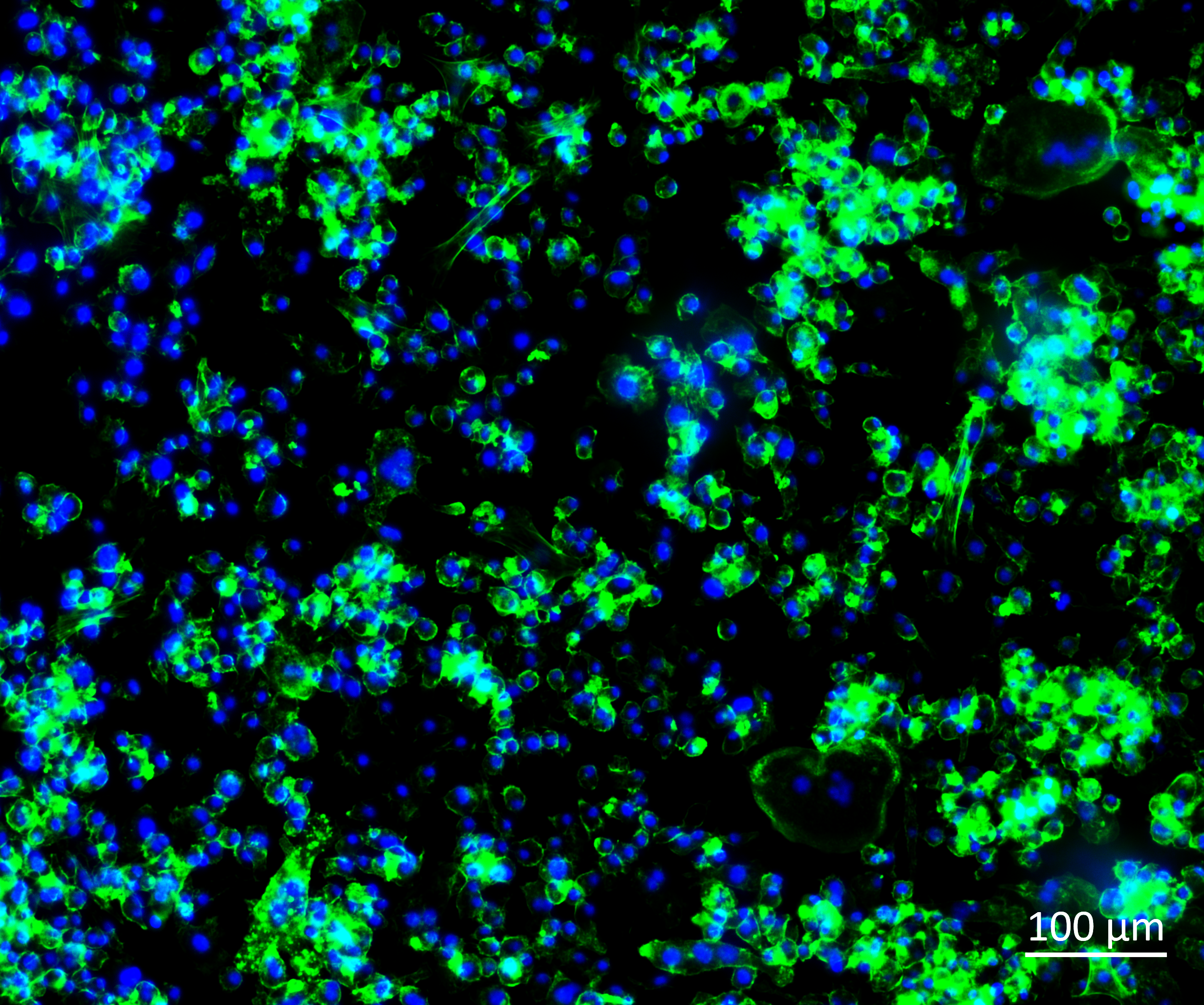

Supplement: Supplementary file 8 — Source data Fig. 6 [file 44319_2024_255_MOESM8_ESM.zip › Figure 6/6B/Actin ring_Nucleotides.tif]

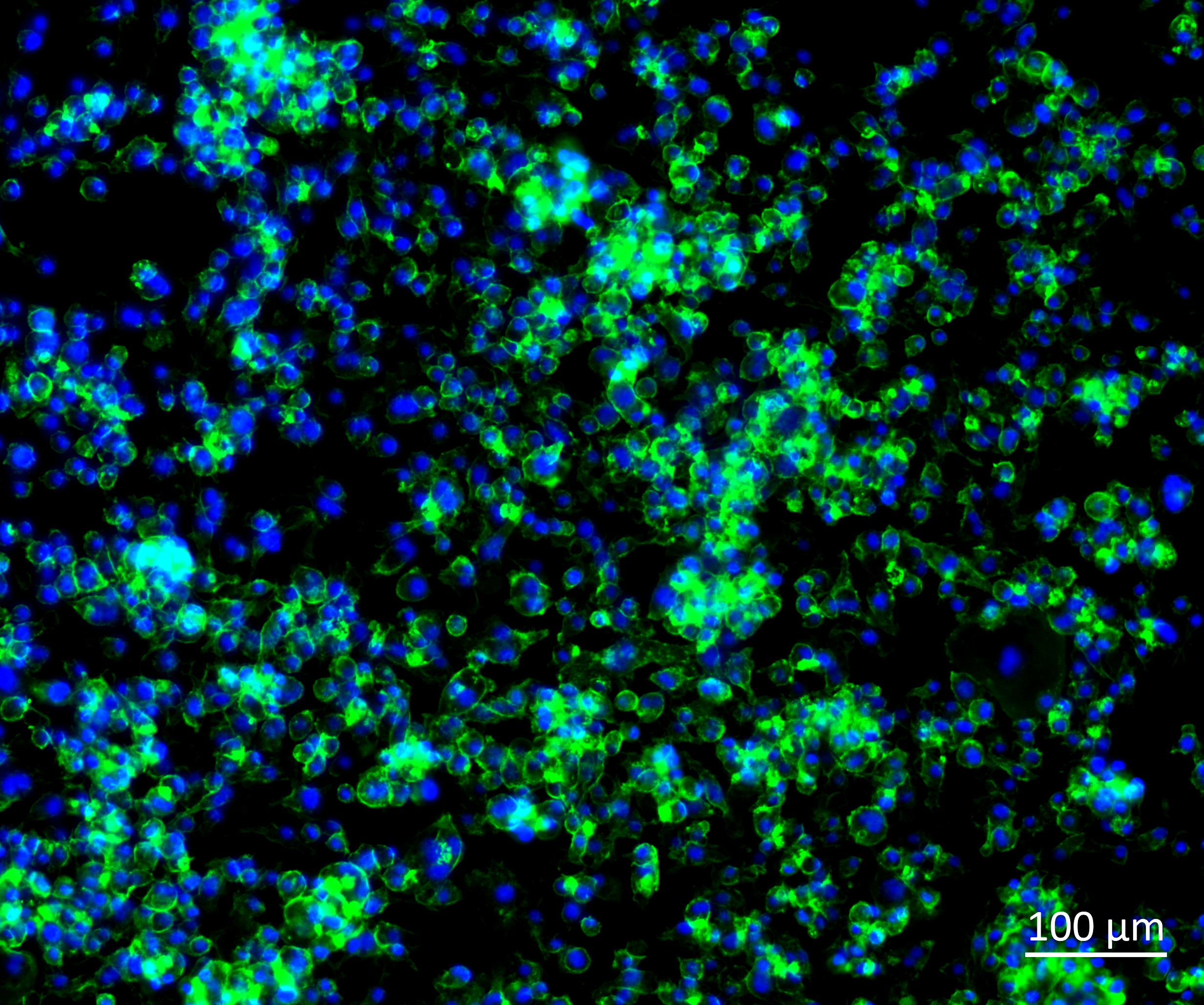

Supplement: Supplementary file 8 — Source data Fig. 6 [file 44319_2024_255_MOESM8_ESM.zip › Figure 6/6B/Actin ring_Vehicle.tif]

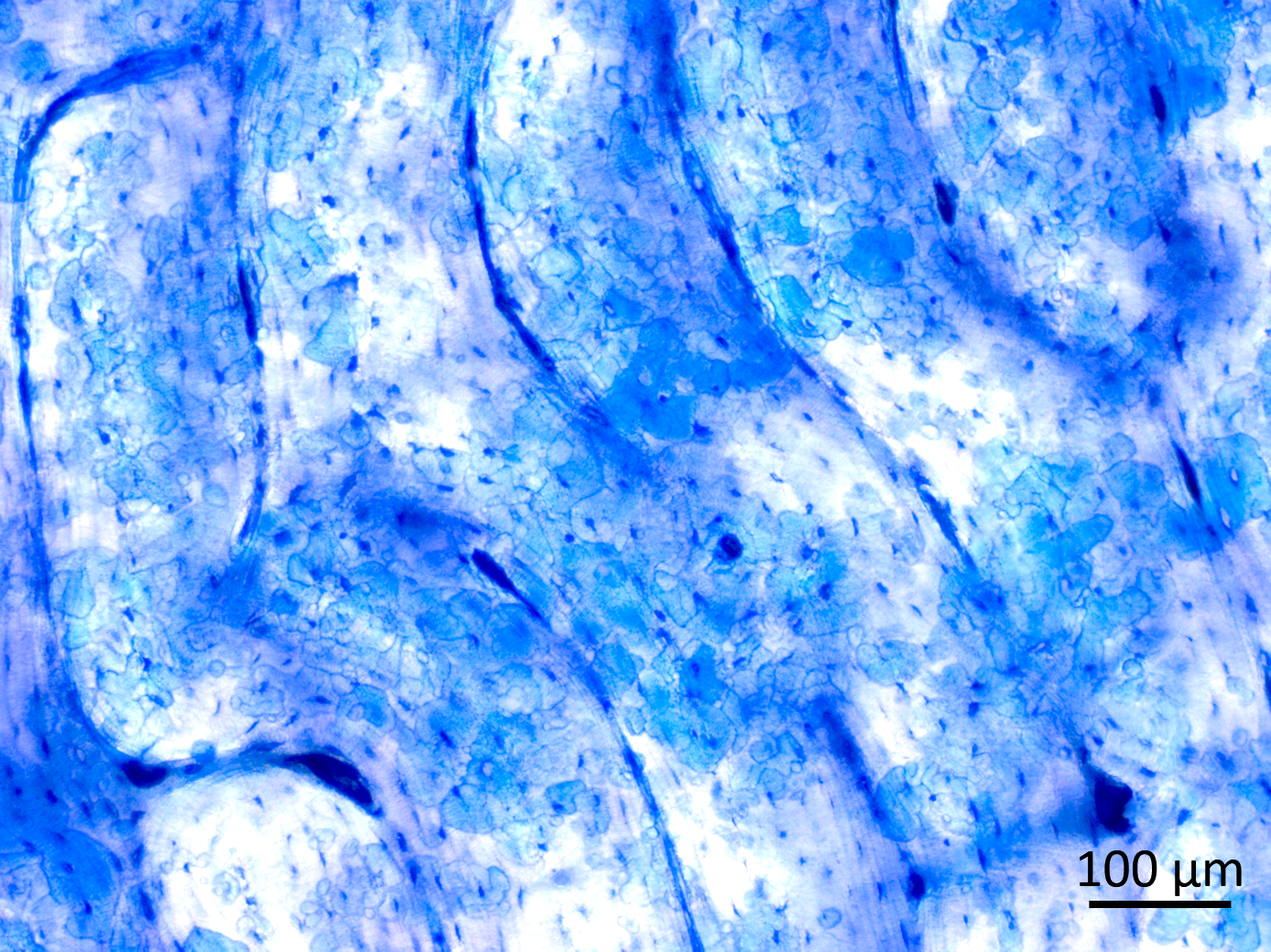

Supplement: Supplementary file 8 — Source data Fig. 6 [file 44319_2024_255_MOESM8_ESM.zip › Figure 6/6C/Pit assay_Control.tif]

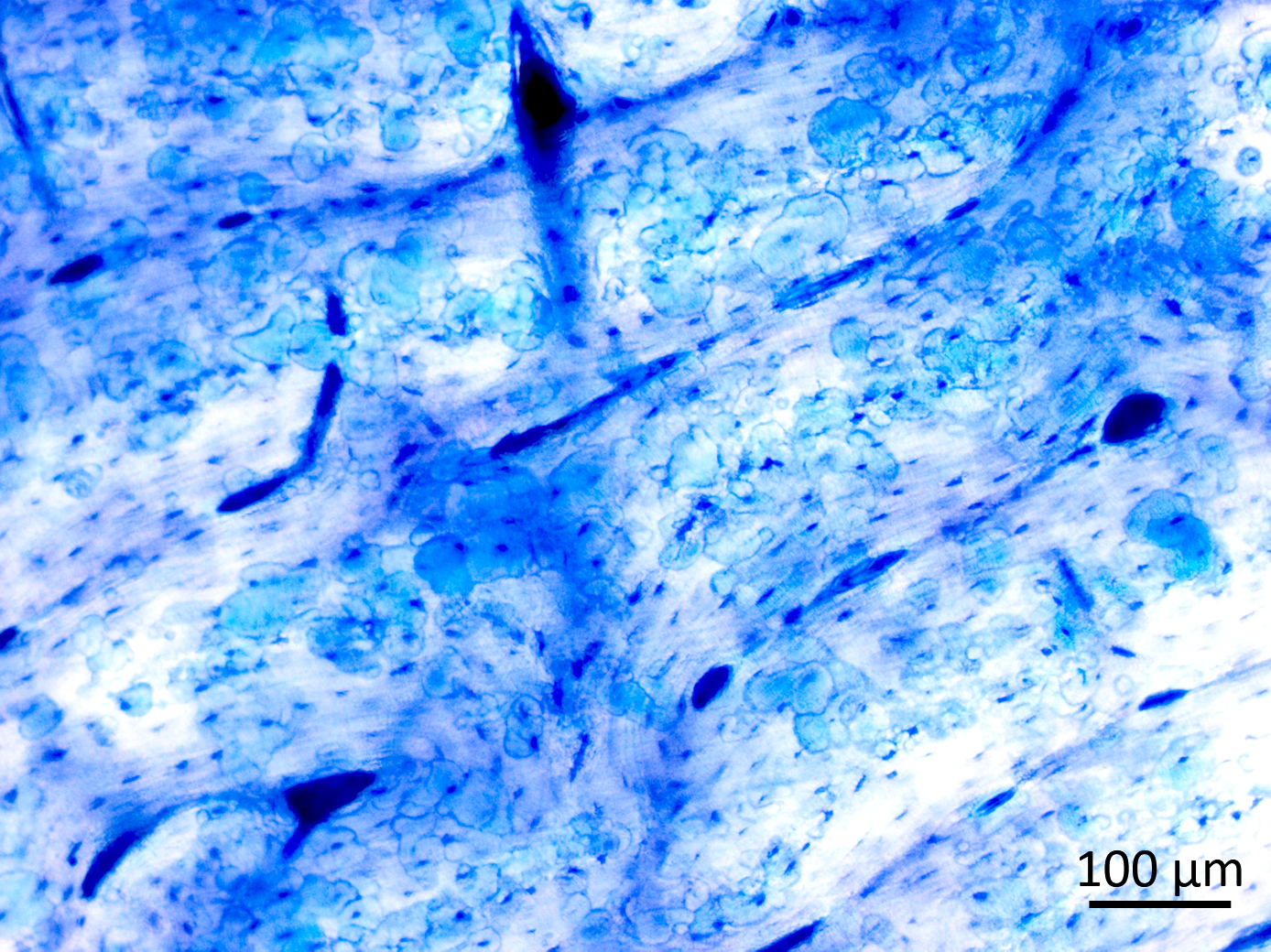

Supplement: Supplementary file 8 — Source data Fig. 6 [file 44319_2024_255_MOESM8_ESM.zip › Figure 6/6C/Pit assay_NEAAs&Nucleotides.tif]

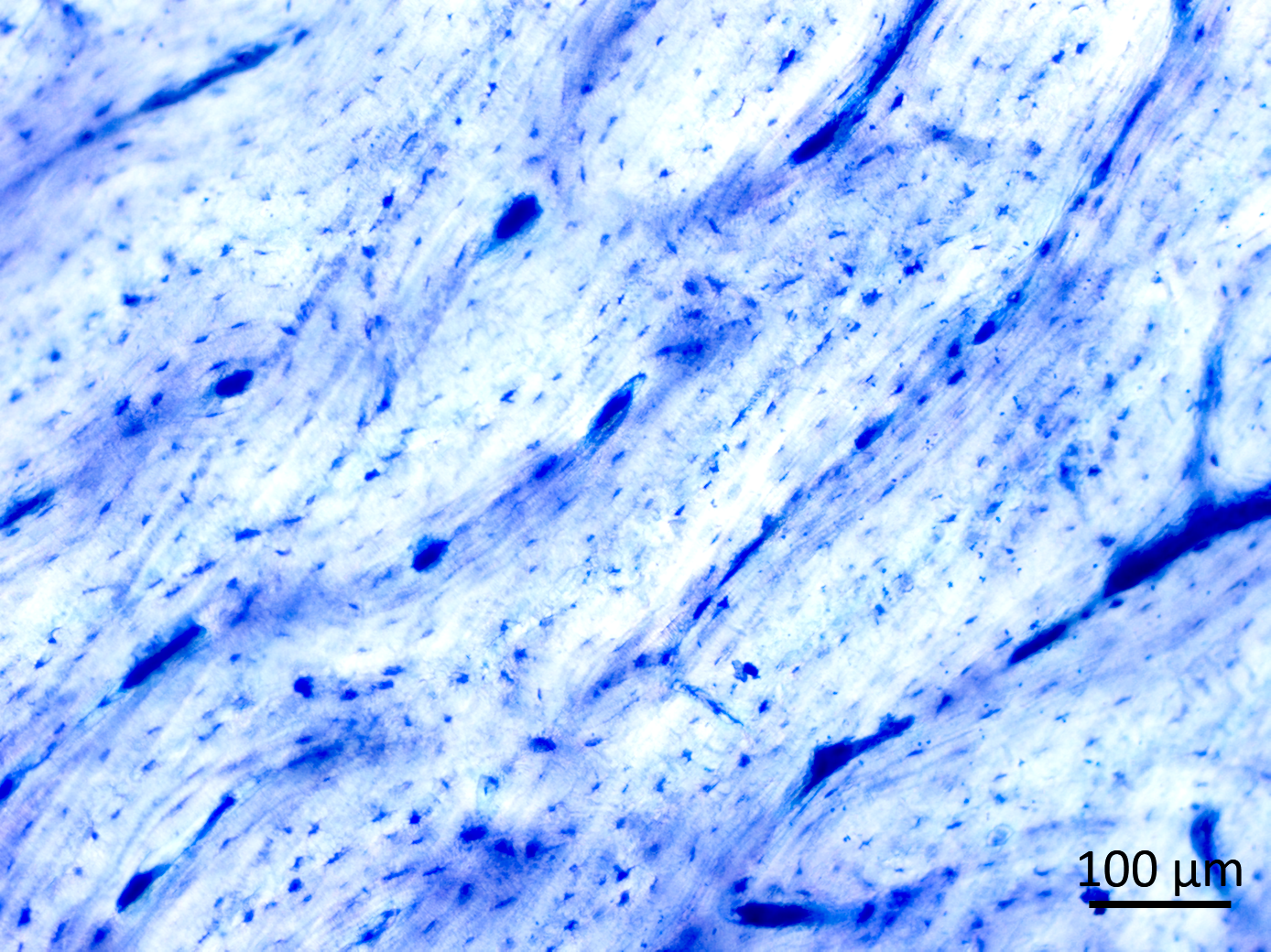

Supplement: Supplementary file 8 — Source data Fig. 6 [file 44319_2024_255_MOESM8_ESM.zip › Figure 6/6C/Pit assay_NEAAs.tif]

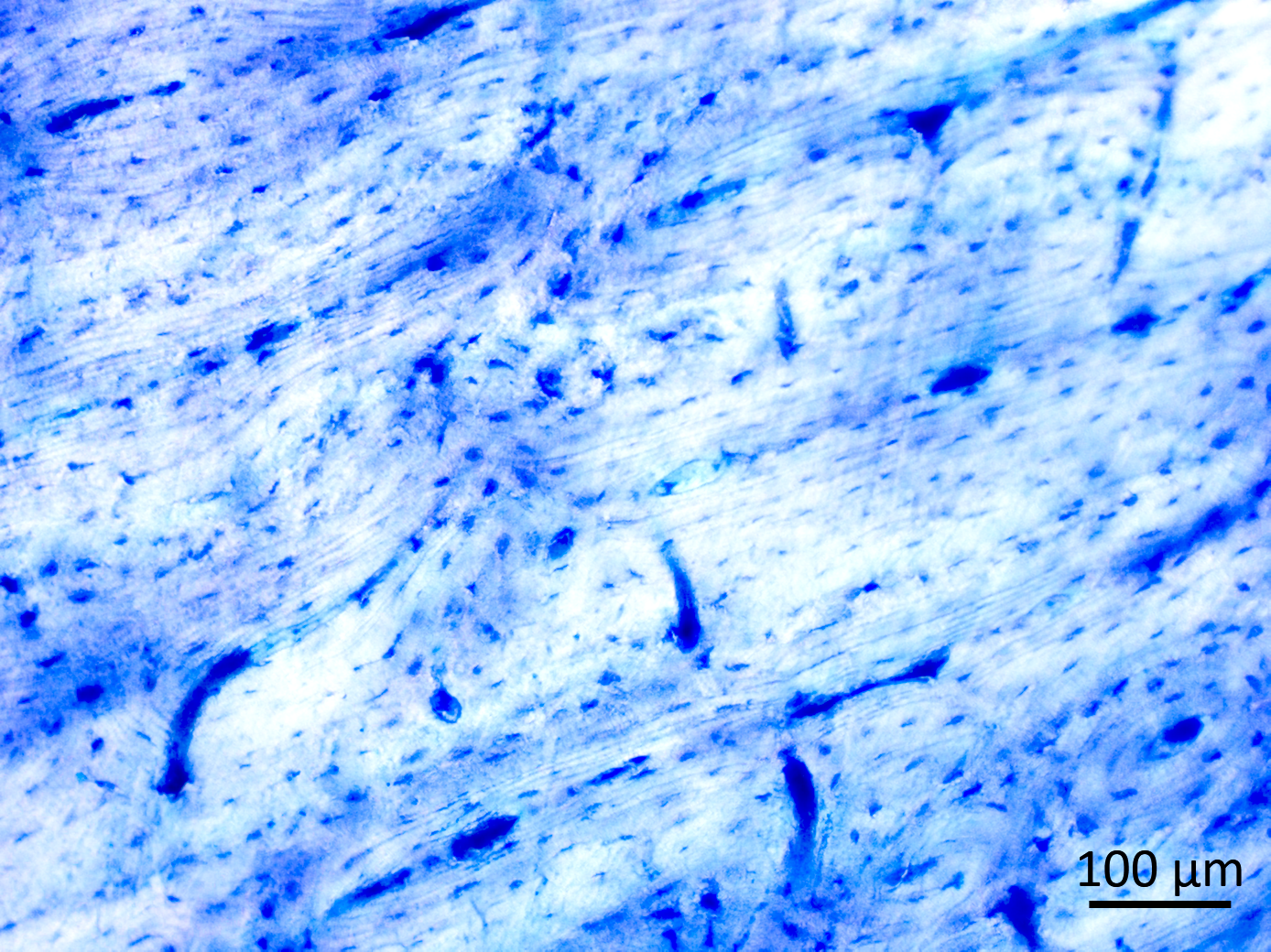

Supplement: Supplementary file 8 — Source data Fig. 6 [file 44319_2024_255_MOESM8_ESM.zip › Figure 6/6C/Pit assay_Nucleotides.tif]

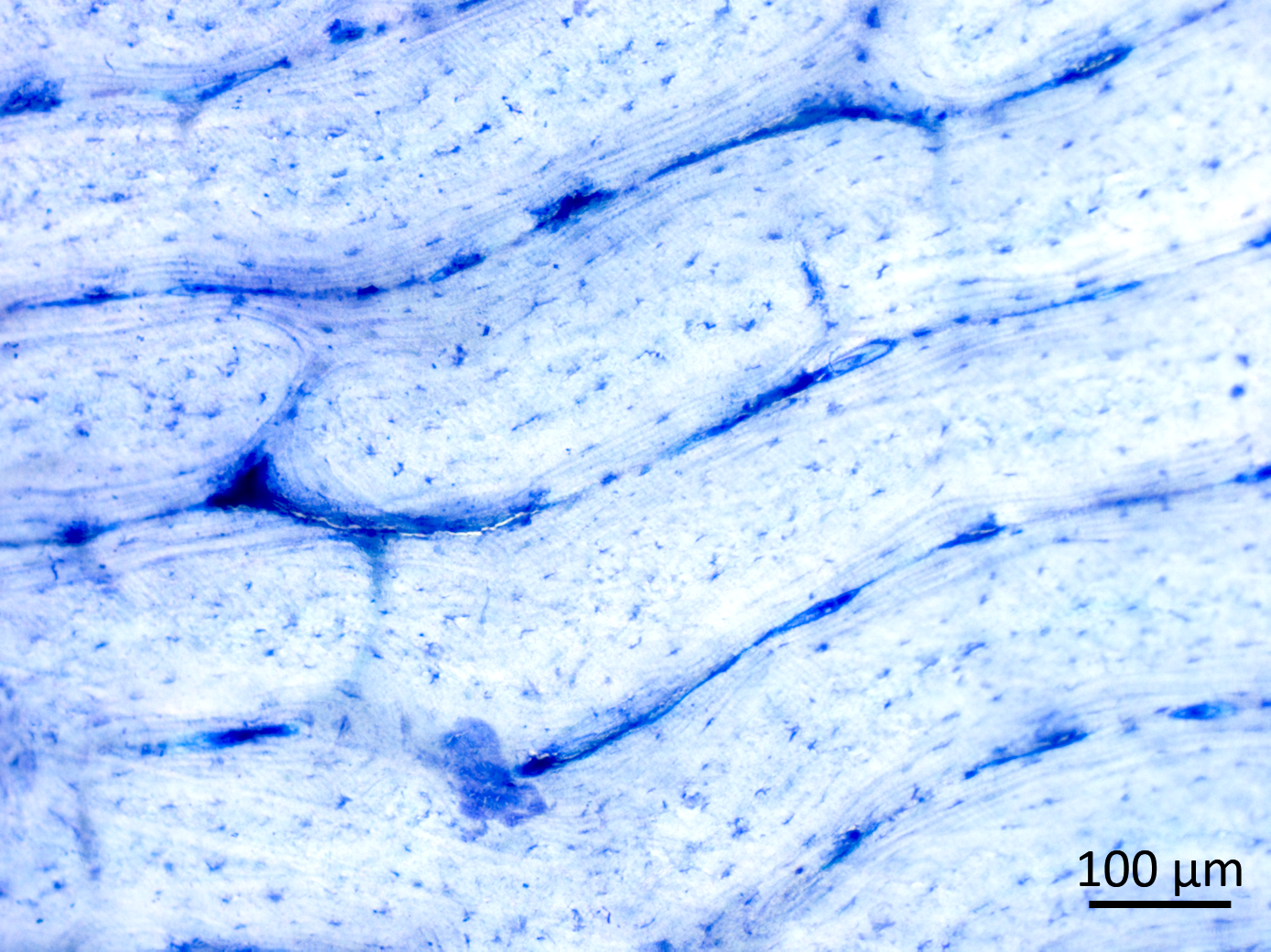

Supplement: Supplementary file 8 — Source data Fig. 6 [file 44319_2024_255_MOESM8_ESM.zip › Figure 6/6C/Pit assay_Vehicle.tif]

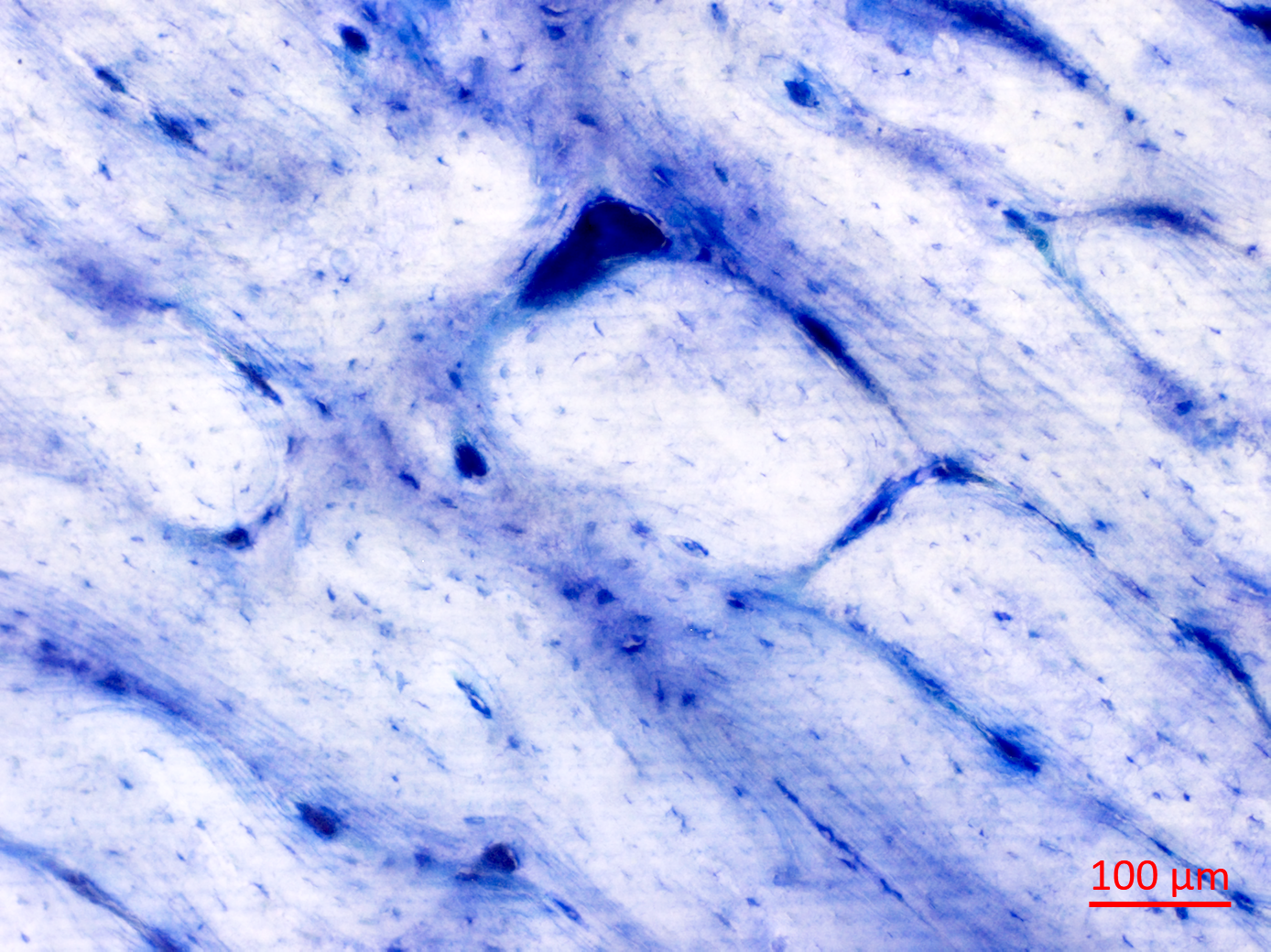

Supplement: Supplementary file 9 — Source data Fig. 7 [file 44319_2024_255_MOESM9_ESM.zip › Figure 7/7K/Pit assay_AOA.tif]

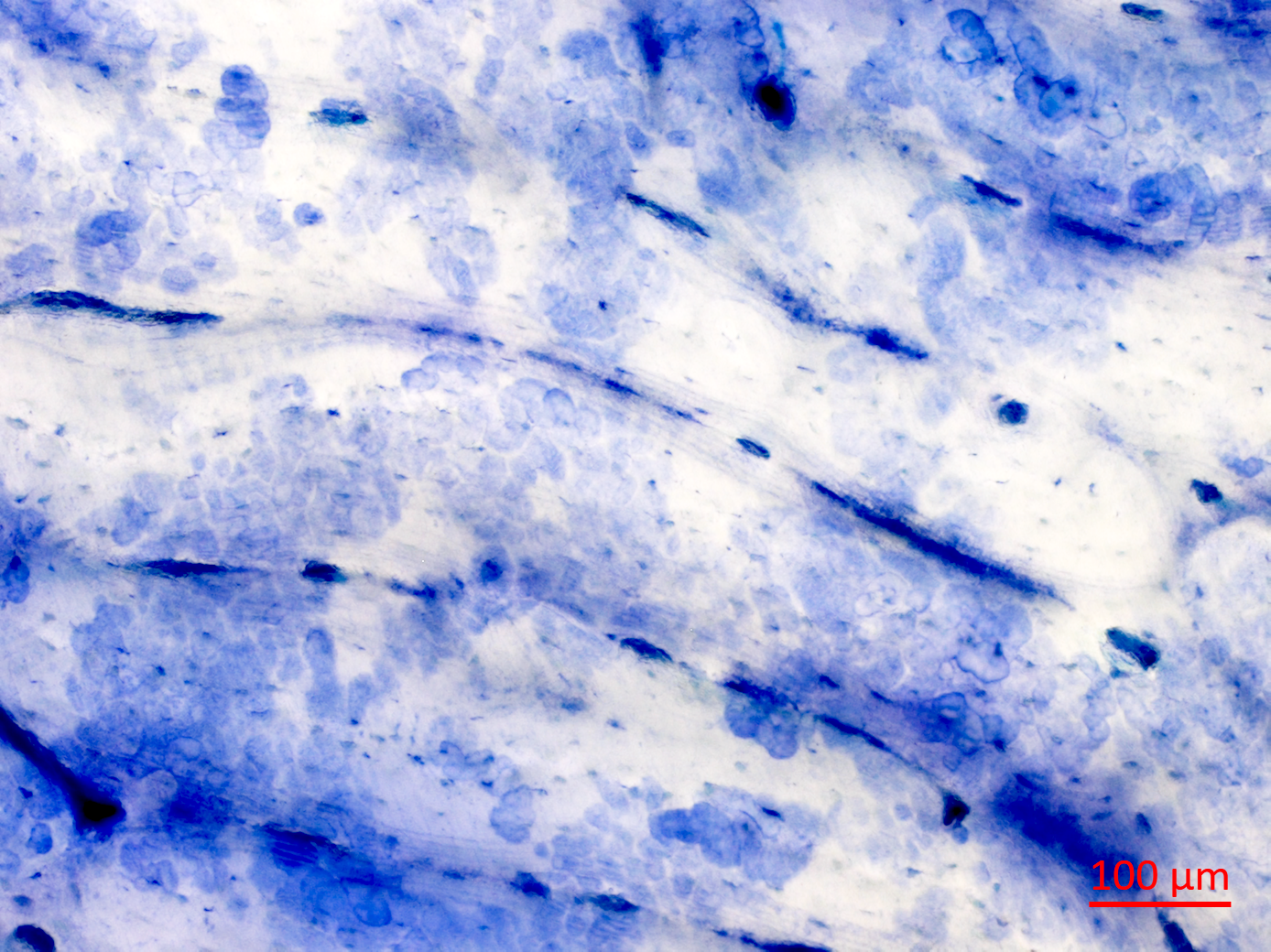

Supplement: Supplementary file 9 — Source data Fig. 7 [file 44319_2024_255_MOESM9_ESM.zip › Figure 7/7K/Pit assay_Vehicle.tif]

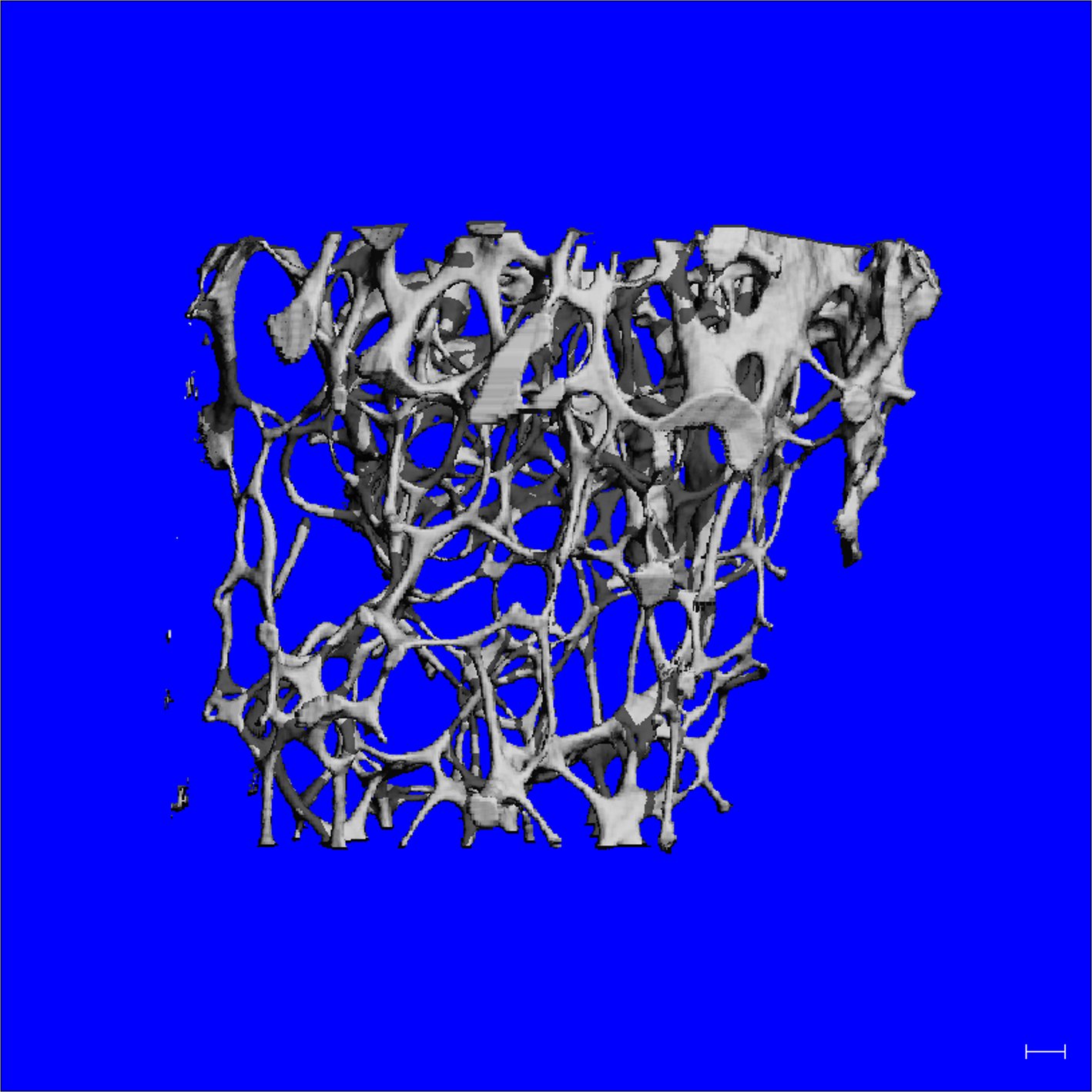

Supplement: Supplementary file 10 — Source data Fig. 8 [file 44319_2024_255_MOESM10_ESM.zip › Figure 8/8B/microCT_Sham-vehicle.tif]

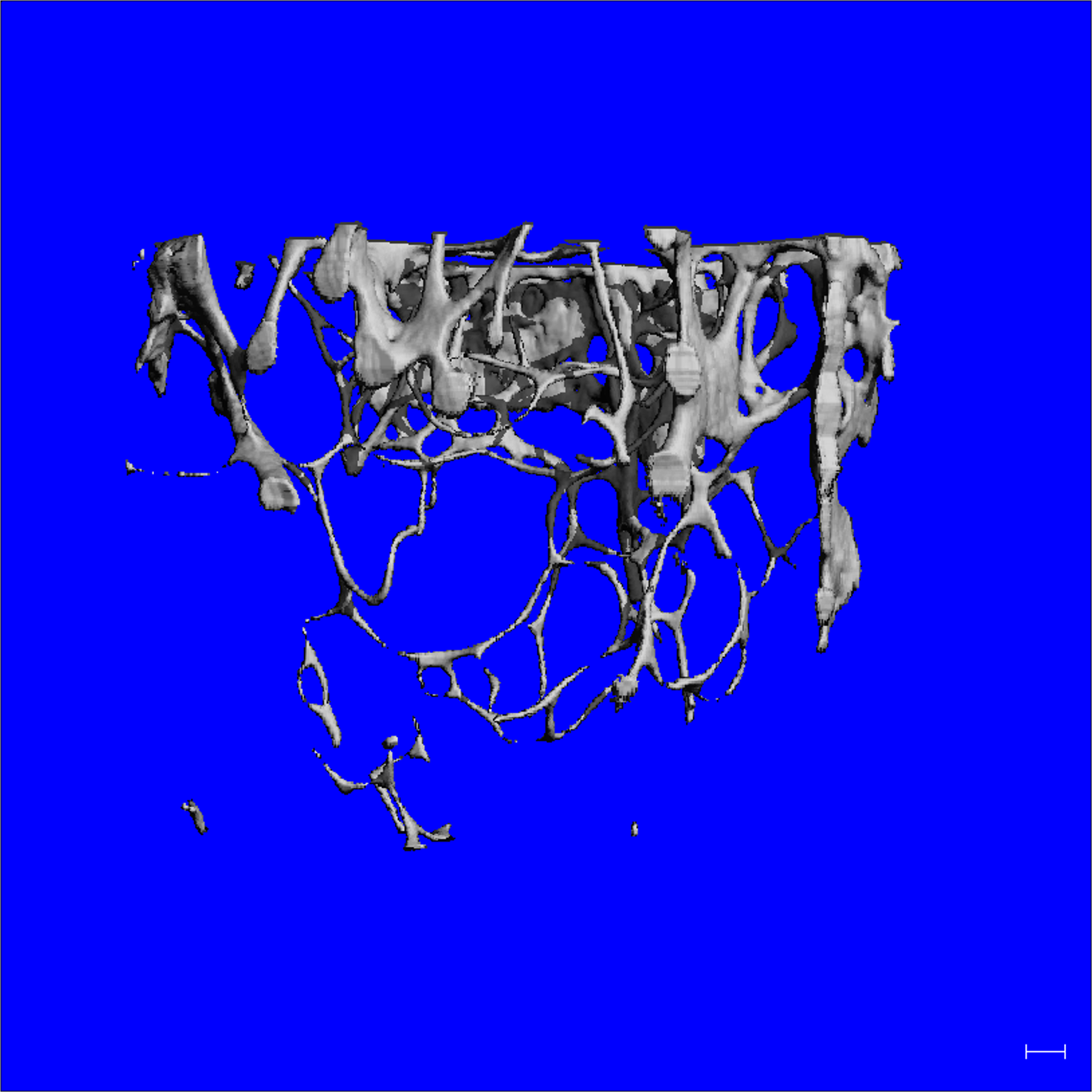

Supplement: Supplementary file 10 — Source data Fig. 8 [file 44319_2024_255_MOESM10_ESM.zip › Figure 8/8C/microCT_OVX-vehicle.tif]

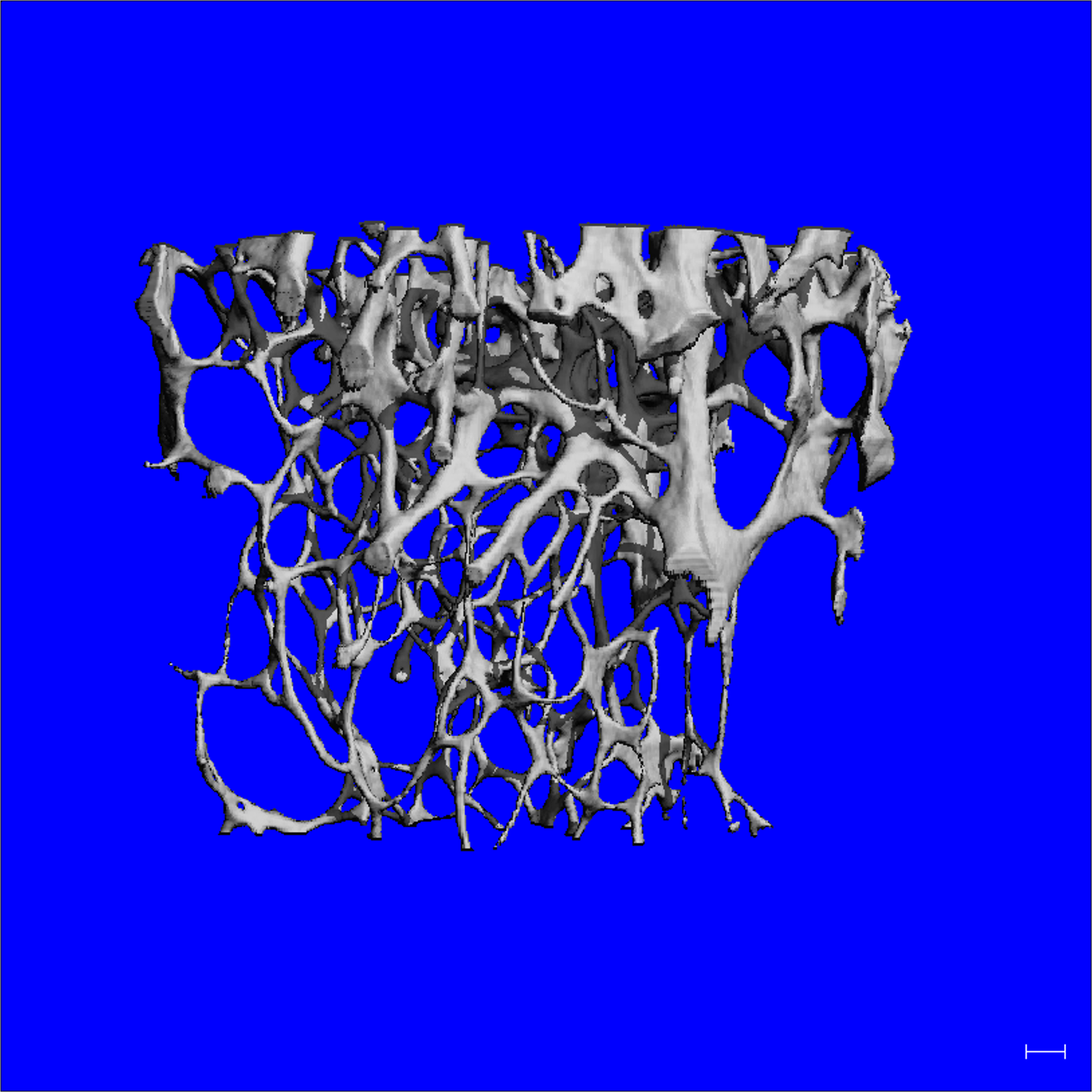

Supplement: Supplementary file 10 — Source data Fig. 8 [file 44319_2024_255_MOESM10_ESM.zip › Figure 8/8D/microCT_OVX-200mg.tif]

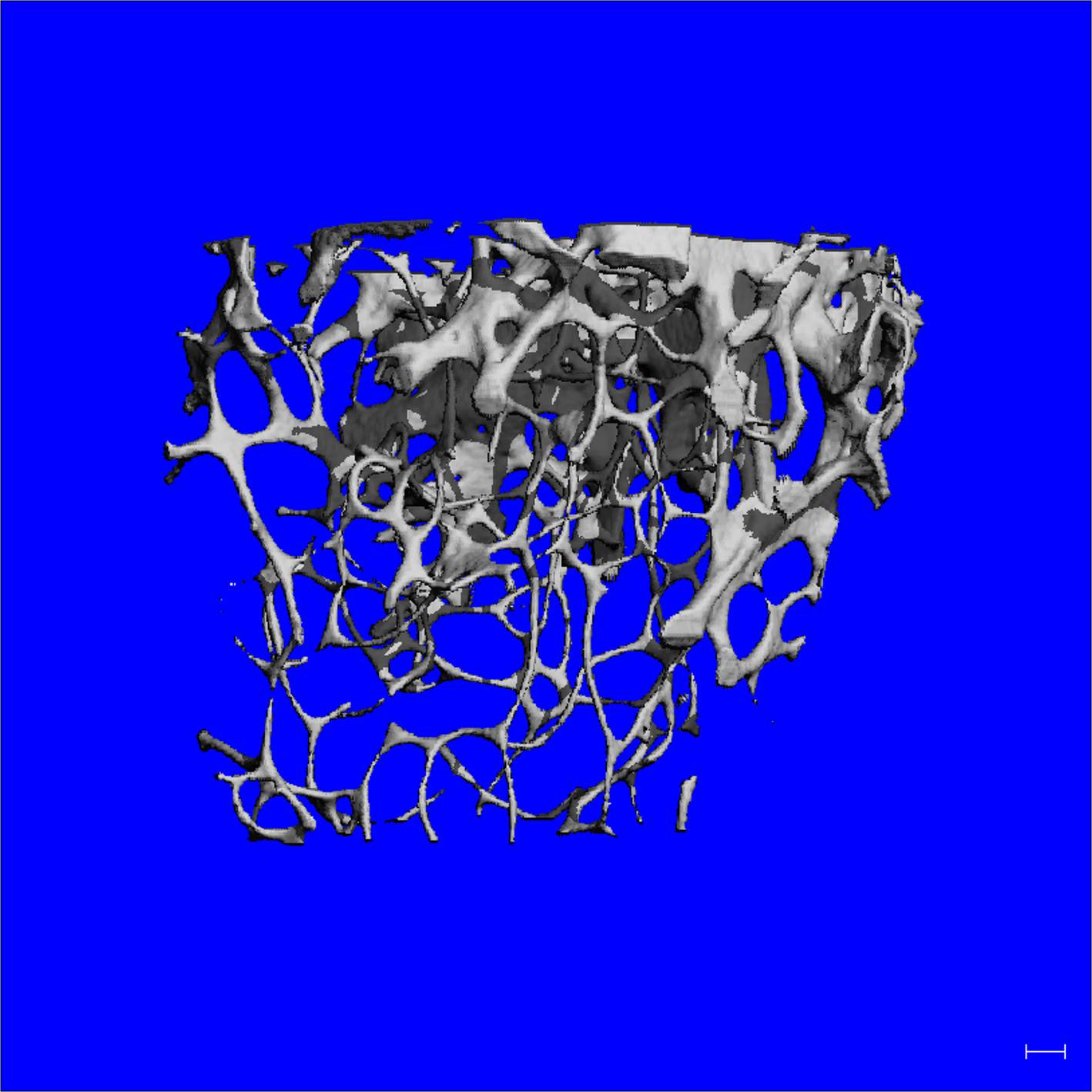

Supplement: Supplementary file 10 — Source data Fig. 8 [file 44319_2024_255_MOESM10_ESM.zip › Figure 8/8E/microCT_OVX-400mg.tif]

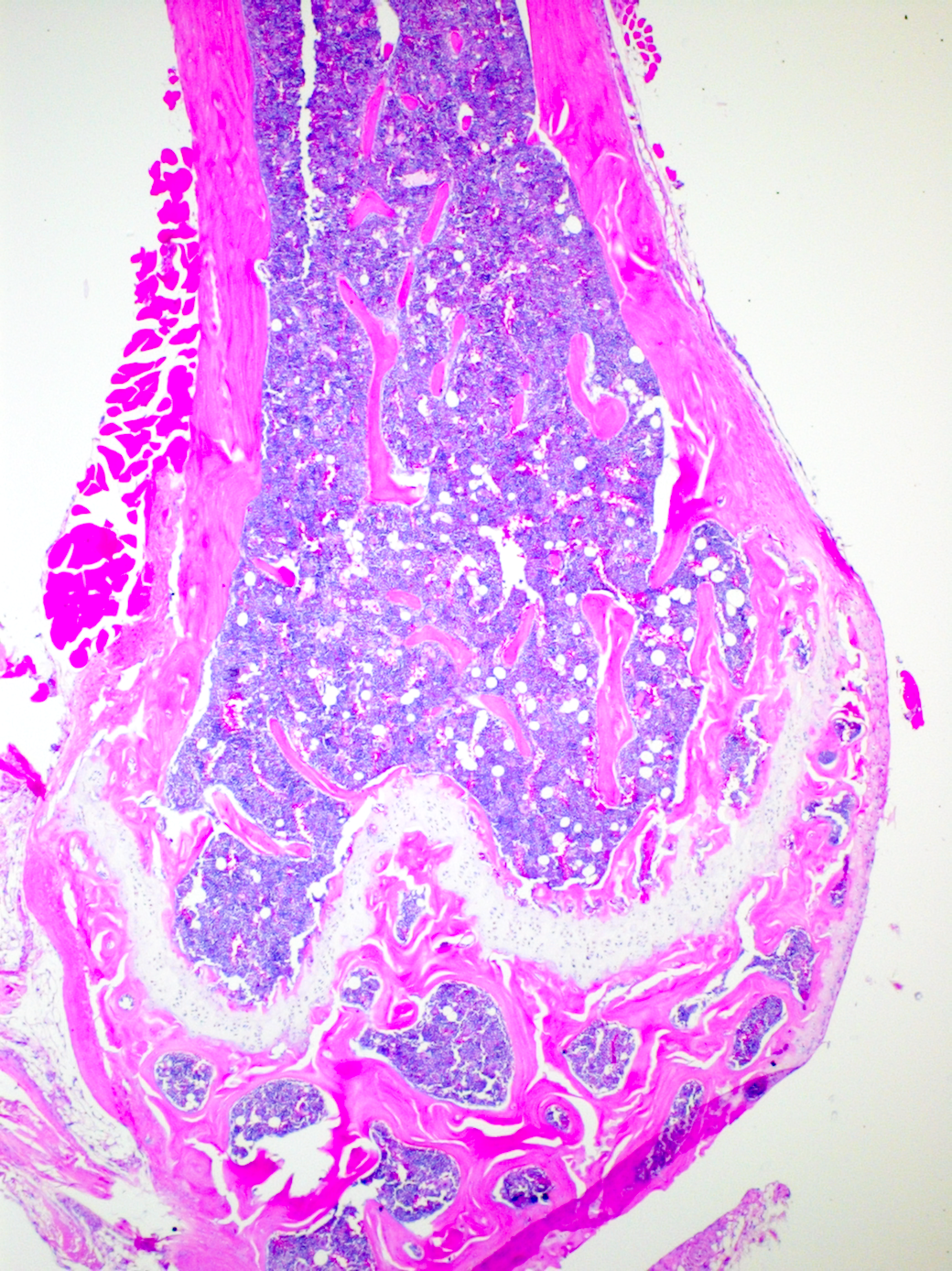

Supplement: Supplementary file 10 — Source data Fig. 8 [file 44319_2024_255_MOESM10_ESM.zip › Figure 8/8G/H&E_Sham-vehicle.tif]

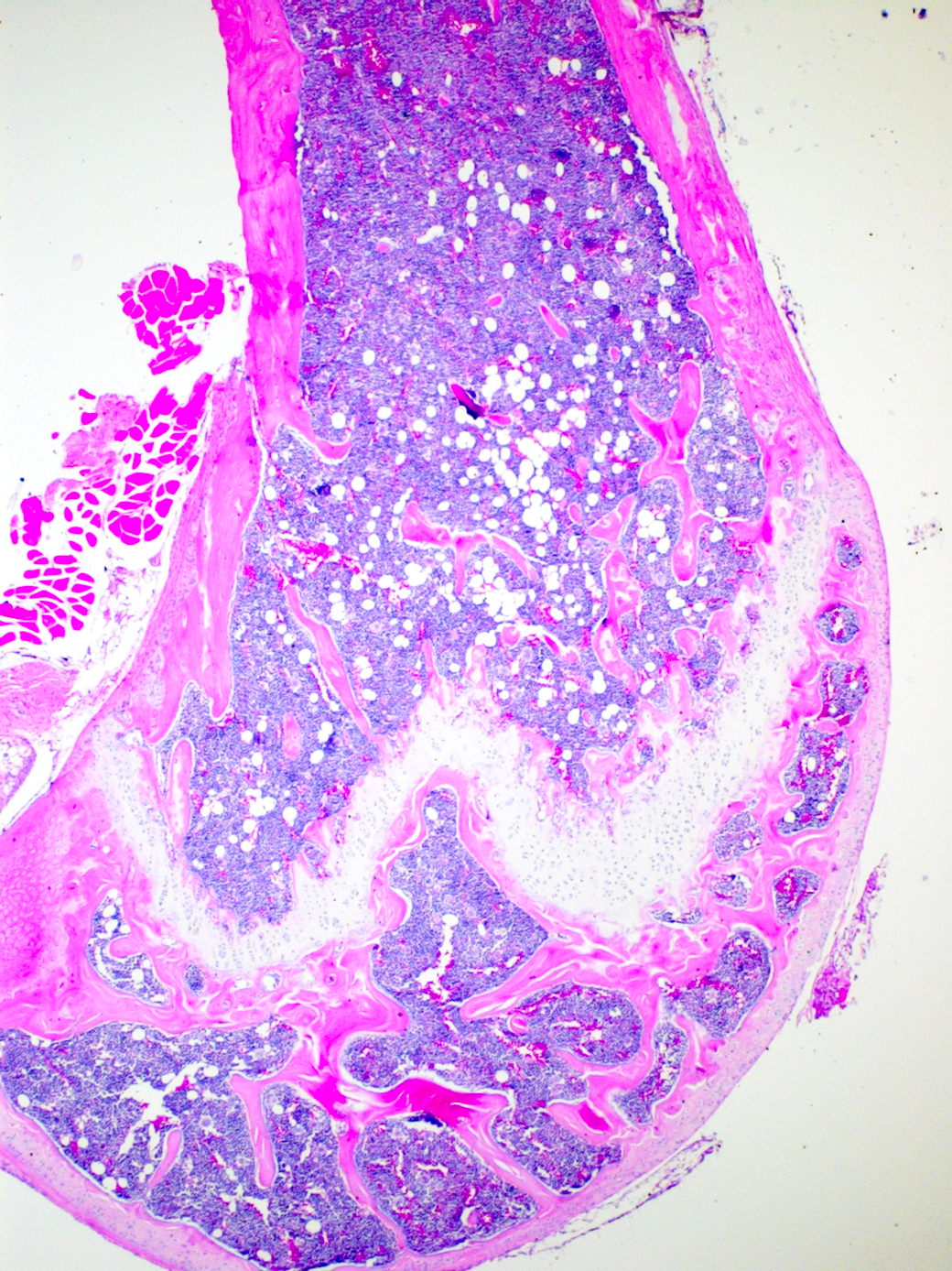

Supplement: Supplementary file 10 — Source data Fig. 8 [file 44319_2024_255_MOESM10_ESM.zip › Figure 8/8H/H&E_OVX-vehicle.tif]

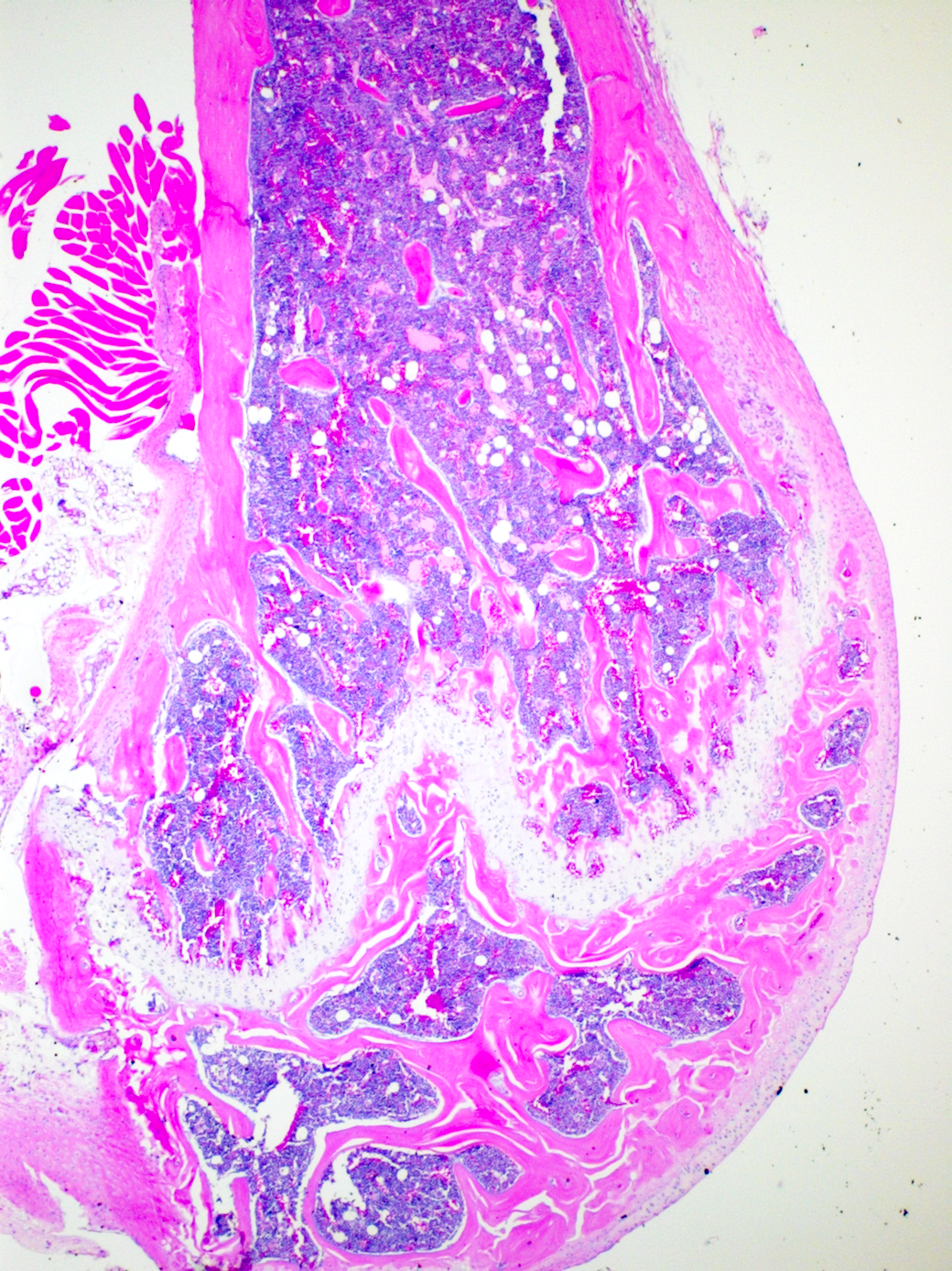

Supplement: Supplementary file 10 — Source data Fig. 8 [file 44319_2024_255_MOESM10_ESM.zip › Figure 8/8I/H&E_OVX-200mg.tif]

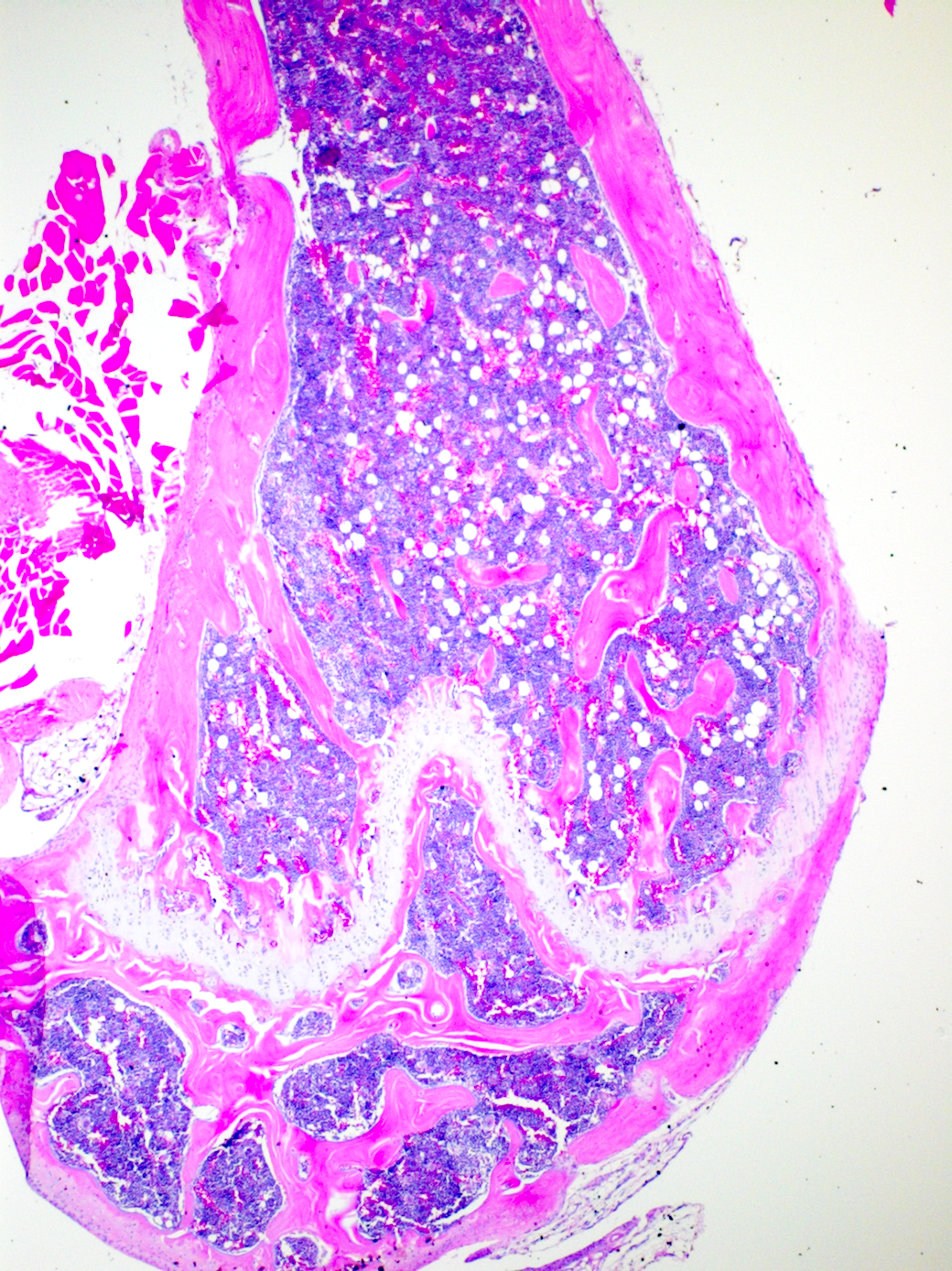

Supplement: Supplementary file 10 — Source data Fig. 8 [file 44319_2024_255_MOESM10_ESM.zip › Figure 8/8J/H&E_OVX-400mg.tif]

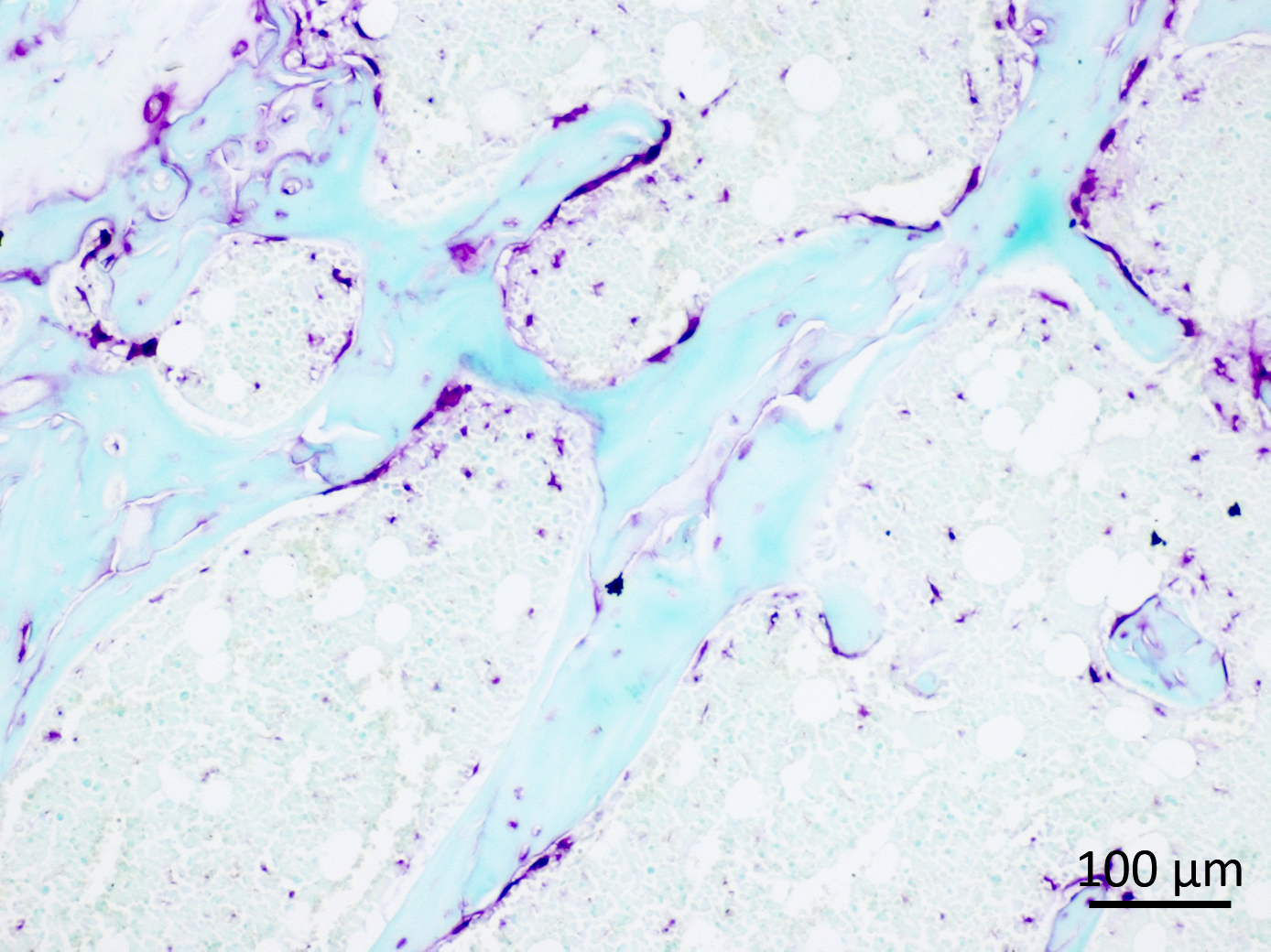

Supplement: Supplementary file 10 — Source data Fig. 8 [file 44319_2024_255_MOESM10_ESM.zip › Figure 8/8K/TRAP_Sham-vehicle.tif]

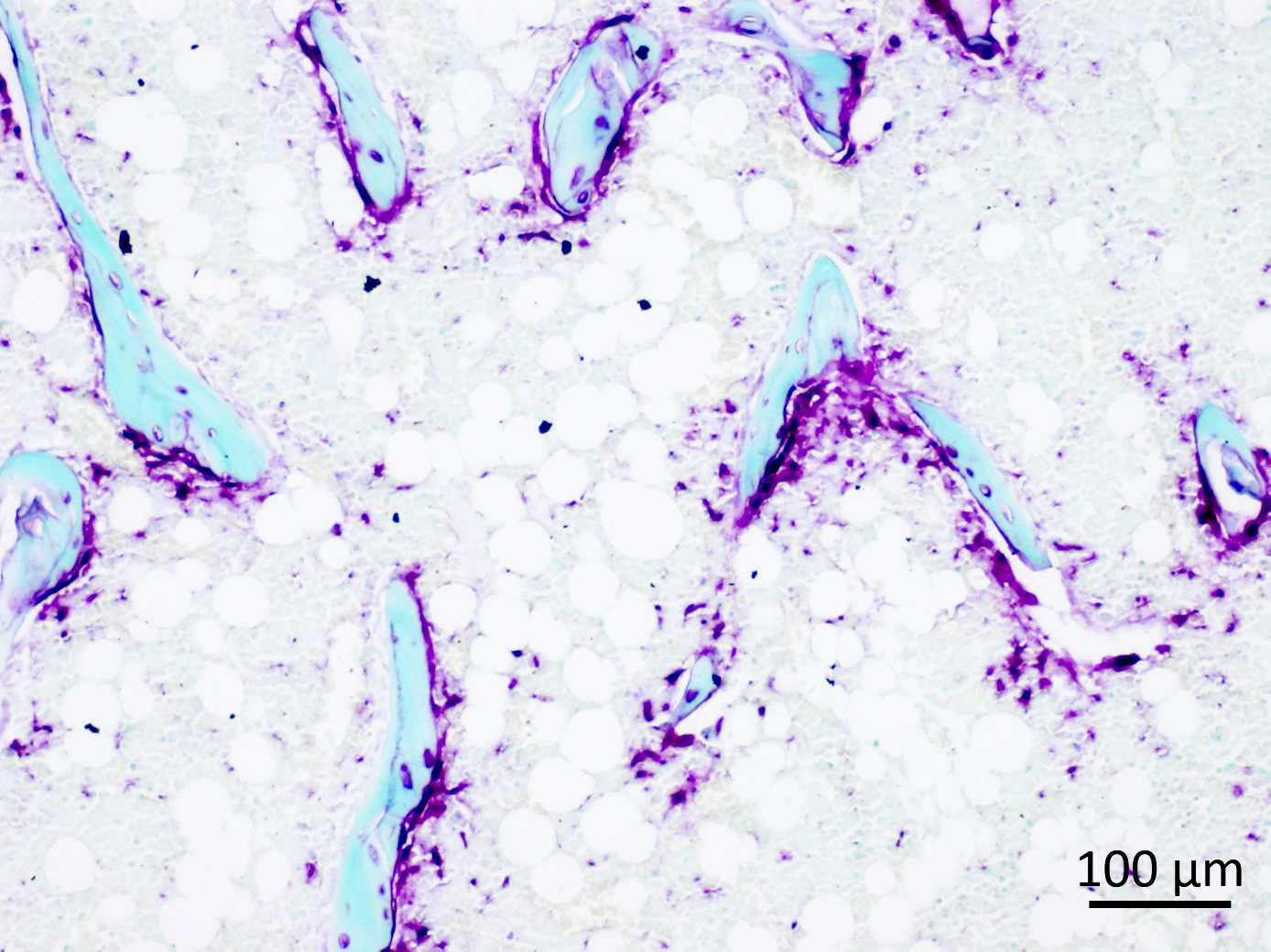

Supplement: Supplementary file 10 — Source data Fig. 8 [file 44319_2024_255_MOESM10_ESM.zip › Figure 8/8L/TRAP_OVX-vehicle.tif]

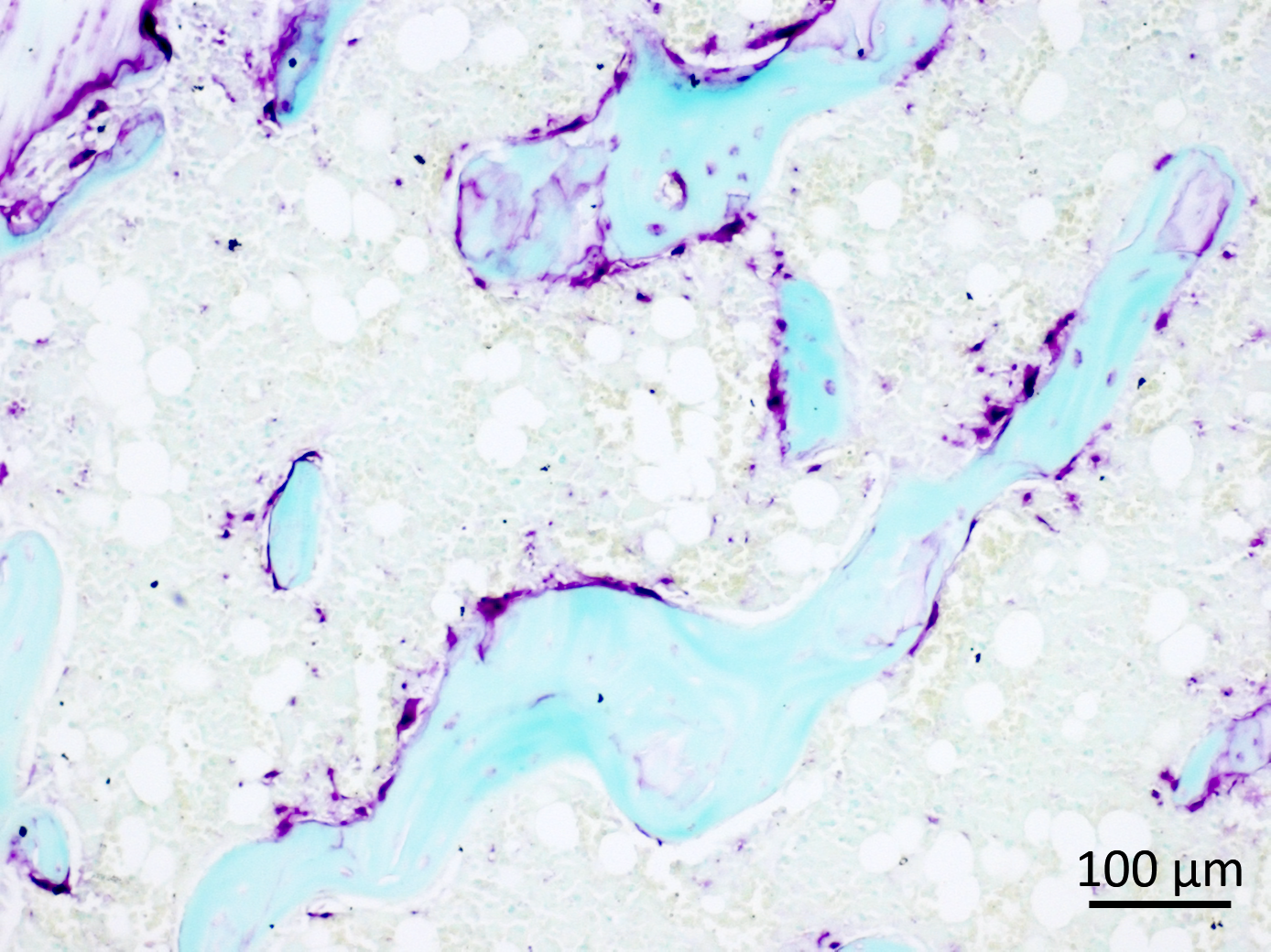

Supplement: Supplementary file 10 — Source data Fig. 8 [file 44319_2024_255_MOESM10_ESM.zip › Figure 8/8M/TRAP_OVX-200mg.tif]

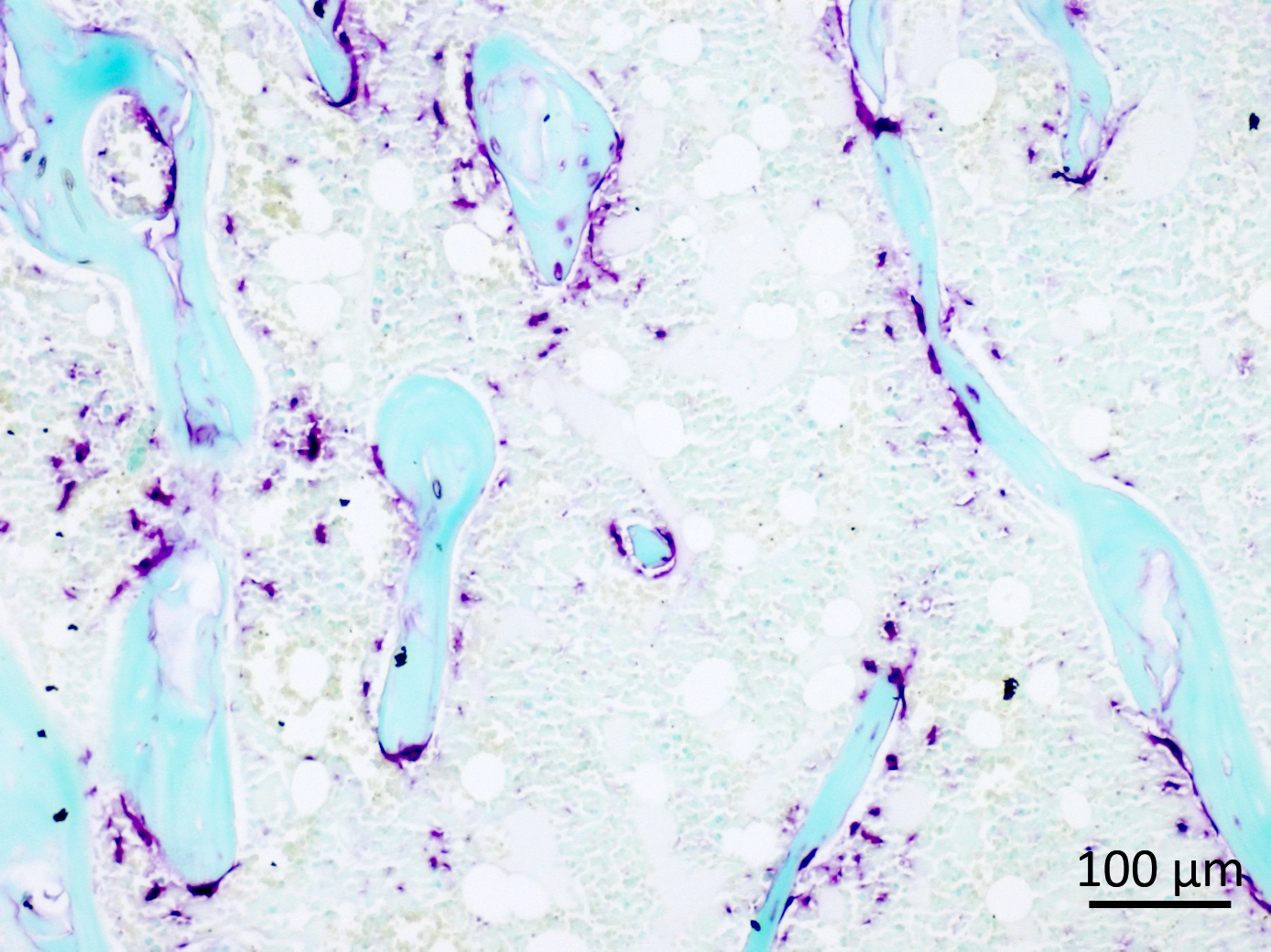

Supplement: Supplementary file 10 — Source data Fig. 8 [file 44319_2024_255_MOESM10_ESM.zip › Figure 8/8N/TRAP_OVX-400mg.tif]

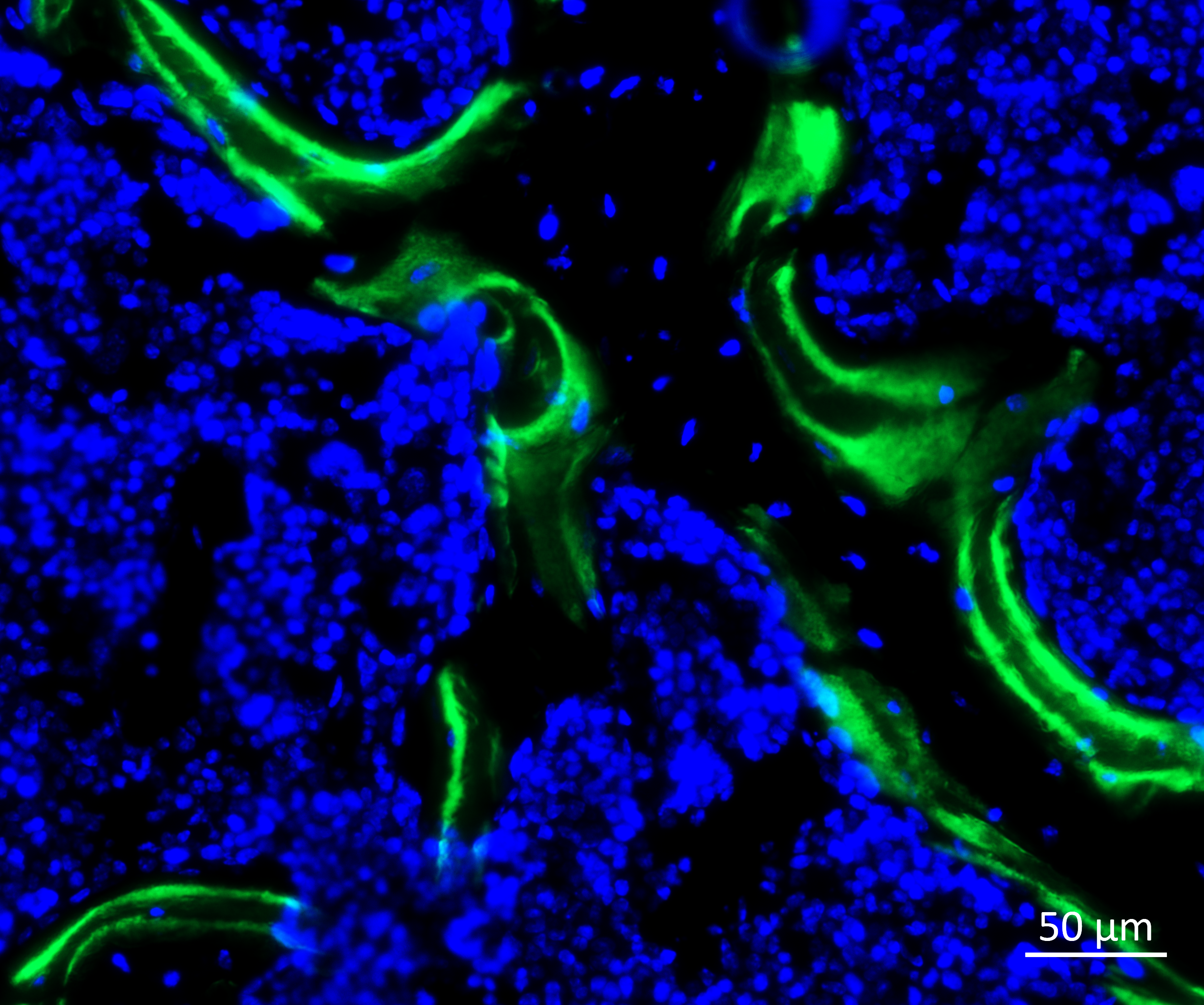

Supplement: Supplementary file 10 — Source data Fig. 8 [file 44319_2024_255_MOESM10_ESM.zip › Figure 8/8Q/Double labeling_Sham-vehicle.tif]

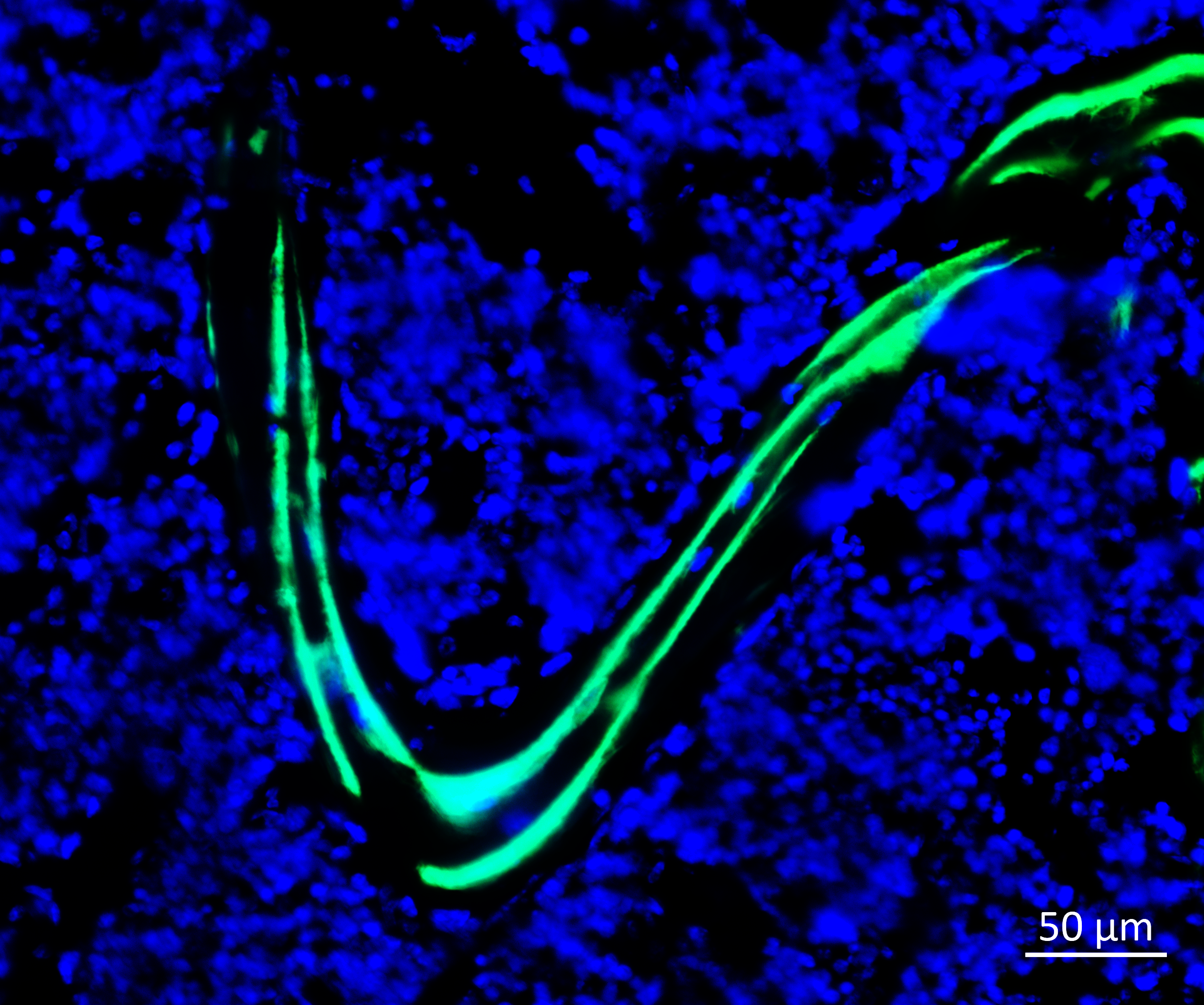

Supplement: Supplementary file 10 — Source data Fig. 8 [file 44319_2024_255_MOESM10_ESM.zip › Figure 8/8R/Double labeling_OVX-vehicle.tif]

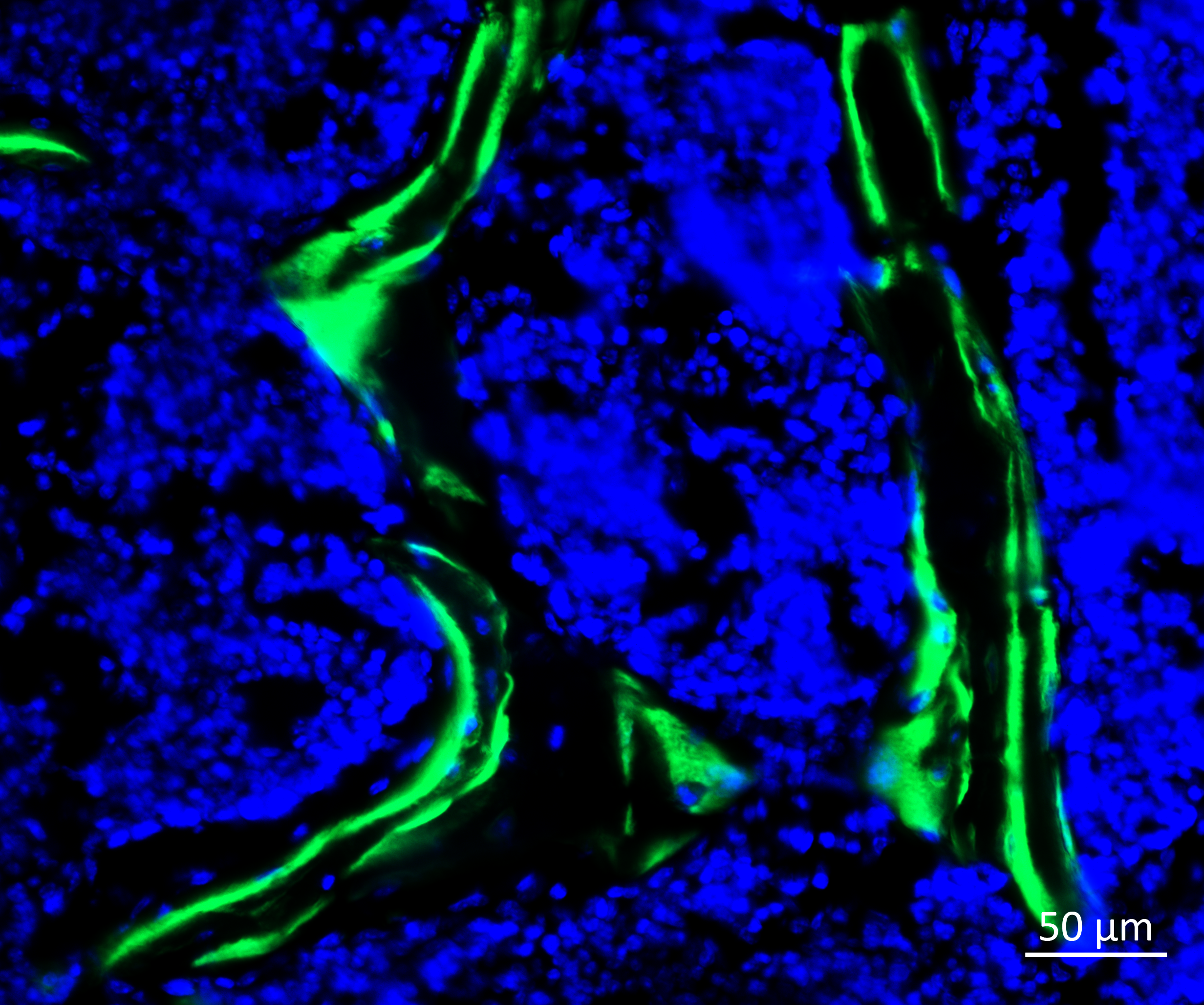

Supplement: Supplementary file 10 — Source data Fig. 8 [file 44319_2024_255_MOESM10_ESM.zip › Figure 8/8S/Double labeling_OVX-200mg.tif]

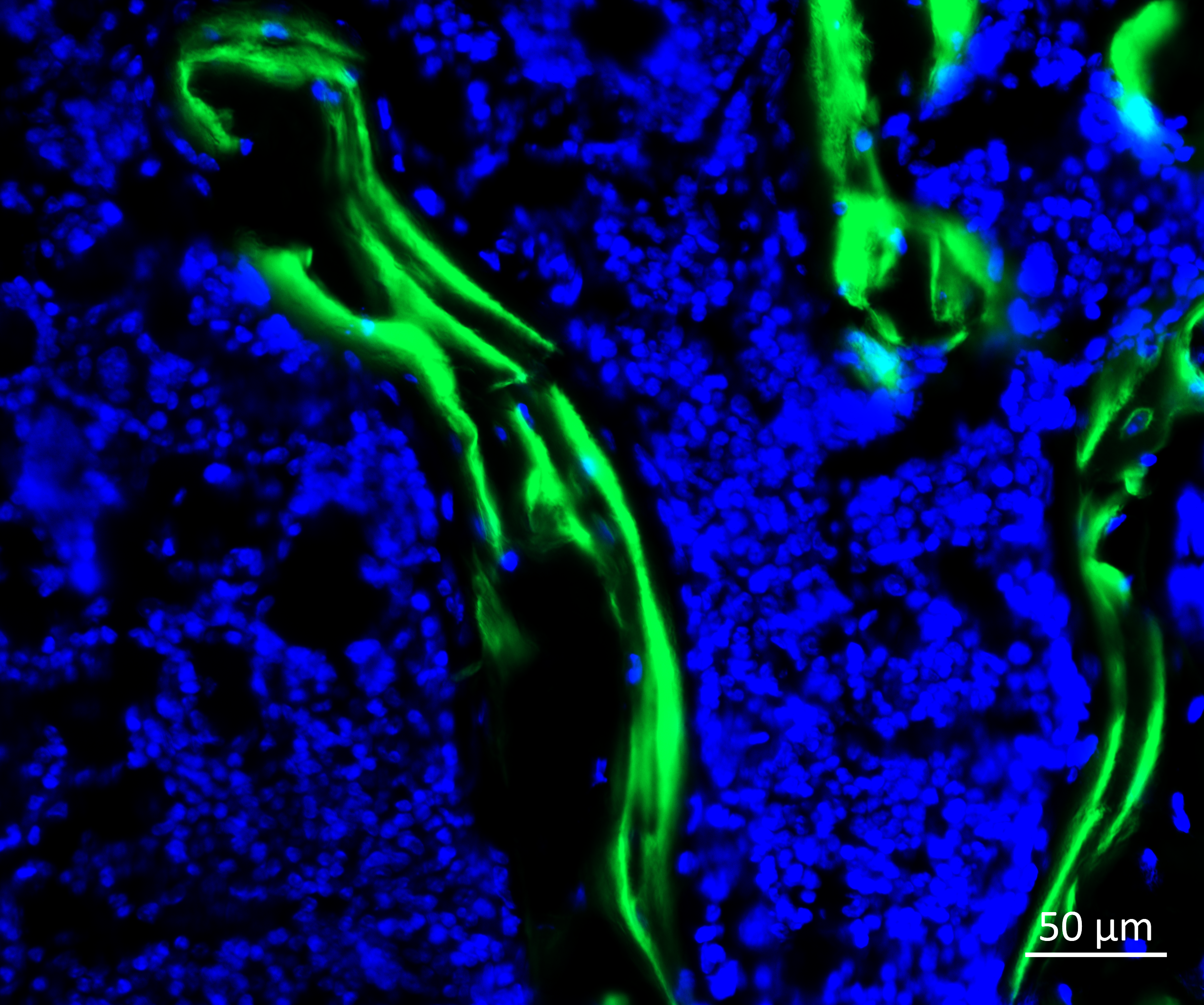

Supplement: Supplementary file 10 — Source data Fig. 8 [file 44319_2024_255_MOESM10_ESM.zip › Figure 8/8T/Double labeling_400mg.tif]
