# Supplementary material for: Glutaminolysis provides nucleotides and amino acids to regulate osteoclast differentiation in mice
Source: EMBO Rep. 2024 Sep 13;25(10):4515–41. doi: 10.1038/s44319-024-00255-x (PMC11467445; doi:10.1038/s44319-024-00255-x)
Supplement: Supplementary file 11 — Expanded View Figures [file 44319_2024_255_MOESM11_ESM.pdf]

Expanded View Figures

Figure EV1. The transcriptomic and metabolic changes during osteoclastogenesis.

(**A**, **B**) Gene Ontology pathway analysis of transcripts enriched in BMM (**A**) or mOC (**B**). (**C**) Volcano plots showing metabolites that differed significantly between BMM and mature osteoclast (mOC) ($n = 4$). Amino acids are colored red, and nucleotides are colored blue. (**D**) Metabolic pathway analysis (based on the Human Metabolome Database, HMDB) showing pathways enriched in mOC. (**E–H**) Graphical depiction of the intracellular abundance of select nucleotides in BMM, pOC, and mOC as determined by mass spectrometry ($n = 4$ independent experiments). (**I**) Metabolic pathways enriched in BMM. (**J**) Graphical depiction of the intracellular abundance of ATP ($n = 4$ independent experiments). (**K–N**) Graphical depiction of the intracellular abundance of select amino acids (alanine, glutamine, proline, and glutamate) in BMM, pOC, and mOC as determined by mass spectrometry ($n = 4$ independent experiments). Data are shown as mean ± SD. Two-tailed Student's unpaired *t* test (**A–D**, and **I**). 1-way ANOVA (**E–H**, **J**, **K–N**).

 © The Author(s)

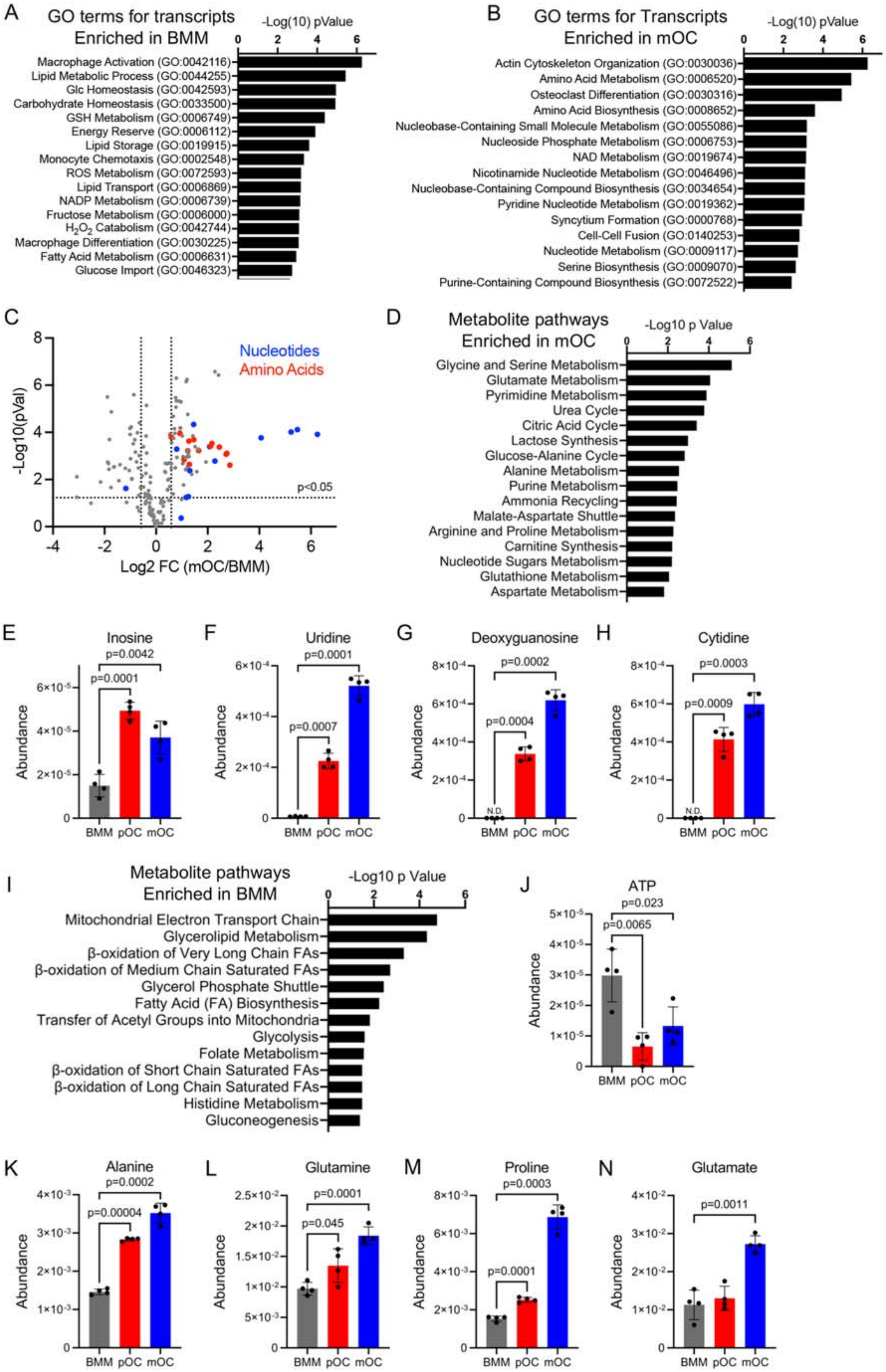
A
GO terms for transcripts Enriched in BMM
-Log(10) pValue
0 2 4 6
Macrophage Activation (GO:0042116)
Lipid Metabolic Process (GO:0044255)
Glc Homeostasis (GO:0042593)
Carbohydrate Homeostasis (GO:0033500)
GSH Metabolism (GO:0006749)
Energy Reserve (GO:0006112)
Lipid Storage (GO:0019915)
Monocyte Chemotaxis (GO:0002548)
ROS Metabolism (GO:0072593)
Lipid Transport (GO:0006869)
NADP Metabolism (GO:0006739)
Fructose Metabolism (GO:0006000)
H_2O_2 Catabolism (GO:0042744)
Macrophage Differentiation (GO:0030225)
Fatty Acid Metabolism (GO:0006631)
Glucose Import (GO:0046323)
B
GO terms for Transcripts Enriched in mOC
-Log(10) pValue
0 2 4 6
Actin Cytoskeleton Organization (GO:0030036)
Amino Acid Metabolism (GO:0006520)
Osteoclast Differentiation (GO:0030316)
Amino Acid Biosynthesis (GO:0008652)
Nucleobase-Containing Small Molecule Metabolism (GO:0055086)
Nucleoside Phosphate Metabolism (GO:0006753)
NAD Metabolism (GO:0019674)
Nicotinamide Nucleotide Metabolism (GO:0046496)
Nucleobase-Containing Compound Biosynthesis (GO:0034654)
Pyridine Nucleotide Metabolism (GO:0019362)
Syncytium Formation (GO:0000768)
Cell-Cell Fusion (GO:0140253)
Nucleotide Metabolism (GO:0009117)
Serine Biosynthesis (GO:0009070)
Purine-Containing Compound Biosynthesis (GO:0072522)
C
Nucleotides
Amino Acids
-Log10(pVal)
p<0.05
Log2 FC (mOC/BMM)
D
Metabolite pathways Enriched in mOC
-Log10 p Value
Glycine and Serine Metabolism
Glutamate Metabolism
Pyrimidine Metabolism
Urea Cycle
Citric Acid Cycle
Lactose Synthesis
Glucose-Alanine Cycle
Alanine Metabolism
Purine Metabolism
Ammonia Recycling
Malate-Aspartate Shuttle
Arginine and Proline Metabolism
Carnitine Synthesis
Nucleotide Sugars Metabolism
Glutathione Metabolism
Aspartate Metabolism
E
Inosine
p=0.0042
p=0.0001
Abundance
BMM pOC mOC
F
Uridine
p=0.0001
p=0.0007
G
Deoxyguanosine
p=0.0002
p=0.0004
N.D.
H
Cytidine
p=0.0003
p=0.0009
N.D.
I
Metabolite pathways Enriched in BMM
-Log10 p Value
Mitochondrial Electron Transport Chain
Glycerolipid Metabolism
β-oxidation of Very Long Chain FAs
β-oxidation of Medium Chain Saturated FAs
Glycerol Phosphate Shuttle
Fatty Acid (FA) Biosynthesis
Transfer of Acetyl Groups into Mitochondria
Glycolysis
Folate Metabolism
β-oxidation of Short Chain Saturated FAs
β-oxidation of Long Chain Saturated FAs
Histidine Metabolism
Gluconeogenesis
J
ATP
p=0.023
p=0.0065
K
Alanine
p=0.0002
p=0.00004
L
Glutamine
p=0.0001
p=0.045
M
Proline
p=0.0003
p=0.0001
N
Glutamate
p=0.0011

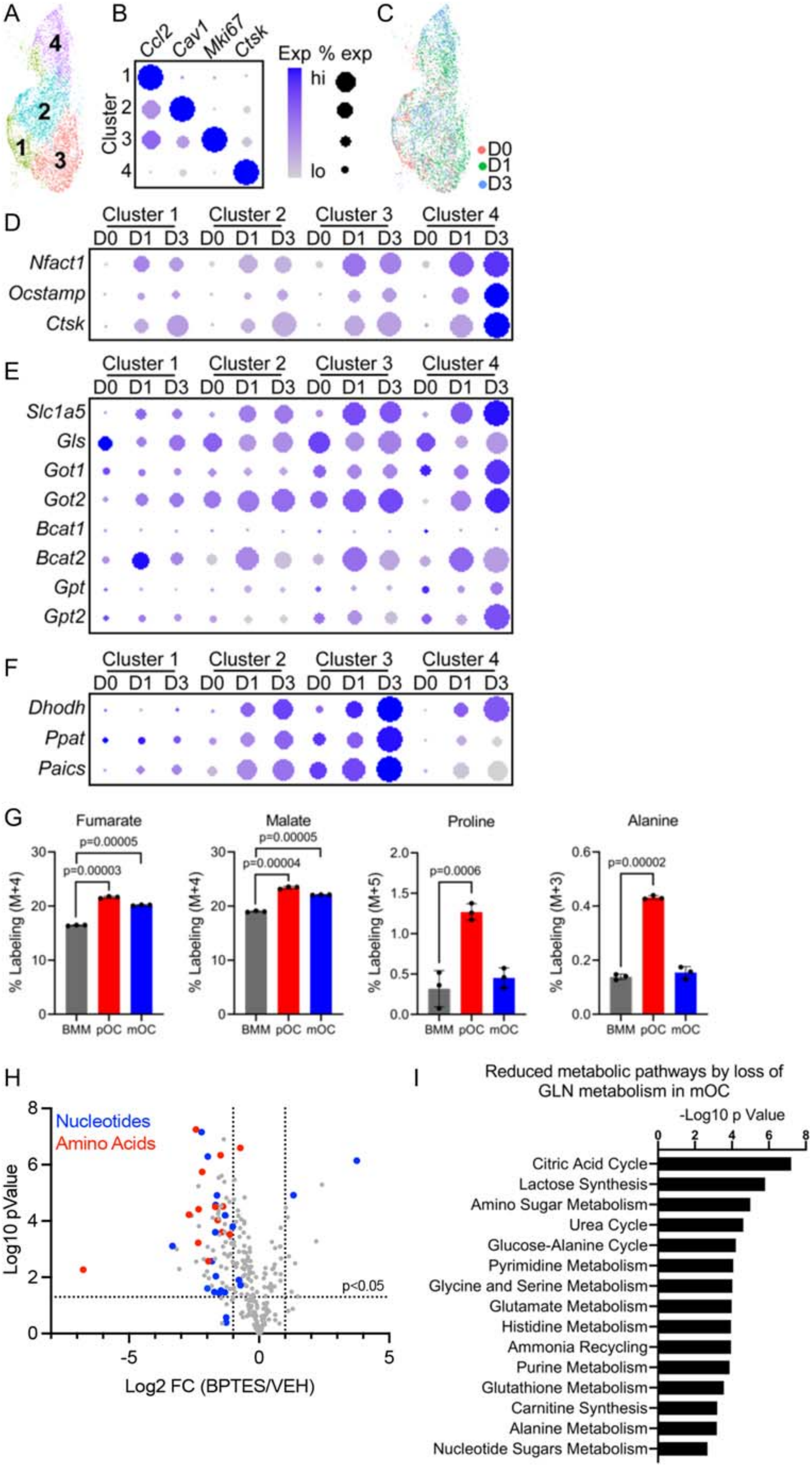
A
B
Ccl2 Cav1 Mki67 Ctsk
Cluster
Exp % exp
hi
lo
C
D0
D1
D3
D
Cluster 1 Cluster 2 Cluster 3 Cluster 4
D0 D1 D3 D0 D1 D3 D0 D1 D3 D0 D1 D3
Nfact1
Ocstamp
Ctsk
E
Slc1a5
Gls
Got1
Got2
Bcat1
Bcat2
Gpt
Gpt2
F
Dhodh
Ppat
Paics
G
Fumarate
Malate
Proline
Alanine
p=0.00005
p=0.00003
p=0.00004
p=0.0006
p=0.00002
% Labeling (M+4)
% Labeling (M+5)
% Labeling (M+3)
BMM pOC mOC
H
Nucleotides
Amino Acids
Log10 pValue
p<0.05
Log2 FC (BPTES/VEH)
I
Reduced metabolic pathways by loss of GLN metabolism in mOC
-Log10 p Value
Citric Acid Cycle
Lactose Synthesis
Amino Sugar Metabolism
Urea Cycle
Glucose-Alanine Cycle
Pyrimidine Metabolism
Glycine and Serine Metabolism
Glutamate Metabolism
Histidine Metabolism
Ammonia Recycling
Purine Metabolism
Glutathione Metabolism
Carnitine Synthesis
Alanine Metabolism
Nucleotide Sugars Metabolism

 © The Author(s)

Figure EV2. Glutaminolysis is activated during osteoclastogenesis to support metabolic reprogramming.

(**A**) UMAP visualization of 4 clusters during osteoclastogenesis in the osteoclast culture system (Day 0–3 with RANKL). (**B**) Dot plot showing cluster marker genes. (**C**) UMAP visualization colored by days post RANKL stimulation. (**D**) Average osteoclast marker gene expression in 4 clusters. (**E**) The expression of genes associated with glutaminolysis in 4 clusters. (**F**) The expression of genes associated with nucleotide biosynthesis in 4 clusters. (**G**) Graphical depiction showing the percent labeling of fumarate, malate, proline, and alanine from [U-^{13}C]-glutamine in BMM, pOC, and mOC ($n = 3$ independent experiments). (**H**) Volcano plot showing metabolites that differed significantly between BPTES (10 μM)- and vehicle-treated mOC ($n = 4$). (**I**) Metabolic pathway analysis showing reduced pathways in BPTES-treated mOC. Data are shown as mean ± SD. One-way ANOVA (**G**). Two-tailed Student's unpaired *t* test (**H**, **I**).

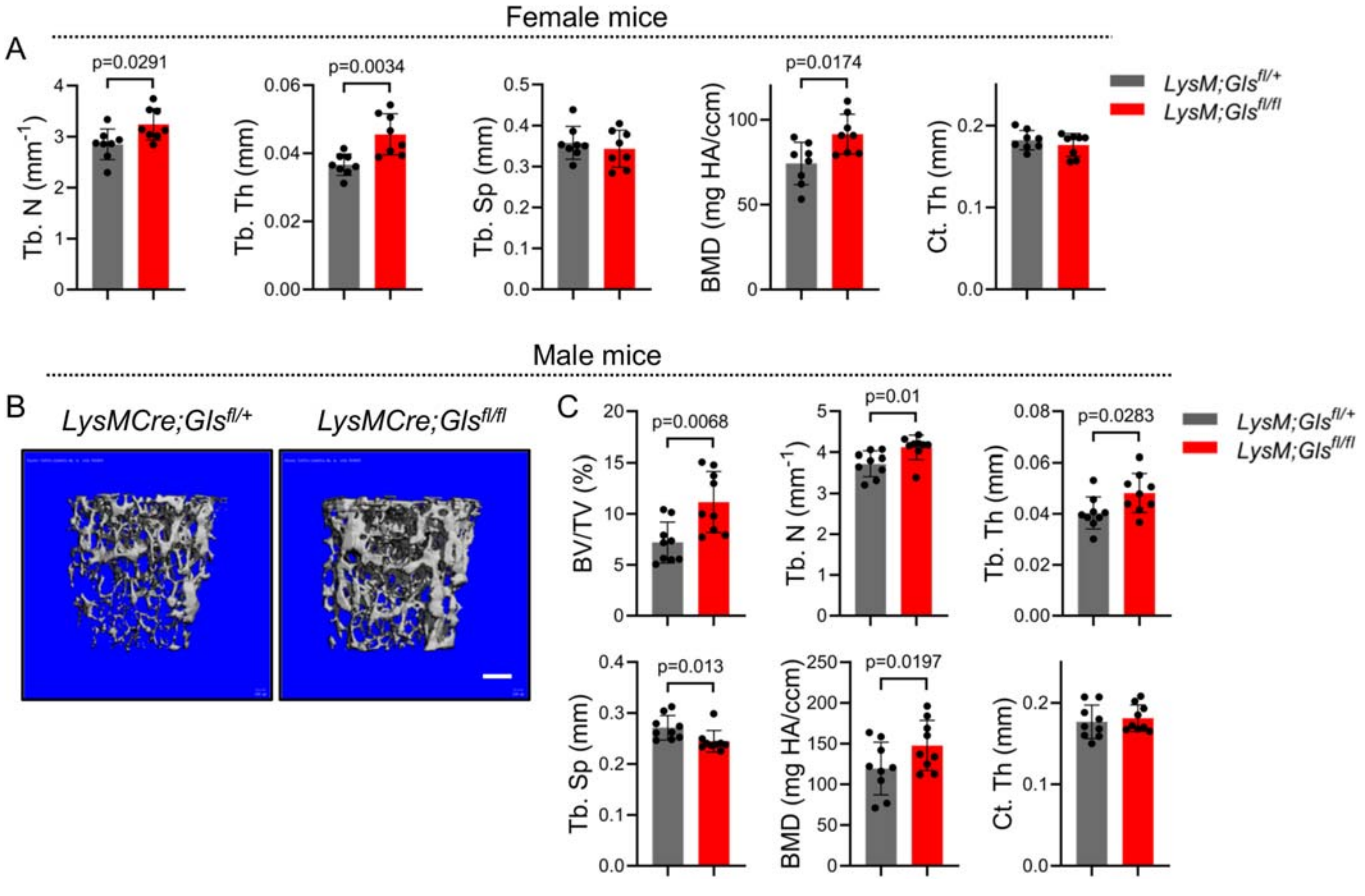

Figure EV3. *Gls* ablation in myeloid lineage cells increases bone mass in both male and female mice.

(**A**) µCT parameters of trabecular bone in the distal femurs of 4-month-old WT and *LysM;Gls*$^{fl/fl}$ female mice ($n = 8$ mice). (**B**, **C**) Representative µCT images (**B**) and µCT parameters of trabecular bone in the distal femurs of 4-month-old WT and *LysM;Gls*$^{fl/fl}$ male mice ($n = 9$ mcie). Scale bar: 200 µm. Data are shown as mean ± SD. Two-tailed Student's paired *t* test (**A** and **C**).

 © The Author(s)

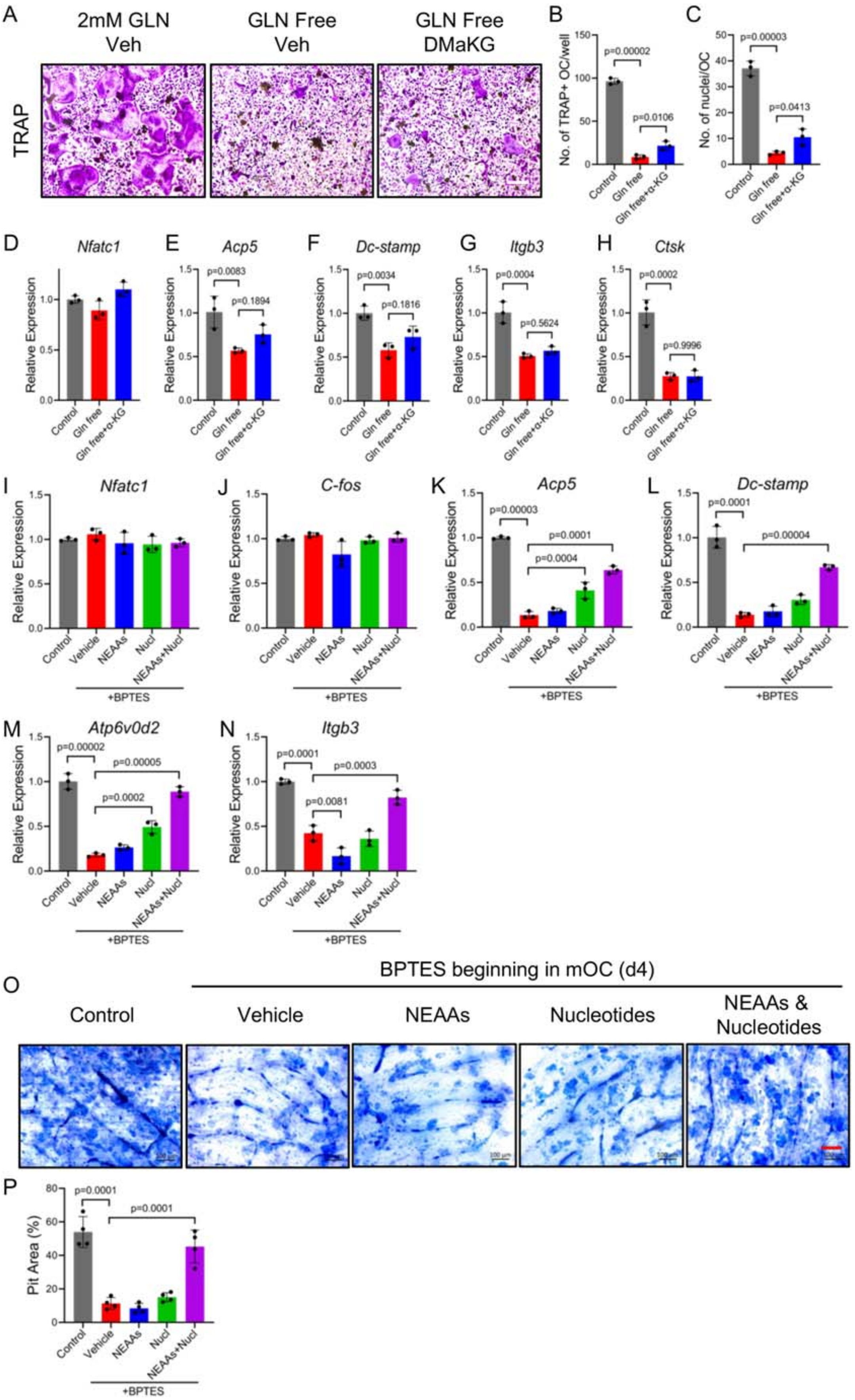
A
2mM GLN Veh
GLN Free Veh
GLN Free DMaKG
TRAP
B
No. of TRAP+ OC/well
p=0.00002
p=0.0106
Control
Gln free
Gln free+α-KG
C
No. of nuclei/OC
p=0.00003
p=0.0413
D
Nfatc1
E
Acp5
p=0.0083
p=0.1894
F
Dc-stamp
p=0.0034
p=0.1816
G
Itgb3
p=0.0004
p=0.5624
H
Ctsk
p=0.0002
p=0.9996
Relative Expression
I
Nfatc1
J
C-fos
K
Acp5
p=0.00003
p=0.0001
p=0.0004
L
Dc-stamp
p=0.0001
p=0.00004
M
Atp6v0d2
p=0.00002
p=0.00005
p=0.0002
N
Itgb3
p=0.0001
p=0.0003
p=0.0081
Control
Vehicle
NEAAs
Nucl
NEAAs+Nucl
+BPTES
O
BPTES beginning in mOC (d4)
Control
Vehicle
NEAAs
Nucleotides
NEAAs & Nucleotides
P
Pit Area (%)
p=0.0001
p=0.0001

Figure EV4. Glutaminolysis provides amino acids and nucleotides, but not αKG, to regulate osteoclastogenesis.

(**A–H**) The effect of supplementing dimethyl-α-ketoglutarate (DMαKG) on osteoclast differentiation as measured by TRAP staining (**A**, $n = 5$, scale bar: 500 µm) or qPCR analysis ($n = 3$ independent experiments) (**E–H**). (**C**) Quantification of TRAP-positive multi-nuclei cells from (**A**). (**D**) Quantification of the number of nuclei per TRAP-positive osteoclast from (**A**). (**I–P**) The effect of supplementing non-essential amino acids (NEAAs) and/or nucleotides (Nucl) on osteoclast differentiation and bone resorption as measured by qPCR analysis of osteoclast marker gene mRNA expression ($n = 3$ independent experiments) (**I–N**). (**O–P**) The effect of supplementing non-essential amino acids (NEAA) and/or nucleotides beginning at day 4 on bone resorption as measured by pit assay. Scale bar: 100 µm. (**P**) Quantification of the resorption pit area from **O** ($n = 4$ independent experiments). Data are shown as mean ± SD. One-way ANOVA (**B–H**), two-way ANOVA (**I–N**, **P**).

 © The Author(s)

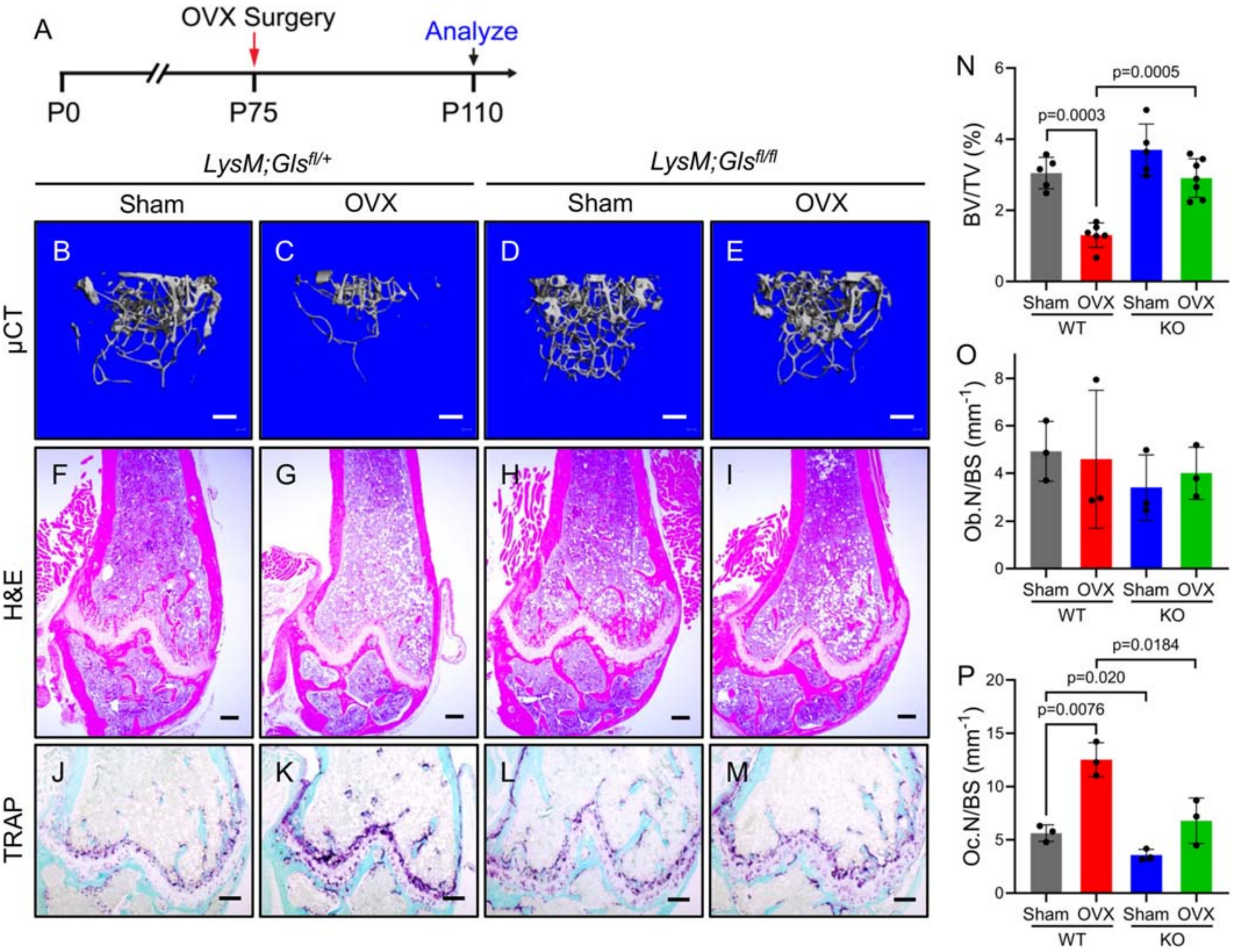

Figure EV5. Genetically targeting glutaminolysis prevents OVX-induced bone loss.

(**A**) Schematic showing the experimental design. (**B–M**) Representative images of μCT (**B–E**, scale bar: 200 μm), H&E staining (**F–I**, scale bar: 100 μm), and TRAP staining (**J–M**, scale bar: 100 μm) of trabecular bone in the distal femurs of WT and *LysM;Gls$^{fl/fl}$* female mice subjected to OVX or sham surgery ($n = 5$–7). (**N**) The calculated BV/TV from μCT images ($n = 5$–7 mice). (**O**) Osteoblast number per bone surface (Ob.N/BS) quantified from H&E stains ($n = 3$ mice). (**P**) Osteoclast number per bone surface (Oc.N/BS) quantified from TRAP stains ($n = 3$ mice). Data are shown as mean ± SD. Two-way ANOVA (**N–P**).
